# High prevalence and genetic diversity of *Treponema paraluisleporidarum* isolates in European lagomorphs

**DOI:** 10.1128/spectrum.01774-23

**Published:** 2023-12-14

**Authors:** Sascha Knauf, Linda Hisgen, Erik O. Ågren, Alexander M. Barlow, Marcus Faehndrich, Ulrich Voigt, Luisa Fischer, Linda Grillová, Luisa K. Hallmaier-Wacker, Marja J. L. Kik, Jana C. Klink, Jitka Křenová, Antonio Lavazza, Simone Lüert, Markéta Nováková, Darina Čejková, Carlo Pacioni, Tiziana Trogu, David Šmajs, Christian Roos

**Affiliations:** 1 Institute of International Animal Health/One Health, Friedrich-Loeffler-Institut, Federal Research Institute for Animal Health, Greifswald, Germany; 2 Infection Biology Unit, Deutsches Primatenzentrum GmbH, Leibniz Institute for Primate Research, Göttingen, Germany; 3 Professorship for International Animal Health/One Health, Faculty of Veterinary Medicine, Justus Liebig University, Giessen, Germany; 4 Department of Pathology and Wildlife Diseases, National Veterinary Institute, Uppsala, Sweden; 5 Wildlife Network for Disease Surveillance, Bristol Veterinary School, Langford, Somerset, United Kingdom; 6 Institute for Terrestrial and Aquatic Wildlife Research, University of Veterinary Medicine Hanover, Foundation, Hanover, Germany; 7 Wildlife Research Institute, State Agency for Nature, Environment and Consumer Protection North Rhine-Westphalia, Bonn, Germany; 8 Department of Biology, Masaryk University, Brno, Czechia; 9 Pathology Division, Department of Biomedical Health Sciences, Veterinary Medicine, Utrecht University, Utrecht, the Netherlands; 10 Department of Animal Health and Welfare – Virology Unit, Istituto Zooprofilattico Sperimentale della Lombardia e dell'Emilia Romagna, Brescia, Italy; 11 Department of Biomedical Engineering, Brno University of Technology, Brno, Czechia; 12 Department of Environment, Land, Water and Planning, Arthur Rylah Institute for Environmental Research, Heidelberg, Victoria, Australia; 13 Environmental and Conservation Sciences, Murdoch University, Murdoch, Australia; 14 Primate Genetics Laboratory, Deutsches Primatenzentrum GmbH, Leibniz Institute for Primate Research, Göttingen, Germany; 15 Gene Bank of Primates, Deutsches Primatenzentrum GmbH, Leibniz Institute for Primate Research, Göttingen, Germany; McGill University, Ste-Anne-de-Bellevue, Quebec, Canada

**Keywords:** spirochetes, European brown hare, *Lepus*, rabbit, *Oryctolagus*, syphilis, *Treponema pallidum*, One Health

## Abstract

The bacterium *Treponema paraluisleporidarum* causes syphilis in lagomorphs. In a set of 1,095 samples from four species—European brown hare, mountain hare, Corsican hare, and European rabbit—we tested for infection and genotyped the strains that infect wild lagomorphs. Samples originate from Sweden, the Netherlands, the United Kingdom, Germany, the Czech Republic, and Italy. The phylogenetic analyses of two informative gene targets (*tp0488* and *tp0548*) showed high genetic diversity among the lagomorph-infecting treponemes. More specifically, we found a high number of nucleotide variants and various short repeat units in the *tp0548* locus that have not been described for human syphilis and primate yaws causing *Treponema pallidum*. While the functional aspect of these short repeat units remains subject to ongoing investigations, it likely enables the pathogen to better survive in its lagomorph host. Our data did not support any geographic clustering, which is equally reflected in the host population genetics as shown by mitochondrial genome data corresponding to the sampled lagomorph populations. This is unexpected and in contrast with what has been shown for nonhuman primate infection with *T. pallidum*. In the future, the combination of multi-locus sequence typing and whole genome data from modern and ancient samples from a wide geographic range and multiple lagomorph species will contribute to a better understanding of the epidemiology and evolutionary path of lagomorph-infecting treponemes. In conclusion, our current study demonstrates widespread infection and a high genetic variation of the syphilis-causing pathogen in a higher number of positively PCR-tested European lagomorphs (*n* = 302/1,095).

**IMPORTANCE:**

Syphilis is an ancient disease of humans and lagomorphs caused by two distinct but genetically closely related bacteria (>98% sequence identity based on the whole genome) of the genus *Treponema*. While human syphilis is well studied, little is known about the disease in the lagomorph host. Yet, comparative studies are needed to understand mechanisms in host–pathogen coevolution in treponematoses. Importantly, *Treponema paraluisleporidarum*–infected hare populations provide ample opportunity to study the syphilis-causing pathogen in a naturally infected model population without antibiotic treatment, data that cannot be obtained from syphilis infection in humans. We provide data on genetic diversity and are able to highlight various types of repetitions in one of the two hypervariable regions at the *tp0548* locus that have not been described in the human syphilis-causing sister bacterium *Treponema pallidum* subsp. *pallidum*.

## INTRODUCTION

Human and lagomorph syphilis is caused by different but genetically closely related spirochete bacteria. While studies on modern (human) syphilis lineages controversially discuss a common ancestor in the 1700s (1), the evolution of all three *Treponema pallidum* (*TP*) subspecies—subsp. *pallidum* (*TPA*) causing syphilis, subsp. *endemicum* causing bejel, and subsp. *pertenue* causing yaws (*TPE*)—and the genetically closely related lagomorph syphilis-causing bacterium *Treponema paraluisleporidarum* ecovar Lepus (*TP*eL) in hares and *T. paraluisleporidarum* ecovar Cuniculus (*TP*eC) in rabbits remains elusive. While treponematoses have a several hundred-year-long history in humans (2, 3), yaws is also known to infect nonhuman primates (4). The taxonomic status of the genus *Treponema* and, in particular, the nomenclature of the lagomorph-infecting treponemes is still unresolved. While the valid species name is still *Treponema paraluiscuniculi* (5), several peer-reviewed publications have picked up the introduction of the name *T. paraluisleporidarum* based on observed differences in infection experiments (6). Although the use of the newly suggested species name is premature according to bacterial taxonomy, we will consistently make use of it in this publication to avoid confusion with our previous publications.

Only a single complete genome of a rabbit-infecting laboratory-maintained strain from the USA has been whole genome-sequenced until today (7). Comparative studies showed that this strain, *TP*eC strain Cuniculi A, has a 98.1% whole genome identity to the human syphilis-causing *TPA* strain Nichols (7, 8). Although its genome is slightly smaller compared to that of the human-infecting syphilis bacterium, there is a general genome synteny (7). Yet, all information on genomic deletions, insertions, or changes that likely code for the lagomorph host specificity is derived from the single published *TP*eC strain Cuniculi A genome (7, 9). Consequently, the basis for human pathogenicity cannot be identified unless more *Treponema* genomes of lagomorph origin are analyzed. For that reason, the lagomorph*–Treponema* system provides ample opportunity to study the epidemiology and evolution of a pathogen that naturally models human syphilis under unbiased conditions, e.g., in the absence of antimicrobial pressure. Comparative genome data will allow further insight into the evolution of virulence factors and host adaptation equally relevant to the sister bacterium *T. pallidum*.

Our previous studies have demonstrated anti-*T*. *paraluisleporidarum* antibodies and the presence of the bacterium in several European brown hare (EBH; *Lepus europaeus*) populations (10 – 12). Serology and quantitative PCR were, however, unable to distinguish infection with *TP*eL, *TP*eC, or the genetically closely related *TP*. We note here that the latter is not known to infect wild lagomorphs, but rabbits are traditionally used to cultivate human treponemes *in vivo,* which highlights the ability of *TP* to adapt and survive in the lagomorph host (13). In this study and prior to whole genome sequencing, we were interested in the epidemiology and the genetic diversity of treponemes infecting European lagomorphs. In light of the previously reported widespread infection in the wild lagomorph host, which argues for a well-established disease in European hares, we hypothesized that hare-infecting strains have a strain diversity as high as the one seen in a comparative wild host–pathogen system—African nonhuman primates infected with the sister bacterium *TPE* (14). We predicted a geographic clustering of strains isolated from structurally connected hare populations. We used the naturally occurring *Treponema* infection in nonhuman primates as a direct comparison since the infection in wild lagomorphs is equally not under selection pressure from antibiotic treatment (4) and has multiple host species involved. Due to the high genetic similarity of human and nonhuman primate-infecting *TP* and hare and rabbit-infecting *TP*eL/C [98.1% genome identity (7)], we applied a multi-locus sequence typing (MLST) system for confirmation of infection and to strain-type treponemes that were originally designed for *TPE* infection in nonhuman primates (14).

## MATERIALS AND METHODS

### Study design, sampling locations, and animals

**Fig 1:**
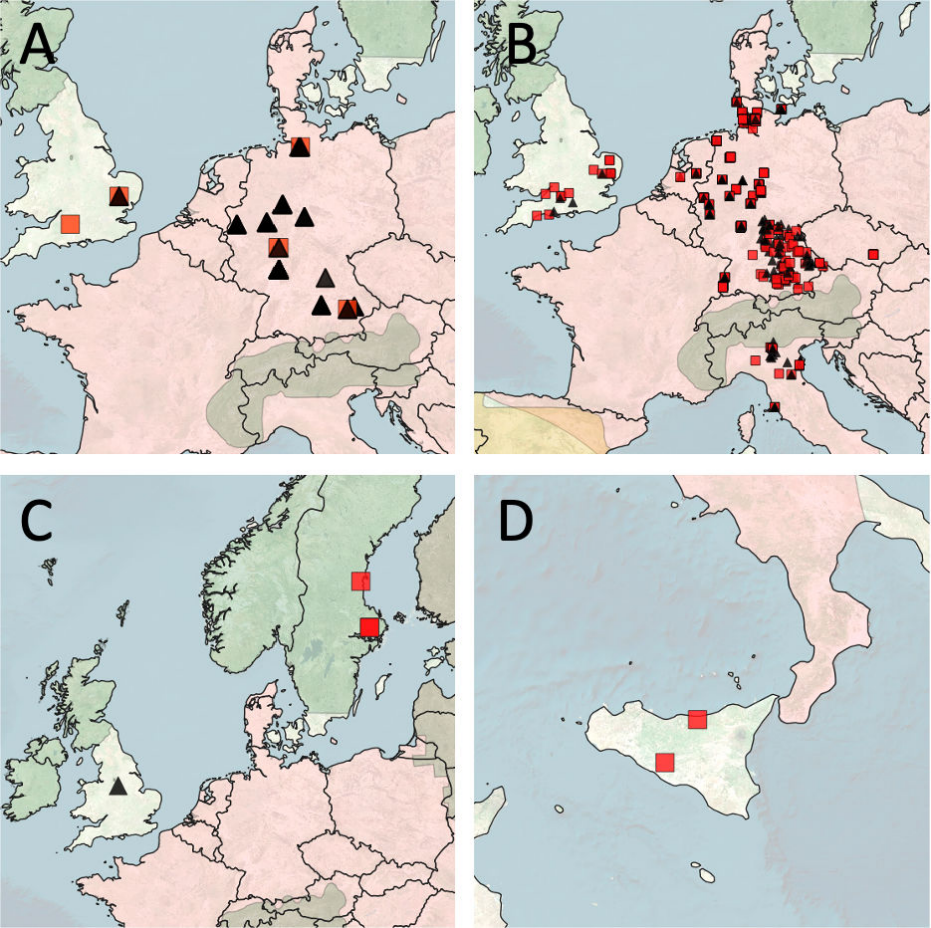
Map of Europe showing the geographic origin and the infection status of our samples based on the host species: (**A**) European rabbit (*Oryctolagus cuniculus*), (**B**) European brown hare (*Lepus europaeus*), (**C**) mountain hare (*Lepus timidus*), and (**D**) Corsican hare (*Lepus corsicanus*). Red squares indicate positive, and black triangles represent negative tested animals. Colored geographic range overlays provide information on the extant (not introduced) distribution of the different lagomorph species according to the International Union for Conservation of Nature Red List of Threatened Species (https://www.iucnredlist.org). *L. europaeus*, in addition, has been documented as a resident of the UK, Ireland, and Sweden [range expansion not shown (15)]. Green = mountain hare; red = European brown hare; yellow = Iberian hare (*Lepus granatensis*). The map was created in QGIS v. 3.20 using Bing Aerial (http://ecn.t3.tiles.virtualearth.net/tiles/a{q}.jpeg?g = 1) as a map source with TM World Borders 0.3 overlay (https://thematicmapping.org/downloads/world_borders.php). Microsoft product screenshots reprinted with permission from Microsoft Corporation.

We tested a total of 1,095 lagomorphs from legally hunted or otherwise deceased (e.g., freshly dead) EBHs (*L. europaeus*, *n* = 1,042), mountain hares (*Lepus timidus*, *n* = 5), Corsican hares (*Lepus corsicanus*, *n* = 2), and European rabbits (*Oryctolagus cuniculus*, *n* = 39) that were opportunistically and randomly collected between 2016 and 2021 in six European countries [Germany (*n* = 938), Sweden (*n* = 4), England (*n* = 25), Italy (*n* = 81), the Netherlands (*n* = 32), and the Czech Republic (*n* = 15); Fig. 1]. The 39 rabbit samples included six euthanized pet rabbits (*O. cuniculus*) and one sample from a live domestic rabbit that was presented with syphilitic lesions in a veterinary clinic. In the latter, swabs were taken purposely for diagnostic reasons.

### Sampling

We swabbed the vagina of female lagomorphs using sterile polyester-tipped swabs (AF.022, abf diagnostics, Kranzberg, Germany) and removed the corpus penis using a sterile scalpel blade in males. In animals with crusty lesions, we sampled the affected skin area using sterile scalpel blades. All samples were stored in 2-mL safe seal tubes (Sarstedt, Nürnbrecht, Germany) containing 500 µL of sterile filtered custom-made lysis buffer (10 mM Tris-HCl, pH 8.0; 0.1 M EDTA, pH 8.0; 0.5% SDS). Samples were frozen at −20°C until further processing.

### DNA extraction

DNA was extracted from swabs and tissue material using the QIAamp DNA Mini Kit (Qiagen, Hilden, Germany) following the manufacturer’s guidance with some minor modifications. Briefly, we extracted DNA from swabs and tissue samples according to the protocols published by Chuma et al. (16) and Hisgen et al. (11), respectively. Subsequently, glycogen precipitation was performed to clean and concentrate the DNA. The method followed the procedure described by Knauf et al. (17). We measured the DNA yield using a NanoDrop photometer (Thermo Fisher Scientific, Darmstadt, Germany).

### Polymerase chain reactions

#### Host genus confirmation

Samples that were received from third parties (e.g., hunters) were subject to host genus confirmation to ensure the correctness of species assignment. For this reason, we amplified a 1,486-bp-long region of the immunoglobulin heavy chain gene using two independent PCRs (IGHGCH2 and IGHG hinge regions) to differentiate lagomorphs on the genus level (18). Briefly, the 50-µL reaction contained 25-µL 2× Phanta Max Master Mix (Vazyme Biotech Co. Ltd., Nanjing, China), 19 µL RNase-free water, 2 µL of the respective 10-µM primers, and template DNA [511.1 ± 600.1 (mean ± SD, *n* = 1,081)]. Cycling conditions were identical for both PCRs: 3-min initial denaturation at 95°C followed by 40 amplification cycles of 15 sec at 95°C and 15 sec at 60°C and an elongation phase at 72°C for 30 sec, followed by a post-extension step at 72°C for 5 min.

#### Treponema multi-locus sequence typing

In our previous work, we identified two variable gene loci for strain typing of *TPE* (*tp0488* and *tp0548*) in nonhuman primates (14), which we adapted for use in our lagomorph samples. We note here the close genetic relationship between *TPE* and *TP*eC, which is over 98% based on the whole genome (7, 19), 97.8% for the *tp0488* gene, and 90.3% for the *tp0548* gene with all amplification primer binding sites being 100% conserved (7).

##### tp0488

The PCR amplifies a ~830-bp region of the methyl-accepting chemotaxis protein 2 gene (*mcp*2). We checked previously published primers (14) for compatibility with the published *TP*eC strain Cuniculi A (GenBank CP002103.1) and amplified the gene target as described previously (14). Briefly, the 51-µL reaction volume comprised 45-µL Platinum PCR Super Mix High Fidelity (Thermo Fisher Scientific, Darmstadt, Germany), 2 µL of each 10-µM primer, and template DNA [511.1 ± 600.1 (mean ± SD, *n* = 1,081)], respectively. The amplification was performed with a SensoQuest Thermocycler (SensoQuest, Goettingen, Germany) applying the following conditions: 2 min pre-denaturation at 94°C followed by 80 cycles of 15 sec at 94°C, 15 sec at 59°C, and 60 sec at 68°C.

##### tp0548

We used a nested PCR to amplify the target sequence of the *tp0548* gene (encoding for a predicted outer membrane protein), using primers and cycling conditions as published elsewhere (14) with the only exception of using a different polymerase. Briefly, the 50-µL reaction solution contained 25-µL 2× Phanta Max Master Mix (Vazyme Biotech Co. Ltd., Nanjing, China), 19-µL RNase-free water, 2 µL of the respective 10-µM primers, and template DNA. The initial amplification of a 1,567-bp product was followed by a second PCR (nested) to amplify the final target sequence of ~1,070 bps. We used 2 µL of the first PCR reaction as input material. Cycling conditions for the first and nested PCR were 3-min initial denaturation at 95°C followed by 35 amplification cycles of 15 sec at 95°C and 15 sec at 48°C and an elongation phase at 72°C for 90 sec (first PCR) and 60 sec (nested PCR), respectively. Each PCR run ended with a post-extension step at 72°C for 5 min.

### Gel electrophoresis, DNA purification, and Sanger sequencing

All amplified samples were run on a 1.5% agarose gel, and PCR products of the correct size were extracted using the QIAquick Gel Extraction Kit (Qiagen, Hilden, Germany) following the manufacturer’s protocol. Subsequently, extracted DNA was sent for Sanger sequencing using the Microsynth Laboratory service (Microsynth, Göttingen, Germany). For the *tp0488* gene product and the IGHGCH2 and the IGHG hinge region amplicons, we utilized the respective forward primer for sequencing. The *tp0548* amplicons, however, were sequenced bidirectionally using the internal sequencing primers published elsewhere (20).

### Sanger sequencing data analysis

Sanger sequence data were evaluated, edited, and aligned using Geneious Prime 2021.2.2 (Biomatters Limited, Auckland, New Zealand) and 4Peaks sequence viewer (Nucleobytes B.V., Aalsmeer, the Netherlands). We compared sequence data to respective orthologs available in GenBank using BLAST search (https://blast.ncbi.nlm.nih.gov/Blast.cgi). *Treponema* sequence data were analyzed for positive gene selection following the tools and algorithm described by Maděránková et al. (21). Briefly, positively selected sites were determined from sequence alignments using (i) a codon-based site model implemented in the EasyCodeML package (22) and/or (ii) a mixed effect model of evolution using the hypothesis testing approach via the Datamonkey webserver (23, 24). For CodeML analysis, the phylogenetic trees were constructed using RAxML-NG tool (25). Phylogenetic trees and networks were constructed with IQ-TREE 2.0.7 (26) and MrBayes 3.2.7 (27), and the minimum spanning trees were inferred using the MSTree V2 algorithm within the GrapeTree program (28).

Maximum-likelihood trees in IQ-TREE were constructed with 1,000 ultrafast bootstrap replicates (29) and the best-fit model (GTR+F+I+G4) as obtained by IQ-Tree’s ModelFinder (30) according to the Bayesian Information Criterion. Tree reconstructions based on Bayesian inference in MrBayes and using the GTR+I+G model of sequence evolution were conducted with 1,000,000 generations with sampling every 100 generations and a burn-in of 25%. To check for convergence of all parameters and adequacy of the burn-in, we investigated the uncorrected potential scale reduction factor (31) as calculated by MrBayes. We used *T. pallidum* subsp. *endemicum* strain Iraq B (GenBank CP032303.1) as an outgroup to root our trees.

### Mitochondrial DNA amplification and high-throughput sequencing

We amplified mitochondrial (mt)-genomes of 95 randomly selected EBHs equally distributed across all sampling sites in Germany, England, Italy, the Netherlands, and the Czech Republic (*n* = 1–5 per site, *n* = 36 sites). Randomization was performed using Research Randomizer 4.0 (http://www.randomizer.org/). In an initial step, we performed two independent long-range PCRs using *Lepus europaeus*–specific primers covering the mt-genome range positions (reference genome GenBank accession number KY211031) 19-9464 (mtF1_lepus_S_Roos19 5′-AAA GCA AAG CAC TGA AAA TGC T and mtF1_lepus_AS_Roos19 5′-CCA AAA CTA ACT GAT TGG AAG T) and positions 8500-484 (mtF2_lepus_S_Roos19 5′-ATT AGT CCA ACA ACA GCC CTA and mtF2_lepus_AS_Roos19 5′-CTT AGC TAT CGT GAG TTC GAA). Primers were designed based on available mt-genome data in GenBank using Geneious Prime 2021.2.2 software. PCR reactions were adjusted to 50 µL and included the following components: 10-µL 5× PrimeSTAR GXL buffer (Takara Bio Europe SAS/Clontech Labs, Saint-Germain-en-Laye, France), 1-µL PrimeSTAR GXL DNA Polymerase (Takara Bio Europe SAS, Saint-Germain-en-Laye, France), 4-µL dNTP mixture (2.5 mM each), 28.5-µL RNase-free water, 2 µL of each 10-µM primer, and ~250 ng DNA (1–3.5 µL depending on DNA concentration). Cycling conditions were as follows: 35 cycles at 98°C for 10 sec, 59°C for 15 sec, and 68°C for 12 min. Subsequently, DNA amplicons were purified using SPRISelect magnetic beads (Beckman Coulter, Inc., Krefeld, Germany) followed by the determination of DNA concentration utilizing the Qubit 4.0 fluorometer (Thermo Fisher Scientific, Darmstadt, Germany). Next, we pooled 250 ng of both PCR products per individual and adjusted the final volume to 26 µL. Pooled PCR products were then enzymatically fragmented to an average size of 300–700 bps and subjected to next-generation library preparation using the NEBNext Ultra II FS DNA Library Prep Kit for Illumina (New England Biolabs, Frankfurt am Main, Germany) according to the manufacturer’s instructions in combination with the NEBNext Multiplex Oligos for Illumina (NEBE6440, 96 Unique Dual Index Primers plate) to ensure that all samples can be sequenced at once. Following the manufacturer’s guidance and prior to sequencing, library quantification was performed with the NEBNext Library Quant Kit for Illumina (New England Biolabs) run on a StepOnePlus Real-Time PCR System (Thermo Fisher Scientific, Darmstadt, Germany). In a final step, we normalized the samples to a concentration of 10 nM using the previously generated quantification data. Libraries were then pooled and sent to the NGS Integrative Genomics Core Unit (NIG, University Medical Center, Göttingen, Germany) for Illumina MiSeq sequencing (250 bp paired-end).

### High-throughput sequencing data analysis

Mt-genome assembly was conducted with the Geneious 11.1.3 package (https://www.geneious.com/). First, demultiplexed raw sequence reads were trimmed and quality-filtered with BBDuk 37.64 of the BBTools package (https://jgi.doe.gov/data-and-tools/bbtools/), and duplicate reads were removed with Dedupe 37.64 (BBTools package); for both steps, we applied standard settings. For mt-genome assembly, cleaned reads were mapped onto the reference mt-genome of *L. europaeus* (GenBank: NC_004028.1) using the Geneious assembler with standard settings. Newly produced mt-genomes were manually checked and then annotated with Geneious. For phylogenetic tree reconstruction, we added additional mt-genome sequences from EBHs from Sweden, Poland, Greece, Cyprus, and Turkey available in GenBank and aligned them as described above. Tree reconstructions based on the maximum-likelihood algorithm and Bayesian inference were performed as described above using IQ-TREE and MrBayes software.

## RESULTS

### *Treponema* infection and clinical manifestations

We tested 1,095 lagomorphs and obtained 302 positives by PCR [27.6%, at least one gene (*tp0488* or *tp0548*) positive; Table 1], but not all lagomorphs could be clinically inspected by our research team. Of those clinically examined (*n* = 531/1,095), only 20 animals (3.8%) had crusts and ulcerations in the face (*n* = 6) or genital region (*n* = 14). The latter includes the domestic rabbit presented in a veterinary clinic. All samples from animals with documented facial or genital lesions*—L. europaeus* = 13, *L. timidus* = 4, *L. corsicanus* = 2, and *O. cuniculus* = 1—were PCR-positive for *T. paraluisleporidarum* (Table S1).

**TABLE 1:** Overview of the infection status of the sampled lagomorphs

Host species	*N* sampled	Confirmed *TP*eC/L PCR positive^*a*^	PCR negative	*TP*eC/L PCR inconclusive^*b*^
*L. europaeus*	1,042	294	514	234
*Lepus timidus*	5	4	1	0
*Lepus corsicanus*	2	2	0	0
*Oryctolagus cuniculus*	39	1	35	3
*Oryctolagus cuniculus* f. *domestica*	7	1	6	0
Total	1,095	302	556	237

^*a*^ At least one gene (*tp0488* or *tp0548*) amplified and sequenced.

^*b*^ PCR product of correct size visible on agarose gel and low sequence quality or high background noise due to superimposed sequences.

### *Treponema paraluisleporidarum* multi-locus sequence typing

We obtained sequence results for the *tp0488* locus from 210 animals (Germany: *n* = 167/938, Sweden: *n* = 4/4, England: *n* = 7/25, Italy: *n* = 11/81, the Netherlands: *n* = 7/32, and Czech Republic: *n* = 14/15) and 285 animals (Germany: *n* = 241/938, Sweden: *n* = 4/4, England: *n* = 9/25, Italy: *n* = 9/81, the Netherlands: *n* = 7/32, and the Czech Republic: *n* = 15/15) for the *tp0548* gene locus (Tables S1 and S2). In 17 animals, we could only amplify the *tp0488* gene, and in 92 animals, we were only able to amplify the *tp0548* gene target.

**Fig 2:**
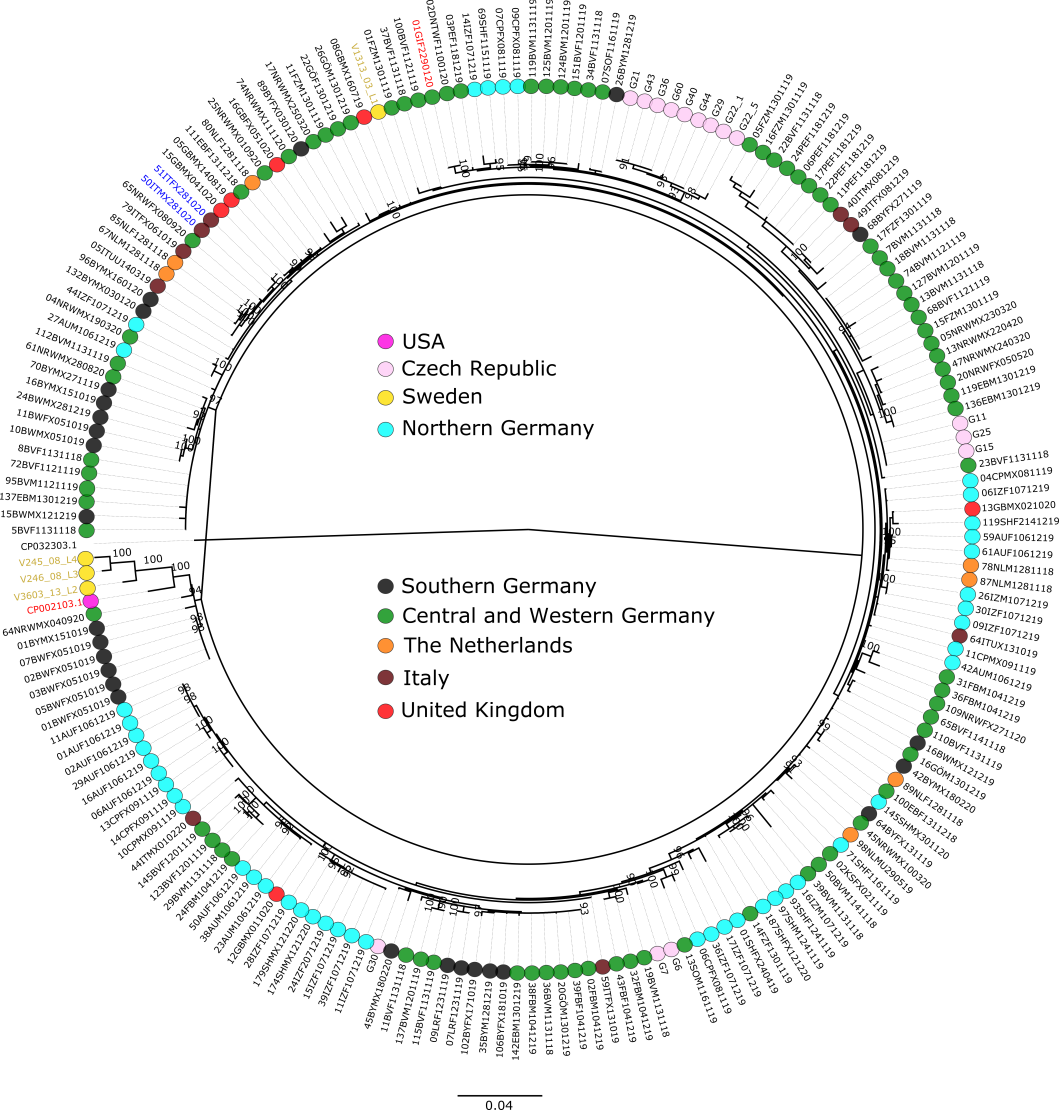
Maximum-likelihood tree based on the concatenated alignment (*tp0488* and *tp0548*). The tree was constructed with IQ-TREE with the best-fit model (GTR+F+R3) based on the Bayesian Information Criterion and 1,000 bootstrap replicates. We included 194 sequences, containing 156 parsimony-informative sites and 48 singletons. Only bootstrap values >90% are shown. The scale bar represents substitutions per nucleotide site. The text color indicates the host species: black = *Lepus europaeus*; yellow = *Lepus timidus*; blue = *Lepus corsicanus;* red = *Oryctolagus cuniculus*. The tree was rooted using *T. pallidum* subsp. *endemicum* strain Iraq B (GenBank CP032303.1).

Our sequencing efforts resulted in 210, 294, and 192 high-quality sequences for *tp0488*, *tp0548,* and the concatenated gene target sequences. These data were complemented with the reference sequence from the *TP*eC strain Cuniculi A (CP002103.1; locus tags *TPCCA_RS02365* and *TPCCA_RS02685*) and *T. pallidum* subsp. *endemicum* strain Iraq B (CP032303.1; locus tags TENDIB_0488 and TENDIB_0548). We generated a maximum-likelihood tree based on the concatenated sequences of the *tp0488* and *tp0548* genes and added the geographic origin as attributes, with samples grouped into Northern (Schleswig Holstein and northern part of Lower Saxony), Central and Western (southern part of Lower Saxony, Hesse, and North Rhine-Westphalia), and Southern (Bavaria and Baden-Wuerttemberg) Germany as well as the Netherlands, Italy, the Czech Republic, and the United Kingdom. Overall, there is no clustering of the samples according to their geographic origin (Fig. 2). While bootstrap support for nodes in the phylogenetic tree is generally low, there are some significantly supported distinctive features that are noteworthy. The tree exhibits an initial split into two clades of which one contains sequences obtained from EBHs sampled in Baden-Wuerttemberg and Bavaria (Southern Germany) and one EBH from North Rhine-Westphalia (Western Germany). In addition, the clade contains the *TP*eC reference strain Cuniculi A and three strains of mountain hares from Sweden. A sample from the fourth Swedish mountain hare (V1313_03_L1) is found in the second clade and clusters together with all other EBH samples as well as a strain that was found in a pet rabbit in Hesse (Central Germany). The latter is identical to a strain obtained from a EBH from Lower Saxony, approximately 140 km from the pet rabbit sampling location. Within both main clusters, a number of statistically supported subclades of geographically related samples, e.g., from the Czech Republic or Northern Germany, were found.

In our analysis, we identified positively selected sites (codons) in each of the target genes including *tp0488* (*n* = 29) and *tp0548* (*n* = 54) (Table S1). Those sites were removed from the alignments, and only nonpositively selected parsimony-informative sites as well as singletons were used for network constructions. The minimum spanning network that resulted from nonpositively selected single nucleotide variants within the *tp0548* locus (Fig. S1) does not change the overall topology of the maximum-likelihood tree shown in Fig. 2 and equally lacks the overall geographic clustering of the samples. Maximum-likelihood trees for individual loci can be found in the Supplemental Material (Fig. S2 and S3).

### Genetic diversity within *tp0548*

**Fig 3:**
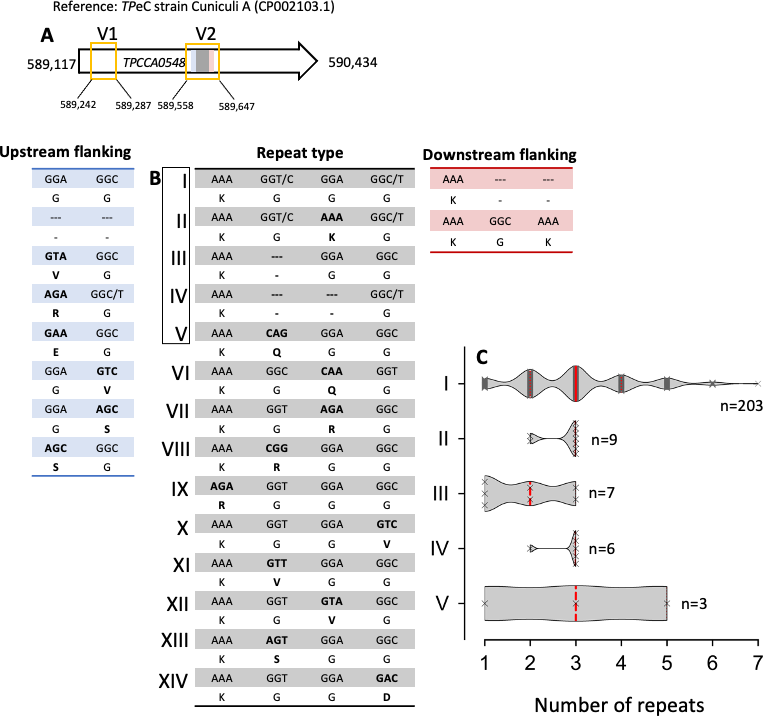
Illustration of the *tp0548* gene variable regions and repeat types. (A) Overview of the two identified variable regions (V1–2) at a minimum variant frequency of 0.25. The colors in V2 are coding for the flanking regions of the repeats upstream (blue) and downstream (red). The gray area indicates the location of the repeats. (B) Sequence information of the identified repeat types I-XIV. Changes from type I repeat are highlighted in bold. (C) Truncated violine plot of the frequency distribution of the five most abundant repeat types identified in our set of sequences. In red, the bold dashed lines indicate the median; the smaller dotted lines represent the quartiles. Individual data are superimposed with a gray cross. The total number of strains with the respective repeat type is shown on the right. Only repeat types occurring greater than three and which contain only a single repeat type are shown. Figure S4 provides further information on other repeat patterns. Violin plots were created in GraphPad PRISM 9.3.1.

While the *tp0488* gene in lagomorph infecting *TPe*C/L strains shows no defined sequence variability site at a chosen minimum variant frequency of 0.25, the *tp0548* gene in our analyzed samples had two hypervariable regions (V1–2). These regions range from 589,242 to 589,287 (V1) and 589,558–589,647 (V2) on the *TP*eC strain Cuniculi A reference genome (GenBank CP002103.1; Fig. 3A) and were characterized by an aggregation of polymorphic sites, deletions, and repeat patterns. Briefly, V1 is characterized through indels and a dominating arginine, serine, and glycine-coding composition. The V2 region is longer and includes various types of repetitions that are illustrated in Fig. 3. Most strains (*n* = 203/287) present with type I repetitions coding for a KGGG amino acid motif. The median number of repetitions of this dominating type I repeat is three with a range of one to seven (Fig. 3C). Besides the 228 strains that showed only one repeat type, 56 strains presented with a mosaic of two or three different repeat types, and three samples had no repeat sequence at all (Fig. S4).

### Host genus confirmation and hare population genetics

We tested a total of *n* = 152/565 MLST-positive lagomorphs—those that were sampled from third parties (e.g., hunters)—to validate the correct host genus assignment using amplification of the two nuclear loci IGHGCH2 and IGHG hinge regions. Briefly, from one sample, we retrieved only the IGHGCH2 target sequence, and for 13 samples, we were left with qualitative useful sequences from the IGHG hinge region only. For all others, we obtained sequence data for both loci. The BLAST search in all resulting sequences (Table S2) resulted in a query coverage of 96.94 ± 0.99% (mean ± SD) and 99.95 ± 0.09% (mean ± SD) identical sites to its closest related sequence in GenBank. Subsequently, the organism outcome of the respective BLAST result was compared to the host genus that was assigned by the sample provider. We could confirm the originally declared host genus (*Lepus* versus *Oryctolagus*) for all but one specimen. The latter (62BYMX121119) was originally labeled as of European rabbit origin but generated a sequence (joint IGHGCH2 and IGHG hinge gene target region) that is identical to the immunoglobulin gamma heavy chain constant region of EBH (query coverage of 96.94% and 100% identical sites to the closest related sequence in GenBank KJ807320).

We used the 95 randomly selected EBHs equally distributed across all sampling sites (Table S2) to further characterize the host population. A phylogenetic tree was constructed from the generated mt-genome sequence data in combination with published data from GenBank using the maximum-likelihood algorithm and Bayesian inference (Fig. S5). Here, we obtained three major clades, referring to populations in Turkey/Cyprus, Greece, and central/western Europe. Subsequently, the corresponding *TP*eL/C strains were assigned to the respective mt-genome sequence with no or only little geographical structuring.

## DISCUSSION

We show further evidence for a high prevalence of *TP*eC/L infection in wild lagomorphs in Europe based on DNA isolated from swab and tissue material. This complements our previous serological and qPCR findings that already suggested a widespread infection in European lagomorphs (10 – 12). Moreover, we confirm infection in three previously reported host species (EBH, mountain hare, and rabbits) and a newly identified host, the Corsican hare. Unfortunately, in this study, we cannot make a statement about actual prevalence rates, which is particularly the case for the two Corsican hare samples that are included in our sample set. Also, the high number of animals that had a PCR product of correct size visible on agarose gel and low sequence quality or high background noise due to superimposed sequences warrants further investigation into multi-strain infection and the effect of DNA degradation in dead-found animals (Table 1). It is, therefore, realistic to assume a higher prevalence than the one calculated based on high-quality sequence data. We believe that our previous publications will be helpful in this regard since they show infection based on serology and qPCR with a EBH prevalence rate of up to 46.5% (10 – 12).

Reports about hybridization in hare species (32 – 35) highlight feasible transmission pathways for the interspecies spread of *Treponema*. In this context, the demonstrated infection of a pet rabbit and a EBH from Central Germany with an identical strain (01GIF22900120 and 02DNTWF1100120, Fig. 1) is more difficult to explain because a direct interaction of the pet rabbit and wild hares can be excluded. Since the information content of MLST systems is limited, whole genome sequencing of the two strains is needed to prove whether the two *TP*eC/L isolates are truly identical. To our knowledge, sexual interactions of rabbits and hares—even under natural conditions—have not been reported. In primate infection (including humans) with the related bacterium *TPE*, vector transmission through flies has been discussed (17, 36, 37). Unfortunately, in lagomorphs, vector transmission of *Tp*eC/L has not been investigated.

Our current data contribute additional support for the high prevalence of treponematosis in wild European lagomorphs and add novel insight into the genetic diversity of lagomorph-infecting *Treponema* from Italy, the Czech Republic, Germany, Sweden, the Netherlands, and the United Kingdom (Fig. 1). We used our established MLST system for nonhuman primate yaws infection (14) to equally characterize lagomorph-infecting strains. This was done under the assumption that the lagomorph-infecting *TP*eC/L and TP are closely related as shown on the basis of the single published whole genome of *TP*eC (7, 9). Although MLST systems are generally limited in the amount of genetic information, only two genes have been used in this study; the results presented in Fig. 2 demonstrate an unexpectedly greater diversity compared to what we have seen in nontreated naturally yaws-infected nonhuman primates in sub-Saharan Africa. In the case of lagomorph *tp0548* sequences, 242 variants—including differences in the length of repetitions—out of 295 obtained sequences were found, demonstrating the enormously high degree of genetic variability. Yet, most variable nucleotides in both loci, *tp0488* and *tp0548*, were found under positive selection, partly explaining the huge observed genetic diversity and the lack of geographical clustering. Interestingly, a human syphilis MLST system also uses partial analysis of the *tp0548* gene for molecular typing of clinical isolates. Until now, 77 different alleles of *tp0548* in *TP* have been identified from a total of 944 investigated clinical isolates (38), suggesting that similar evolutionary forces operate on the *tp0548* locus in human- and lagomorph-infecting *Treponema*. The higher observed genetic diversity of *tp0548* in lagomorphs could be explained by prolonged infection in hares than in humans who are treated with antibiotics. It is open to debate whether the higher genetic diversity of lagomorph-infecting *TP*eC/L mimics treponemal evolution in an untreated human population.

The strain diversity, geographic range of infection, and the involvement of multiple lagomorph species are all indicators of the endemic character of the disease in European lagomorphs. In contrast to nonhuman primates (14) and human infections (3) with the sister bacterium *T. pallidum*, our current data from lagomorphs showed only weak geographic clustering. This is unexpected, since the biology of hares—a mostly philopatric species that shows only limited dispersal activity (39)—would likely contribute to the long-term circulation of regional (dominant) strains in the different hare metapopulations. It is open to debate whether this is the result of the positive selection of variants in the gene targets that we used for molecular typing or an effect of the anthropogenic influence on the population through trans- or relocation (40 – 42). Until today, the management of overexploited EBH populations is based on annual restocking (43). In combination with the mt-genome data (Fig. S5), which indicate a panmictic EBH population, it is most likely that EBH dispersal and, associated with this, the dispersal of *TP*eL strains are dominated by human influence.

In *TP*eC/L, the *tp0548* locus shows not only a higher number of nucleotide variants compared to nonhuman primate and human infection with *TPE* and *TPA*, respectively, but also various types of short repeat units that have not been described in primate treponematoses (Fig. 3). While the functional aspect of these *tp0548* short repeat units remains subject to ongoing investigations, it is likely that it enables the pathogen to better survive in its lagomorph host. From an evolutionary perspective, short, repeated nucleotide sequences in bacteria are frequently associated with higher replication error rates caused by slipped-strand mispairing (44, 45). It seems obvious that a provoked slipped-strand mispairing in structurally nonessential parts of antigenic outer membrane proteins provides an advantage over spontaneous mutations in terms of immune escape.

Apparently, the actual selected loci*—tp0488* and in particular *tp0548*—are not well suited for the molecular typing of treponemes of lagomorph origin. This is reflected by the high number of haplotypes. Once a reasonable number of whole genome sequences of *TP*eC/L becomes available, an in-depth revision of the current typing system is necessary to include more decently variable loci that are suitable for the epidemiological monitoring of transmission chains. Moreover, whole genome sequencing of modern and ancient samples from a wide geographic range and from multiple lagomorph species, including those that are not yet investigated [broom hare (*Lepus castroviejoi*) in northern Spain, the cape hare (*Lepus capensis*) in Sardinia and Cyprus, and the Iberian hare (*L. granatensis*) on the Iberian Peninsula] will help backtrack the evolutionary path of the pathogen and its relationship to modern syphilis in humans.

We have demonstrated a high prevalence of *TP*eC/L using nucleic amplification assays and subsequent Sanger sequencing. These methods prevent us from making a final statement on the viability of the treponemes. Yet, in the authors’ view, the consistence of infection in lagomorphs sampled across Europe and the high copy numbers detected in samples of the internal genitalia of EBHs (11) make an active infection likely. In human syphilis, with the exception of latent syphilis, active infection is associated with clinical lesions (46), which were rarely seen in the clinically inspected lagomorphs, of which 20/532 (3.8%) had typical skin lesions. Unfortunately, many samples included in this study originated from collaborating hunting parties, which limited the clinical expectation of the integument, the oral cavity, and the genital tract during the sampling procedure. In these samples, it cannot be excluded that lesions have been overlooked, for example, when the ulcer was located in the urethra as it is described for human syphilis (47).

### Conclusion

In our current study, we show a high proportion of wild European lagomorphs infected with *TP*eC/L, based on the detection of the pathogen’s DNA in genital swabs and tissue materials (Table 1). Sequencing of the targeted gene loci revealed an unexpectedly high genetic diversity. The various types of repetitions in one of the two hypervariable regions at the *tp0548* locus have not been described in the sister bacterium *TPA*, the causative agent of human syphilis. This warrants further research on the functional aspects of repetitive units in the genome of *TP*eC/L. A revision of the MLST system is recommended once a substantial number of lagomorph-infecting treponemes have been whole genome-sequenced.

## Data Availability

*Treponema paraluisleporidarum* haplotype sequences obtained from Sanger sequencing are available under GenBank accession numbers OM939693-OM939724 (*tp0488*) and OM990854-OM991094 (*tp0548*). Likewise, the mt-genome sequences can be found using the GenBank accession numbers OM993354-OM991094. Immunoglobulin heavy chain gene sequence data are available under the GenBank accession numbers ON089362-ON089577. Data are summarized in Table S1 (TPeC/L related data) and S2 (mt-genome data).

## SUPPLEMENTAL MATERIAL

Supplemental information spectrum.01774-23-s0001.pdf Figures S1 to S5.

Table S1 spectrum.01774-23-s0002.pdf Metadata and GenBank accession numbers for the samples included in this study.

Table S2 spectrum.01774-23-s0003.pdf GenBank accession numbers for the mt-genomes.

ASM does not own the copyrights to Supplemental Material that may be linked to, or accessed through, an article. The authors have granted ASM a non-exclusive, world-wide license to publish the Supplemental Material files. Please contact the corresponding author directly for reuse.
