# Supplementary material for: High prevalence and genetic diversity of Treponema paraluisleporidarum isolates in European lagomorphs
Source: Microbiol Spectr. 2023 Dec 14;12(1):e01774-23. doi: 10.1128/spectrum.01774-23 (PMC10783078; doi:10.1128/spectrum.01774-23)
Supplement: Supplemental information — Figures S1 to S5. [file spectrum.01774-23-s0001.pdf]

Technical Appendix

High prevalence and genetic diversity of *Treponema paraluisleporidarum* isolates in European lagomorphs

Running title: Prevalence and genetic diversity of *Treponema paraluisleporidarum*

Authors: Sascha Knauf,[a,b,c,*,§] Linda Hisgen,[a,b]* Erik O. Ågren,[d] Alexander M. Barlow,[e] Marcus Faehndrich,[f] Ulrich Voigt,[f] Luisa Fischer,[g] Linda Grillová,[h] Luisa K. Hallmaier-Wacker,[b] Marja J. L. Kik,[i] Jana C. Klink,[f] Jitka Křenová,[h] Antonio Lavazza,[j] Simone Lüert,[a,b] Markéta Nováková,[h] Darina Čejková,[k] Carlo Pacioni,[l,m] Tiziana Trogu,[k] David Šmajs,[h] Christian Roos[n,o]

[a]Institute of International Animal Health/One Health, Friedrich-Loeffler-Institut, Federal Research Institute for Animal Health, Greifswald – Insel Riems, Germany

[b]Infection Biology Unit, Deutsches Primatenzentrum GmbH, Leibniz Institute for Primate Research, Göttingen, Germany

[c]Professorship for International Animal Health/One Health, Faculty of Veterinary Medicine, Justus Liebig University, Giessen, Germany

[d]Department of Pathology and Wildlife Diseases, National Veterinary Institute, Uppsala, Sweden

[e]Wildlife Network for Disease Surveillance, Bristol Veterinary School, Langford, Somerset, United Kingdom

[f]Institute for Terrestrial and Aquatic Wildlife Research, University of Veterinary Medicine Hanover- Foundation, Hanover, Germany

[g]Wildlife Research Institute, State Agency for Nature, Environment and Consumer Protection North Rhine-Westphalia, Bonn, Germany
[h]Department of Biology, Masaryk University, Brno, Czech Republic
[i]Pathology Division, Department of Biomedical Health Sciences, Veterinary Medicine, Utrecht University, Utrecht, The Netherlands
[j]Istituto Zooprofilattico Sperimentale della Lombardia e dell'Emilia Romagna, Brescia, Italy
[k]Department of Biomedical Engineering, Brno University of Technology, Brno, Czech Republic
[l]Arthur Rylah Institute for Environmental Research, Heidelberg, VIC, Australia
[m]Environmental and Conservation Sciences, Murdoch University, Murdoch, WA, Australia
[n]Primate Genetics Laboratory, Deutsches Primatenzentrum GmbH, Leibniz Institute for Primate Research, Göttingen, Germany
[o]Gene Bank of Primates, Deutsches Primatenzentrum GmbH, Leibniz Institute for Primate Research, Göttingen, Germany
*Contributed equally

[§]**Correspondence** should be addressed to Prof Sascha Knauf, PhD habil., Institute of International Animal Health/One Health, Friedrich-Loeffler-Institut, Federal Institute for Animal Health, Südufer 10, 17493 Greifswald – Insel Riems, Germany, sascha.knauf@fli.de

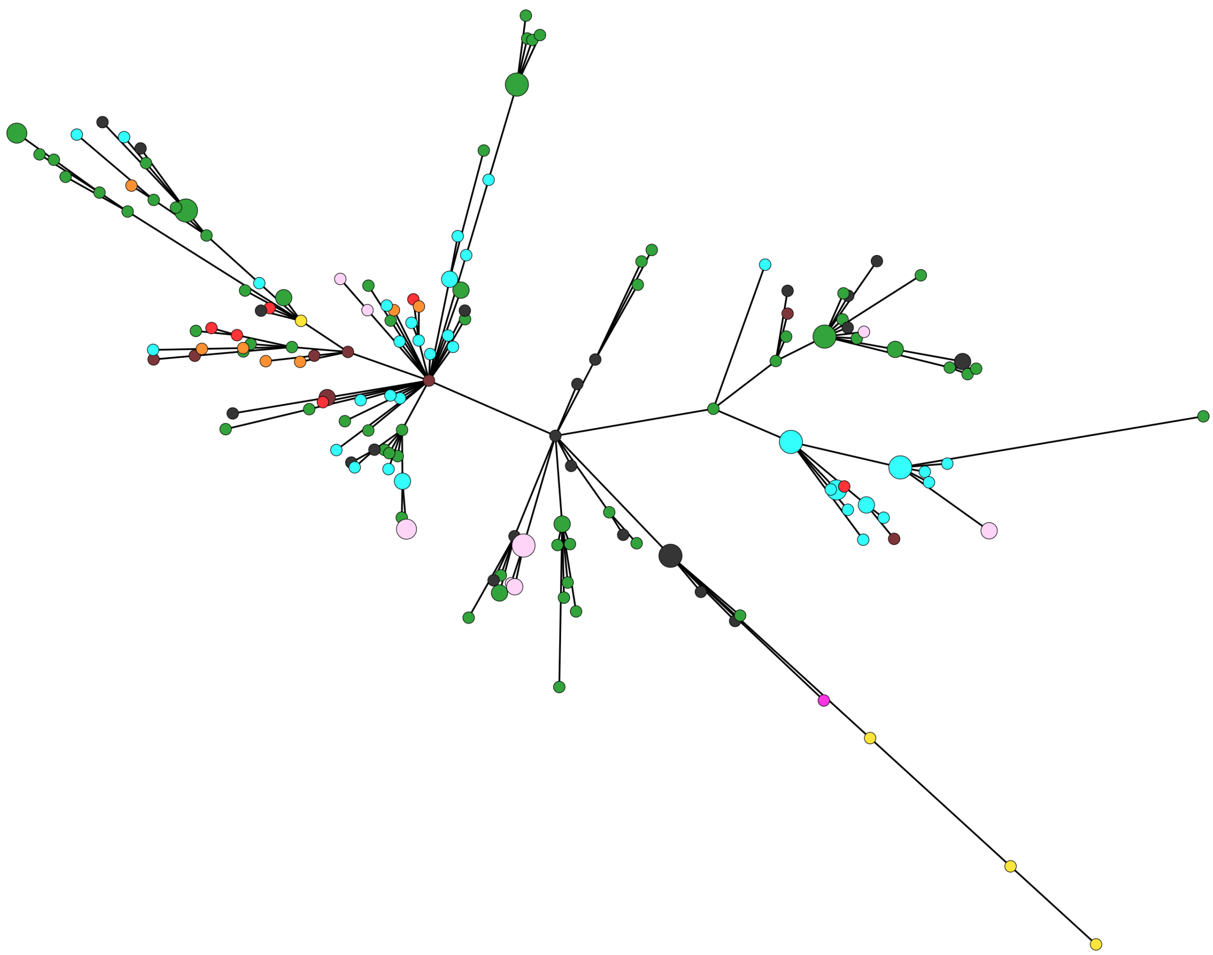

Figure S1. Minimum spanning network constructed using GrapeTree (Zhou et al., 2018) based on the concatenated sequences of the *tp0488* and *tp0548* genes. Positively selected sites have been excluded. The country origin is displayed by the colour of the nodes: magenta = USA (n=1, GenBank CP002103.1), yellow = Sweden (n = 4), cyan = Northern Germany (n = 45), black = Southern Germany (n=26), green = Central and Western Germany (n = 81), orange = The Netherlands (n=7), wine-red = Italy (n = 9), pink = Czech Republic (n = 15) and red = United Kingdom (6). No clear clustering can be seen based on the geographic origin.

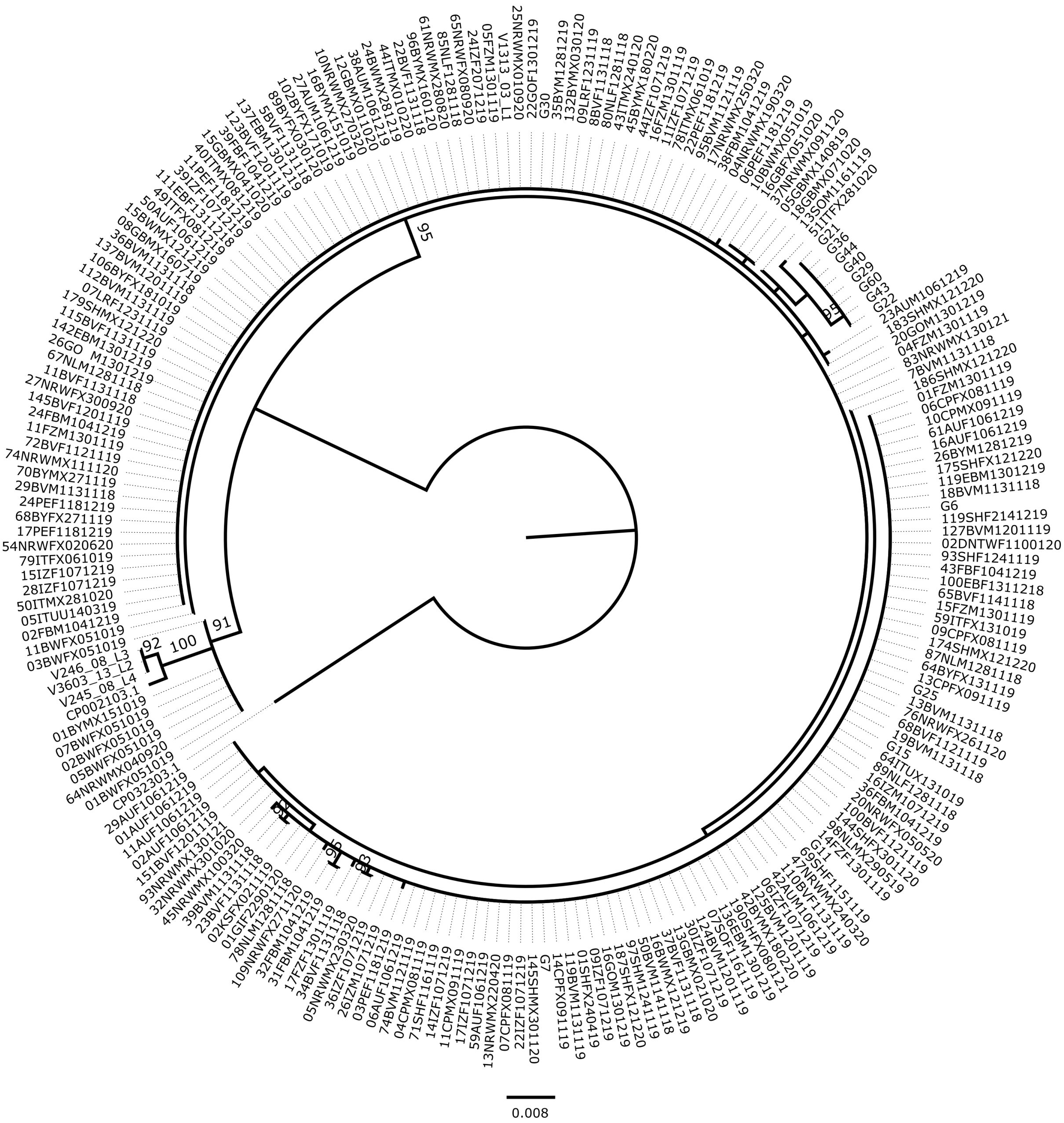

Figure S2. Maximum-likelihood tree based on the *tp0488* gene. The tree was constructed using IQ-TREE (Minh et al., 2020) with the best-fit model (TN+F+G4) based on the Bayesian Information Criterion and 1,000 bootstrap replicates. We included 212 sequences, containing 29 parsimony-informative sites and 39 singletons. Only bootstrap values > 90% are shown. Scale bar represents substitutions per nucleotide site. Details about the samples can be found in Table S1. The tree was rooted using *T. pallidum* subsp. *endemicum* strain Iraq B (GenBank CP032303.1).

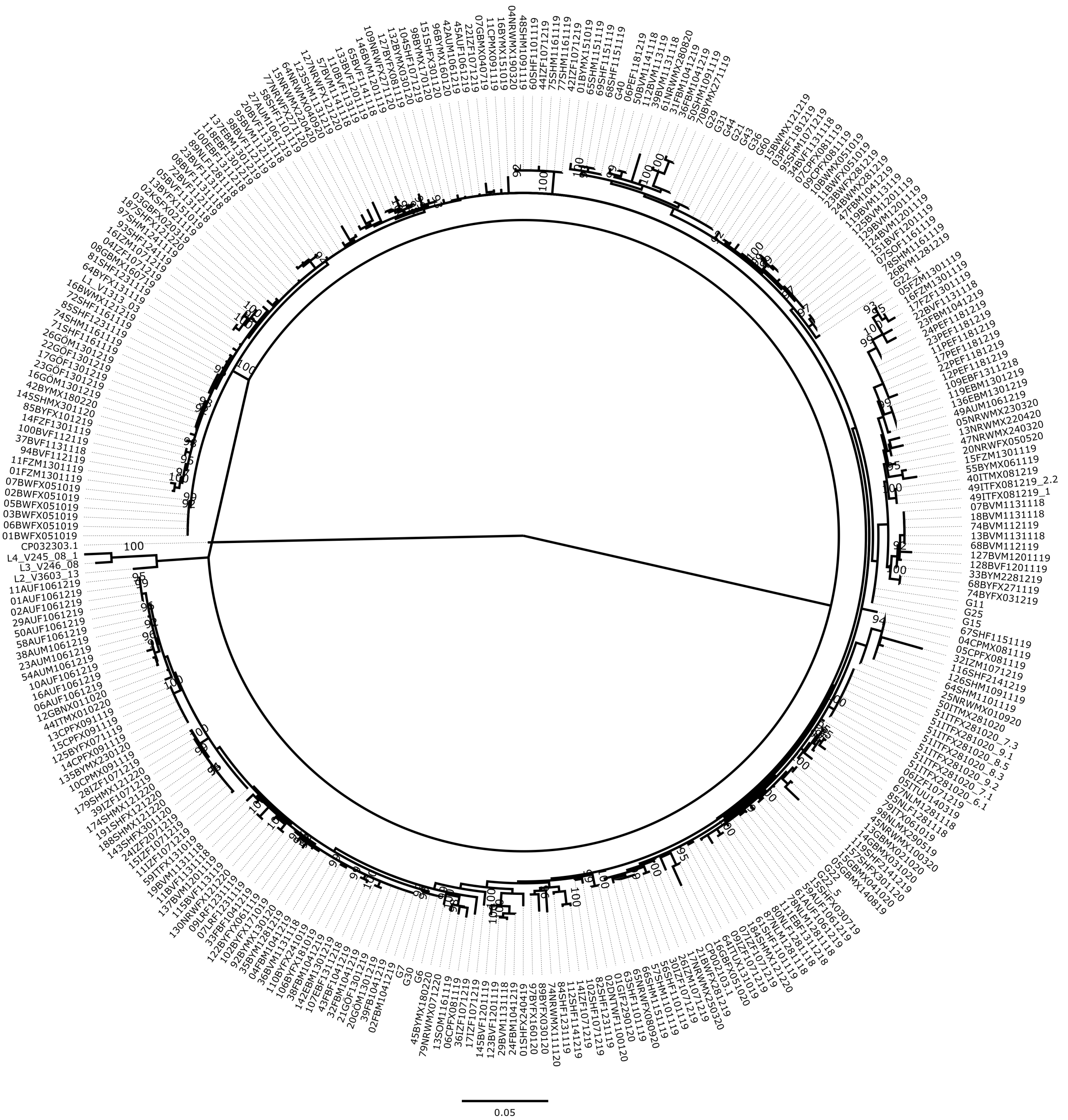

Figure S3. Maximum-likelihood tree based on the *tp0548* gene. The tree was constructed using IQ-TREE (Minh et al., 2020) with the best-fit model (TIMe+R3) based on the Bayesian Information Criterion and 1,000 bootstrap replicates. We included 296 sequences, containing 149 parsimony-informative sites and 95 singletons. Bootstrap values > 90% are shown. Scale bar represents substitutions per nucleotide site. Details about the samples can be found in Table S1. The tree was rooted using *T. pallidum* subsp. *endemicum* strain Iraq B (GenBank CP032303.1).

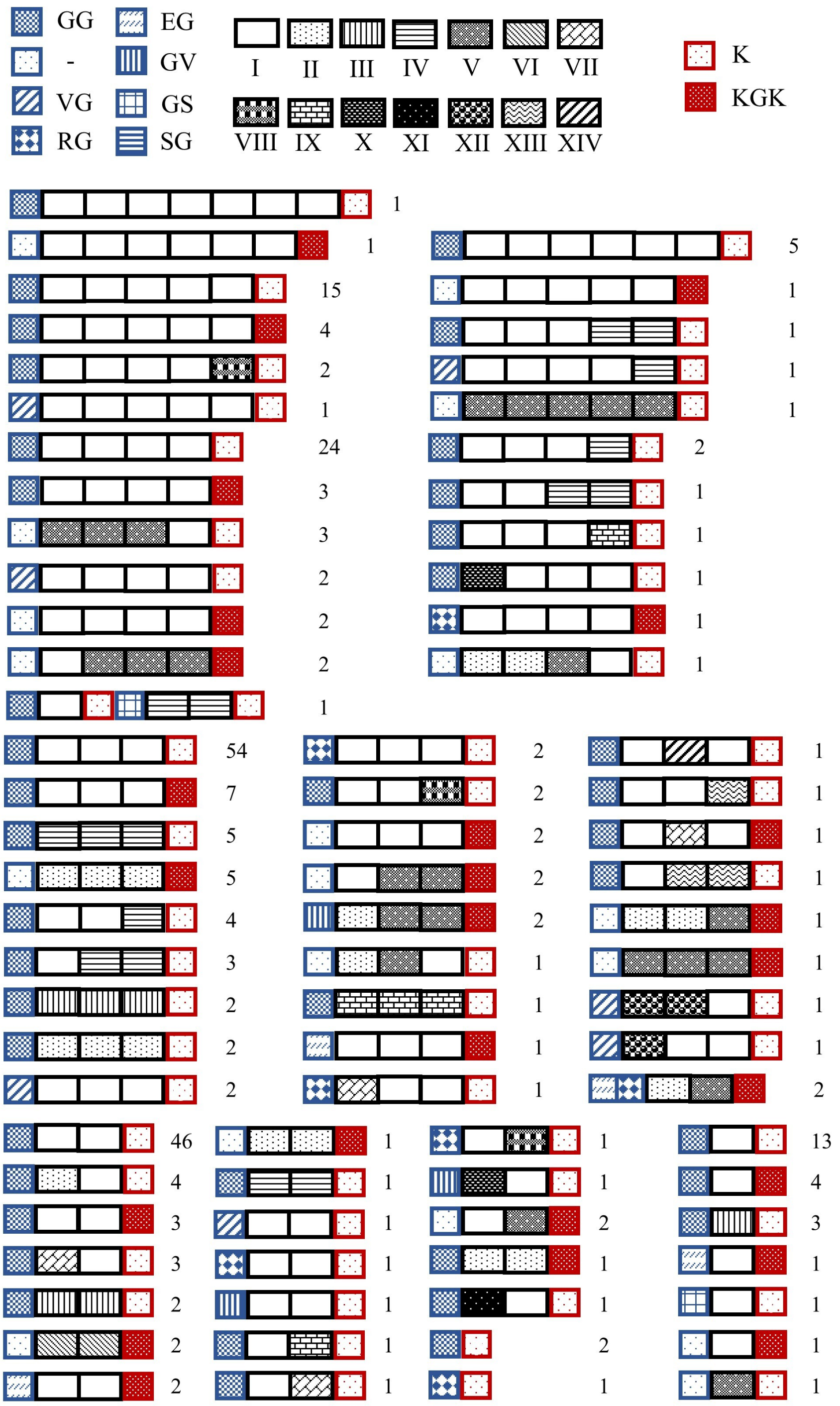

Figure S4. Overview of the haplotypes of the *tp0548*-repeat region and their overall frequency in our dataset. The legend provides details about the amino acid coding of the respective flanking region upstream (blue) and downstream (red) and the repeat type as described in Figure 2. The numbers indicate the frequency of the respective repeat haplotype in our dataset.

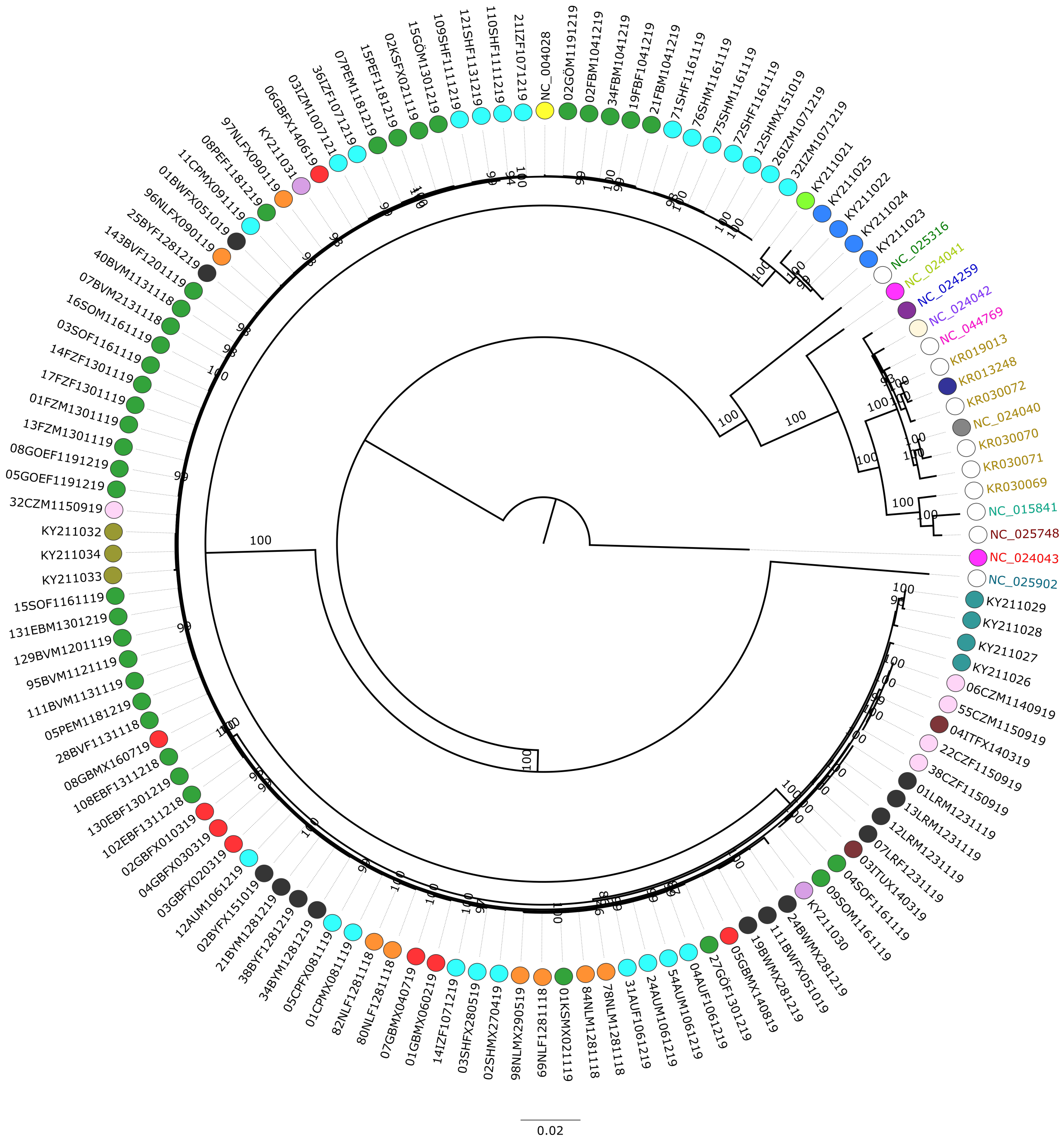

Figure S5. Maximum-likelihood tree based on mt-genomes of 126 hares. The 96 new sequences generated in this study are displayed with their sequence ID. Corresponding GenBank accession numbers can be found in Table S1. The tree was constructed with IQ-TREE with the best-fit model (GTR+F+I+G4) based on the Bayesian Information Criterion and 1,000 bootstrap replicates. We included a total of 126 sequences, containing 2,225 parsimony-informative sites and 1,615 singletons. Only bootstrap values > 90% are shown. Scale bar represents substitutions per nucleotide site. The tree was rooted using *Lepus*

americanus (NC_024043). The circle colour indicates the geographic origin of the sample: yellow = Sweden, cyan = Northern Germany, black = Southern Germany, green = Central and Western Germany, apple green = Turkey, blue = Cyprus, magenta = USA, dark lilac = Korea, orange = The Netherlands, wine-red = Italy, light pink = Czech Republic, olive = Poland, lilac = Germany unknown, white = unknown, beige = Spain, dark blue = China, dark grey = Finland, turquoise = Greece, and red = United Kingdom. Text colour indicates the host species: black = *Lepus europaeus*, green = *Lepus sinensis*, apple green = *Lepus townsendii*, blue = *Lepus coreanus*, lilac = *Lepus granatensis*, pink = *Lepus arcticus*, dark yellow = *Lepus timidus*, turquoise = *Lepus capensis*, wine-red = *Lepus tolai*, red = *Lepus americanus* and marine blue = *Lepus hainanus*. Scale bar represents substitutions per nucleotide site. Mt-genomes do not correspond to animals with treponemal infections but have been randomly selected for each region.

Table S1. Metadata and GenBank accession numbers for the samples included into this study.

Table S2. GenBank accession numbers for the mt-genomes.
