# Supplementary material for: High prevalence and genetic diversity of Treponema paraluisleporidarum isolates in European lagomorphs
Source: Microbiol Spectr. 2023 Dec 14;12(1):e01774-23. doi: 10.1128/spectrum.01774-23 (PMC10783078; doi:10.1128/spectrum.01774-23)
Supplement: Table S1 — Metadata and GenBank accession numbers for the samples included in this study. [file spectrum.01774-23-s0002.pdf]

										tp0488 PCR				*tp0548* PCR				*tp0548* repeat region (v2)							
Sample ID	Country Code	Colour Code	Clinical Infection Status	Location	Latitude	Longitude	GPS comment	Host Species	Host species confirmation: Genebank Accession number (aligned sequences)	Sequence incl. in Alignment	PCR inconclusive (see Table 1	No Amplification	Genebank Accession Number (Reference Genotypes are Shown in Bold)	Sequence incl. in Alignment	PCR inconclusive (see Table 1	No Amplification	Genebank Accession Number (Reference Genotypes are Shown in Bold)	Upstream Flanking (nt)	Upstream Flanking (aa)	Repeat Sequence (nt)	Repeat Sequence (aa)	Repeat Type	No. of Repetitions	Downstream Flanking (nt)	Downstream Flanking (aa)
CP002103.1	USA		1	unknown	unknown	unknown	NA	*Oryctolagus cuniculus f. domestica*	NA	NA	NA	NA	AEH40426.1	NA	NA	NA	**AEH40487.1**	GGAGGC	GG	AAAGGTGGAGGC	KGGG	I	5	AAA	K
V1313_03_L1	SWE		1	Sweden	59.81885	17.65728	estimated	*Lepus timidus*	**ON089574**	1	0	0	OM939693	1	0	0	**OM991091**	GGAGGC	GG	AAAGGTGGAGGC	KGGG	I	4	AAA	K
V3603_13_L2	SWE		1	Sweden	61.55000	16.98333	estimated	*Lepus timidus*	**ON089575**	1	0	0	OM939699	1	0	0	**OM991094**	GGAGGC	GG	NA	NA	NA	NA	AAA	K
V246_08_L3	SWE		1	Sweden	59.81885	17.65728	estimated	*Lepus timidus*	**ON089576**	1	0	0	OM939699	1	0	0	**OM991093**	AGAGGC	RG	NA	NA	NA	NA	AAA	K
V245_08_1_L4	SWE		1	Sweden	59.81885	17.65728	estimated	*Lepus timidus*	**ON089577**	1	0	0	**OM939699**	1	0	0	**OM991092**	NA	NA	AAAGGC	KG	V	1	AAA	K
01ITUX140319	IT		unknown	Castellucchio	45.149517	10.648699	estimated	*Lepus europaeus*	NA	0	0	1	NA	0	0	1	NA	NA	NA	NA	NA	NA	NA	NA	NA
02ITMX140319	IT		unknown	Vigarano Mainarda	44.840930	11.493340	estimated	*Lepus europaeus*	**ON089369**	0	0	1	NA	0	0	1	NA	NA	NA	NA	NA	NA	NA	NA	NA
03ITUX140319	IT		unknown	Roverbella	45.260651	10.808983	estimated	*Lepus europaeus*	NA	0	0	1	NA	0	0	1	NA	NA	NA	NA	NA	NA	NA	NA	NA
04ITFX140319	IT		unknown	Zocca	44.366397	11.000216	estimated	*Lepus europaeus*	**ON089376**	0	1	0	NA	0	1	0	NA	NA	NA	NA	NA	NA	NA	NA	NA
05ITXX140319	IT		unknown	Rivergaro	44.913724	9.607967	estimated	*Lepus europaeus*	**ON089380**	1	0	0	OM939693	1	0	0	**OM990868**	GGAGGC	GG	AAAGGTGGAGGC	KGGG	I	5	AAA	K
06ITMX060120	IT		unknown	Mesola	44.92217	12.26018	estimated	*Lepus europaeus*	NA	0	0	1	NA	0	0	1	NA	NA	NA	NA	NA	NA	NA	NA	NA
07ITFX060120	IT		unknown	Mesola	44.92217	12.26018	estimated	*Lepus europaeus*	NA	0	0	1	NA	0	0	1	NA	NA	NA	NA	NA	NA	NA	NA	NA
08ITMX060120	IT		unknown	Mesola	44.92217	12.26018	estimated	*Lepus europaeus*	NA	0	0	1	NA	0	0	1	NA	NA	NA	NA	NA	NA	NA	NA	NA
09ITMX060120	IT		unknown	Mesola	44.92217	12.26018	estimated	*Lepus europaeus*	NA	0	0	1	NA	0	0	1	NA	NA	NA	NA	NA	NA	NA	NA	NA
10ITFX060120	IT		unknown	Mesola	44.92217	12.26018	estimated	*Lepus europaeus*	NA	0	0	1	NA	0	0	1	NA	NA	NA	NA	NA	NA	NA	NA	NA
11ITFX060120	IT		unknown	Mesola	44.92217	12.26018	estimated	*Lepus europaeus*	NA	0	0	1	NA	0	0	1	NA	NA	NA	NA	NA	NA	NA	NA	NA
12ITFX060120	IT		unknown	Mesola	44.92217	12.26018	estimated	*Lepus europaeus*	NA	0	0	1	NA	0	0	1	NA	NA	NA	NA	NA	NA	NA	NA	NA
13ITFX171219	IT		unknown	Imola	44.334750	11.729612	estimated	*Lepus europaeus*	NA	0	0	1	NA	0	0	1	NA	NA	NA	NA	NA	NA	NA	NA	NA
14ITFX171219	IT		unknown	Imola	44.334750	11.729612	estimated	*Lepus europaeus*	NA	0	0	1	NA	0	0	1	NA	NA	NA	NA	NA	NA	NA	NA	NA
15ITMX171219	IT		unknown	Imola	44.334750	11.729612	estimated	*Lepus europaeus*	NA	0	0	1	NA	0	0	1	NA	NA	NA	NA	NA	NA	NA	NA	NA
16ITMX171219	IT		unknown	Imola	44.334750	11.729612	estimated	*Lepus europaeus*	NA	0	0	1	NA	0	0	1	NA	NA	NA	NA	NA	NA	NA	NA	NA
17ITFX171219	IT		unknown	Imola	44.334750	11.729612	estimated	*Lepus europaeus*	NA	0	0	1	NA	0	0	1	NA	NA	NA	NA	NA	NA	NA	NA	NA
18ITMX171219	IT		unknown	Imola	44.334750	11.729612	estimated	*Lepus europaeus*	NA	0	0	1	NA	0	0	1	NA	NA	NA	NA	NA	NA	NA	NA	NA
19ITFX171219	IT		unknown	Imola	44.334750	11.729612	estimated	*Lepus europaeus*	NA	0	0	1	NA	0	0	1	NA	NA	NA	NA	NA	NA	NA	NA	NA
20ITMX171219	IT		unknown	Imola	44.334750	11.729612	estimated	*Lepus europaeus*	NA	0	0	1	NA	0	0	1	NA	NA	NA	NA	NA	NA	NA	NA	NA
21ITFX171219	IT		unknown	Imola	44.334750	11.729612	estimated	*Lepus europaeus*	**ON089431**	0	1	0	NA	0	1	0	NA	NA	NA	NA	NA	NA	NA	NA	NA
22ITFX171219	IT		unknown	Imola	44.334750	11.729612	estimated	*Lepus europaeus*	NA	0	0	1	NA	0	0	1	NA	NA	NA	NA	NA	NA	NA	NA	NA
23ITFX151219	IT		unknown	Comacchio	44.700166	12.177793	estimated	*Lepus europaeus*	NA	0	0	1	NA	0	0	1	NA	NA	NA	NA	NA	NA	NA	NA	NA
24ITMX151219	IT		unknown	Comacchio	44.700166	12.177793	estimated	*Lepus europaeus*	NA	0	0	1	NA	0	0	1	NA	NA	NA	NA	NA	NA	NA	NA	NA
25ITFX151219	IT		unknown	Comacchio	44.700166	12.177793	estimated	*Lepus europaeus*	NA	0	0	1	NA	0	0	1	NA	NA	NA	NA	NA	NA	NA	NA	NA
26ITFX151219	IT		unknown	Comacchio	44.700166	12.177793	estimated	*Lepus europaeus*	NA	0	0	1	NA	0	0	1	NA	NA	NA	NA	NA	NA	NA	NA	NA
27ITMX151219	IT		unknown	Comacchio	44.700166	12.177793	estimated	*Lepus europaeus*	NA	0	0	1	NA	0	0	1	NA	NA	NA	NA	NA	NA	NA	NA	NA
28ITMX151219	IT		unknown	Comacchio	44.700166	12.177793	estimated	*Lepus europaeus*	NA	0	0	1	NA	0	0	1	NA	NA	NA	NA	NA	NA	NA	NA	NA
29ITMX151219	IT		unknown	Comacchio	44.700166	12.177793	estimated	*Lepus europaeus*	NA	0	0	1	NA	0	0	1	NA	NA	NA	NA	NA	NA	NA	NA	NA
30ITMX151219	IT		unknown	Comacchio	44.700166	12.177793	estimated	*Lepus europaeus*	NA	0	0	1	NA	0	0	1	NA	NA	NA	NA	NA	NA	NA	NA	NA
31ITFX151219	IT		unknown	Comacchio	44.700166	12.177793	estimated	*Lepus europaeus*	NA	0	0	1	NA	0	0	1	NA	NA	NA	NA	NA	NA	NA	NA	NA
32ITMX151219	IT		unknown	Comacchio	44.700166	12.177793	estimated	*Lepus europaeus*	NA	0	0	1	NA	0	0	1	NA	NA	NA	NA	NA	NA	NA	NA	NA
33ITFX081219	IT		unknown	Lagosanto	44.731336	12.199408	estimated	*Lepus europaeus*	NA	0	0	1	NA	0	0	1	NA	NA	NA	NA	NA	NA	NA	NA	NA
34ITFX081219	IT		unknown	Lagosanto	44.731336	12.199408	estimated	*Lepus europaeus*	NA	0	0	1	NA	0	0	1	NA	NA	NA	NA	NA	NA	NA	NA	NA
35ITFX081219	IT		unknown	Lagosanto	44.731336	12.199408	estimated	*Lepus europaeus*	NA	0	0	1	NA	0	0	1	NA	NA	NA	NA	NA	NA	NA	NA	NA
36ITMX081219	IT		unknown	Lagosanto	44.731336	12.199408	estimated	*Lepus europaeus*	NA	0	0	1	NA	0	0	1	NA	NA	NA	NA	NA	NA	NA	NA	NA
37ITFX081219	IT		unknown	Lagosanto	44.731336	12.199408	estimated	*Lepus europaeus*	NA	0	0	1	NA	0	0	1	NA	NA	NA	NA	NA	NA	NA	NA	NA
38ITMX081219	IT		unknown	Lagosanto	44.731336	12.199408	estimated	*Lepus europaeus*	NA	0	0	1	NA	0	0	1	NA	NA	NA	NA	NA	NA	NA	NA	NA
39ITFX081219	IT		unknown	Lagosanto	44.731336	12.199408	estimated	*Lepus europaeus*	NA	0	0	1	NA	0	0	1	NA	NA	NA	NA	NA	NA	NA	NA	NA
40ITMX081219	IT		unknown	Lagosanto	44.731336	12.199408	estimated	*Lepus europaeus*	**ON089446**	1	0	0	OM939693	1	0	0	**OM990955**	GAAGGCAGAGGTAAAGGCAAAGGT	EGRGKGKG	AAAGGC	KG	NA	2	AAA	K
41ITFX081219	IT		unknown	Lagosanto	44.731336	12.199408	estimated	*Lepus europaeus*	NA	0	0	1	NA	0	0	1	NA	NA	NA	NA	NA	NA	NA	NA	NA
42ITMX081219	IT		unknown	Lagosanto	44.731336	12.199408	estimated	*Lepus europaeus*	NA	0	0	1	NA	0	0	1	NA	NA	NA	NA	NA	NA	NA	NA	NA
43ITMX240120	IT		unknown	Follonica	42.935136	10.747320	estimated	*Lepus europaeus*	**ON089450**	1	0	0	OM939693	0	1	0	NA	NA	NA	NA	NA	NA	NA	NA	NA
44ITMX010220	IT		unknown	Follonica	42.935136	10.747320	estimated	*Lepus europaeus*	**ON089451**	1	0	0	OM939693	1	0	0	**OM990959**	GGAGGC	GG	AAAGGTGGAGGC	KGGG	NA	2	AAACGGGAAGGCAAA	KRGGK
45ITMX010220	IT		unknown	Follonica	42.935136	10.747320	estimated	*Lepus europaeus*	NA	0	0	1	NA	0	0	1	NA	NA	NA	NA	NA	NA	NA	NA	NA
46ITMX010220	IT		unknown	Follonica	42.935136	10.747320	estimated	*Lepus europaeus*	NA	0	0	1	NA	0	0	1	NA	NA	NA	NA	NA	NA	NA	NA	NA
47ITMX010220	IT		unknown	Follonica	42.935136	10.747320	estimated	*Lepus europaeus*	NA	0	0	1	NA	0	0	1	NA	NA	NA	NA	NA	NA	NA	NA	NA
48ITFX010220	IT		unknown	Follonica	42.935136	10.747320	estimated	*Lepus europaeus*	NA	0	0	1	NA	0	0	1	NA	NA	NA	NA	NA	NA	NA	NA	NA
49ITFX081219_1	IT		unknown	Lagosanto	44.731336	12.199408	estimated	*Lepus europaeus*	**ON089457**	1	0	0	OM939693	1	0	0	OM990955	GAAGGCAGAGGTAAAGGCAAAGGT	EGRGKGKG	AAAGGC	KG	NA	2	AAA	K
49ITFX081219_2.2	IT		unknown	Lagosanto	44.731336	12.199408	estimated	*Lepus europaeus*	NA	NA	NA	NA	NA	NA	NA	NA	OM990955	GAAGGCAGAGGTAAAGGCAAAGGT	EGRGKGKG	AAAGGC	KG	NA	2	AAA	K
50ITMX281020	IT		1	Agrigento	37.403080	13.747163	estimated	*Lepus corsicanus*	**ON089459**	1	0	0	OM939693	1	0	0	**OM990970**	GGAGGC	GG	AAAGGTGGAGGC	KGGG	I	2	AAA	K
51ITFX281020_6.1	IT		1	Messina	37.967569	14.287152	estimated	*Lepus corsicanus*	**ON089461**	1	0	0	**OM939707**	1	0	0	OM990970	GGAGGC	GG	AAAGGTGGAGGC	KGGG	I	2	AAA	K
51ITFX281020_7.1	IT		1	Messina	37.967569	14.287152	estimated	*Lepus corsicanus*	NA	NA	NA	NA	NA	NA	NA	NA	OM990970	GGAGGC	GG	AAAGGTGGAGGC	KGGG	I	2	AAA	K
51ITFX281020_7.3	IT		1	Messina	37.967569	14.287152	estimated	*Lepus corsicanus*	NA	NA	NA	NA	NA	NA	NA	NA	OM990970	GGAGGC	GG	AAAGGTGGAGGC	KGGG	I	2	AAA	K
51ITFX281020_8.3	IT		1	Messina	37.967569	14.287152	estimated	*Lepus corsicanus*	NA	NA	NA	NA	NA	NA	NA	NA	OM990970	GGAGGC	GG	AAAGGTGGAGGC	KGGG	I	2	AAA	K
51ITFX281020_8.5	IT		1	Messina	37.967569	14.287152	estimated	*Lepus corsicanus*	NA	NA	NA	NA	NA	NA	NA	NA	OM990970	GGAGGC	GG	AAAGGTGGAGGC	KGGG	I	2	AAA	K
51ITFX281020_9.1	IT		1	Messina	37.967569	14.287152	estimated	*Lepus corsicanus*	NA	NA	NA	NA	NA	NA	NA	NA	OM990970	GGAGGC	GG	AAAGGTGGAGGC	KGGG	I	2	AAA	K
51ITFX281020_9.2	IT		1	Messina	37.967569	14.287152	estimated	*Lepus corsicanus*	NA	NA	NA	NA	NA	NA	NA	NA	OM990970	GGAGGC	GG	AAAGGTGGAGGC	KGGG	I	2	AAA	K
52ITMX021019	IT		unknown	Garniano	45.692958	10.664057	estimated	*Lepus europaeus*	NA	0	0	1	NA	0	0	1	NA	NA	NA	NA	NA	NA	NA	NA	NA
53ITFX031019	IT		unknown	Bozzolo	45.107321	10.487834	estimated	*Lepus europaeus*	NA	0	0	1	NA	0	0	1	NA	NA	NA	NA	NA	NA	NA	NA	NA
54ITFX031019	IT		unknown	Bozzolo	45.107321	10.487834	estimated	*Lepus europaeus*	NA	0	0	1	NA	0	0	1	NA	NA	NA	NA	NA	NA	NA	NA	NA
55ITMX091019	IT		unknown	Desenzano	45.456439	10.551671	estimated	*Lepus europaeus*	NA	0	0	1	NA	0	0	1	NA	NA	NA	NA	NA	NA	NA	NA	NA
56ITMX121019	IT		unknown	Lonato	45.467642	10.487625	estimated	*Lepus europaeus*	NA	0	0	1	NA	0	0	1	NA	NA	NA	NA	NA	NA	NA	NA	NA
57ITFX121019	IT		unknown	Lonato	45.467642	10.487625	estimated	*Lepus europaeus*	NA	0	0	1	NA	0	0	1	NA	NA	NA	NA	NA	NA	NA	NA	NA
58ITFX131019	IT		unknown	Commessaggio	45.036474	10.549372	estimated	*Lepus europaeus*	NA	0	0	1	NA	0	0	1	NA	NA	NA	NA	NA	NA	NA	NA	NA
59ITFX131019	IT		unknown	Lonato	45.467642	10.487625	estimated	*Lepus europaeus*	**ON089469**	1	0	0	OM939694	1	0	0	**OM990979**	GGAGGC	GG	AAAGGTGGAGGC	KGGG	I	4	AAA	K
60ITFX131019	IT		unknown	Lonato	45.467642	10.487625	estimated	*Lepus europaeus*	NA	0	0	1	NA	0	0	1	NA	NA	NA	NA	NA	NA	NA	NA	NA
61ITFX131019	IT		unknown	Desenzano	45.456439	10.551671	estimated	*Lepus europaeus*	NA	0	0	1	NA	0	0	1	NA	NA	NA	NA	NA	NA	NA	NA	NA
62ITUX101019	IT		unknown	Desenzano	45.456439	10.551671	estimated	*Lepus europaeus*	NA	0	0	1	NA	0	0	1	NA	NA	NA	NA	NA	NA	NA	NA	NA
63ITUX131019	IT		unknown	Desenzano	45.456439	10.551671	estimated	*Lepus europaeus*	NA	0	0	1	NA	0	0	1	NA	NA	NA	NA	NA	NA	NA	NA	NA
64ITUX131019	IT		unknown	Desenzano	45.456439	10.551671	estimated	*Lepus europaeus*	**ON089476**	1	0	0	OM939694	1	0	0	**OM990985**	GGAGGC	GG	AAAGGTGGAGGC	KGGG	I	5	AAA	K
65ITUX001119	IT		unknown	Desenzano	45.456439	10.551671	estimated	*Lepus europaeus*	NA	0	0	1	NA	0	0	1	NA	NA	NA	NA	NA	NA	NA	NA	NA
66ITUX001119	IT		unknown	Desenzano	45.456439	10.551671	estimated	*Lepus europaeus*	NA	0	0	1	NA	0	0	1	NA	NA	NA	NA	NA	NA	NA	NA	NA
67ITUX001119	IT		unknown	Desenzano	45.456439	10.551671	estimated	*Lepus europaeus*	NA	0	0	1	NA	0	0	1	NA	NA	NA	NA	NA	NA	NA	NA	NA
68ITUX001119	IT		unknown	Desenzano	45.456439	10.551671	estimated	*Lepus europaeus*	NA	0	0	1	NA	0	0	1	NA	NA	NA	NA	NA	NA	NA	NA	NA
69ITUX001119	IT		unknown	Desenzano	45.456439	10.551671	estimated	*Lepus europaeus*	NA	0	0	1	NA	0	0	1	NA	NA	NA	NA	NA	NA	NA	NA	NA
70ITUX001119	IT		unknown	Desenzano	45.456439	10.551671	estimated	*Lepus europaeus*	NA	0	0	1	NA	0	0	1	NA	NA	NA	NA	NA	NA	NA	NA	NA
71ITUX001119	IT		unknown	Desenzano	45.456439	10.551671	estimated	*Lepus europaeus*	NA	0	0	1	NA	0	0	1	NA	NA	NA	NA	NA	NA	NA	NA	NA
72ITFX121019	IT		unknown	Pozzolengo	45.41229	10.62323	estimated	*Lepus europaeus*	NA	0	0	1	NA	0	0	1	NA	NA	NA	NA	NA	NA	NA	NA	NA
73ITMX131019	IT		unknown	Pozzolengo	45.41229	10.62323	estimated	*Lepus europaeus*	NA	0	0	1	NA	0	0	1	NA	NA	NA	NA	NA	NA	NA	NA	NA
74ITFX131019*	IT		unknown	Pozzolengo	45.41229	10.62323	estimated	*Lepus europaeus*	NA	0	0	1	NA	0	0	1	NA	NA	NA	NA	NA	NA	NA	NA	NA
75ITFX121019*	IT		unknown	Pozzolengo	45.41229	10.62323	estimated	*Lepus europaeus*	NA	0	0	1	NA	0	0	1	NA	NA	NA	NA	NA	NA	NA	NA	NA
76ITFX121019	IT		unknown	Pozzolengo	45.41229	10.62323	estimated	*Lepus europaeus*	NA	0	0	1	NA	0	0	1	NA	NA	NA	NA	NA	NA	NA	NA	NA
77ITFX061119	IT		unknown	Pozzolengo	45.41229	10.62323	estimated	*Lepus europaeus*	**ON089499**	0	1	0	NA	0	1	0	NA	NA	NA	NA	NA	NA	NA	NA	NA
78ITMX061019	IT		unknown	Pozzolengo	45.41229	10.62323	estimated	*Lepus europaeus*	**ON089502**	1	0	0	OM939693	0	1	0	NA	NA	NA	NA	NA	NA	NA	NA	NA

79ITFX061019	IT		unknown	Pozzolengo	45.41229	10.62323	estimated	*Lepus europaeus*	**ON089505**	1	0	0	OM939693	1	0	0	**OM991005**	GGAGGC	GG	AAAGGTGGAGGC	KGGG	I	3	AAA	K
80ITFX191019	IT		unknown	Pozzolengo	45.41229	10.62323	estimated	*Lepus europaeus*	NA	0	0	1	NA	0	0	1	NA	NA	NA	NA	NA	NA	NA	NA	NA
81ITMX231019	IT		unknown	Pozzolengo	45.41229	10.62323	estimated	*Lepus europaeus*	NA	0	0	1	NA	0	0	1	NA	NA	NA	NA	NA	NA	NA	NA	NA
67NLM1281118	NL		0	Wilp	52.21760	6.10586	accurate	*Lepus europaeus*	NA	1	0	0	OM939693	1	0	0	**OM990992**	AGAGGC	RG	AAAGGTGGAGGC	KGGG	I	3	AAA	K
68NLM1281118	NL		0	Wilp	52.21760	6.10586	accurate	*Lepus europaeus*	NA	0	1	0	NA	0	0	1	NA	NA	NA	NA	NA	NA	NA	NA	NA
69NLF1281118	NL		0	Wilp	52.21760	6.10586	accurate	*Lepus europaeus*	NA	0	1	0	NA	0	1	0	NA	NA	NA	NA	NA	NA	NA	NA	NA
70NLM1281118	NL		0	Wilp	52.21760	6.10586	accurate	*Lepus europaeus*	NA	0	0	1	NA	0	0	1	NA	NA	NA	NA	NA	NA	NA	NA	NA
71NLF1281118	NL		0	Wilp	52.21760	6.10586	accurate	*Lepus europaeus*	NA	0	0	1	NA	0	0	1	NA	NA	NA	NA	NA	NA	NA	NA	NA
72NLF1281118	NL		0	Wilp	52.21760	6.10586	accurate	*Lepus europaeus*	NA	0	0	1	NA	0	0	1	NA	NA	NA	NA	NA	NA	NA	NA	NA
73NLF1281118	NL		0	Wilp	52.21760	6.10586	accurate	*Lepus europaeus*	NA	0	1	0	NA	0	1	0	NA	NA	NA	NA	NA	NA	NA	NA	NA
74NLM1281118	NL		0	Wilp	52.21760	6.10586	accurate	*Lepus europaeus*	NA	0	0	1	NA	0	0	1	NA	NA	NA	NA	NA	NA	NA	NA	NA
75NLM1281118	NL		0	Wilp	52.21760	6.10586	accurate	*Lepus europaeus*	NA	0	1	0	NA	0	0	1	NA	NA	NA	NA	NA	NA	NA	NA	NA
76NLM1281118	NL		0	Wilp	52.21760	6.10586	accurate	*Lepus europaeus*	NA	0	1	0	NA	0	0	1	NA	NA	NA	NA	NA	NA	NA	NA	NA
77NLM1281118	NL		0	Wilp	52.21760	6.10586	accurate	*Lepus europaeus*	NA	0	1	0	NA	0	0	1	NA	NA	NA	NA	NA	NA	NA	NA	NA
78NLM1281118	NL		0	Wilp	52.21760	6.10586	accurate	*Lepus europaeus*	NA	1	0	0	**OM939718**	1	0	0	**OM991003**	GGAGGC	GG	AAAGGTGGAGGC	KGGG	I	2	AAA	K
79NLM1281118	NL		0	Wilp	52.21760	6.10586	accurate	*Lepus europaeus*	NA	0	1	0	NA	0	0	1	NA	NA	NA	NA	NA	NA	NA	NA	NA
80NLF1281118	NL		0	Wilp	52.21760	6.10586	accurate	*Lepus europaeus*	NA	1	0	0	OM939693	1	0	0	**OM991007**	GGAGGC	GG	AAAGGTGGAGGC	KGGG	NA	2	AAAGGAGGCAAA	KGGK
81NLM1281118	NL		0	Wilp	52.21760	6.10586	accurate	*Lepus europaeus*	NA	0	1	0	NA	0	1	0	NA	NA	NA	NA	NA	NA	NA	NA	NA
82NLF1281118	NL		0	Wilp	52.21760	6.10586	accurate	*Lepus europaeus*	NA	0	0	1	NA	0	0	1	NA	NA	NA	NA	NA	NA	NA	NA	NA
83NLF1281118	NL		0	Wilp	52.21760	6.10586	accurate	*Lepus europaeus*	NA	0	0	1	NA	0	0	1	NA	NA	NA	NA	NA	NA	NA	NA	NA
84NLM1281118	NL		0	Wilp	52.21760	6.10586	accurate	*Lepus europaeus*	NA	0	0	1	NA	0	0	1	NA	NA	NA	NA	NA	NA	NA	NA	NA
85NLF1281118	NL		0	Wilp	52.21760	6.10586	accurate	*Lepus europaeus*	NA	1	0	0	OM939693	1	0	0	**OM991012**	AGAGGC	RG	AAAGGTGGAGGC	KGGG	NA	1	AAACGGGGAGGCAAA	KRGGK
86NLF1281118	NL		0	Wilp	52.21760	6.10586	accurate	*Lepus europaeus*	NA	0	1	0	NA	0	1	0	NA	NA	NA	NA	NA	NA	NA	NA	NA
87NLM1281118	NL		0	Wilp	52.21760	6.10586	accurate	*Lepus europaeus*	NA	1	0	0	OM939694	1	0	0	OM991007	GGAGGC	GG	AAAGGTGGAGGC	KGGG	NA	2	AAAGGAGGCAAA	KGGK
88NLF1281118	NL		0	Wilp	52.21760	6.10586	accurate	*Lepus europaeus*	NA	0	0	1	NA	0	0	1	NA	NA	NA	NA	NA	NA	NA	NA	NA
89NLF1281118	NL		0	Wilp	52.21760	6.10586	accurate	*Lepus europaeus*	NA	1	0	0	OM939694	1	0	0	**OM991014**	GGAGGC	GG	AAAGGTGGAGGC	KGGG	I	3	AAAGGCAAA	KGK
90NLF1281118	NL		0	Wilp	52.21760	6.10586	accurate	*Lepus europaeus*	NA	0	1	0	NA	0	1	0	NA	NA	NA	NA	NA	NA	NA	NA	NA
91NLM1281118	NL		0	Wilp	52.21760	6.10586	accurate	*Lepus europaeus*	NA	0	0	1	NA	0	0	1	NA	NA	NA	NA	NA	NA	NA	NA	NA
92NLF1281118	NL		0	Wilp	52.21760	6.10586	accurate	*Lepus europaeus*	NA	0	0	1	NA	0	0	1	NA	NA	NA	NA	NA	NA	NA	NA	NA
93NLM1281118	NL		0	Wilp	52.21760	6.10586	accurate	*Lepus europaeus*	NA	0	0	1	NA	0	0	1	NA	NA	NA	NA	NA	NA	NA	NA	NA
94NLM1281118	NL		0	Wilp	52.21760	6.10586	accurate	*Lepus europaeus*	NA	0	1	0	NA	0	1	0	NA	NA	NA	NA	NA	NA	NA	NA	NA
95NLF1281118	NL		0	Wilp	52.21760	6.10586	accurate	*Lepus europaeus*	NA	0	0	1	NA	0	0	1	NA	NA	NA	NA	NA	NA	NA	NA	NA
96NLFX090119	NL		unknown	Utrecht	52.08516	5.17411	estimated	*Lepus europaeus*	NA	0	0	1	NA	0	0	1	NA	NA	NA	NA	NA	NA	NA	NA	NA
97NLFX090119	NL		unknown	Utrecht	52.08516	5.17411	estimated	*Lepus europaeus*	NA	0	0	1	NA	0	0	1	NA	NA	NA	NA	NA	NA	NA	NA	NA
98NLMU290519	NL		1	Utrecht	52.08516	5.17411	estimated	*Lepus europaeus*	**ON089525**	1	0	0	OM939694	1	0	0	**OM991049**	GGAGGC	GG	AAAGGTGGAGGC	KGGG	I	2	AAA	K
01GBMX060219	GB		unknown	Somerset	51.467740	-2.7845384	accurate	*Lepus europaeus*	NA	0	0	1	NA	0	1	0	NA	NA	NA	NA	NA	NA	NA	NA	NA
02GBFX010319	GB		unknown	Hampshire	51.149161	-1.1764564	accurate	*Lepus europaeus*	NA	0	0	1	NA	0	0	1	NA	NA	NA	NA	NA	NA	NA	NA	NA
03GBFX020319	GB		unknown	Wiltshire	51.383957	-1.8548170	accurate	*Lepus europaeus*	**ON089373**	0	0	1	NA	1	0	0	**OM991054**	GGAGGC	GG	AAAGGTGGAGGC	KGGG	I	4	AAA	K
04GBFX030319	GB		unknown	Wiltshire	51.380368	-1.8605757	accurate	*Lepus europaeus*	**ON089375**	0	1	0	NA	0	1	0	NA	NA	NA	NA	NA	NA	NA	NA	NA
05GBMX140819	GB		unknown	Dorset	50.699181	-2.5833173	accurate	*Lepus europaeus*	**ON089379**	1	0	0	OM939697	1	0	0	**OM991056**	GGAGGC	GG	AAAGGTGGAGGC	KGGG	I	2	AAA	K
06GBFX140619	GB		unknown	Wiltshire	51.497646	-1.4078866	accurate	*Lepus europaeus*	ON089569	0	1	0	NA	0	1	0	NA	NA	NA	NA	NA	NA	NA	NA	NA
07GBMX040719	GB		unknown	Gloucestershire	51.704711	-2.2777974	accurate	*Lepus europaeus*	**ON089386**	0	0	1	NA	1	0	0	**OM991059**	GGAGGC	GG	AAAGGTGGAGGC	KGGG	I	2	AAA	K
08GBMX160719	GB		unknown	Devon	50.652101	-3.2928449	accurate	*Lepus europaeus*	**ON089389**	1	0	0	OM939693	1	0	0	**OM991060**	GGAGGCAAAGGTGGAGTC	GGKGGV	AAAGGTGGAGGC	KGGG	NA	3	AAA	K
09GBFX010420	GB		unknown	Derbyshire	53.12704	-1.577045	estimated	*Lepus timidus*	NA	0	0	1	NA	0	0	1	NA	NA	NA	NA	NA	NA	NA	NA	NA
10GBMX010720	GB		unknown	Wiltshire	51.309999	-1.921564	estimated	*Lepus europaeus*	**ON089394**	0	0	1	NA	0	0	1	NA	NA	NA	NA	NA	NA	NA	NA	NA
11GBFX040720	GB		unknown	Dorset	50.811349	-2.294185	estimated	*Lepus europaeus*	NA	0	0	1	NA	0	0	1	NA	NA	NA	NA	NA	NA	NA	NA	NA
12GBMX011020	GB		unknown	Cambridgeshire	52.338160	0.056691	estimated	*Lepus europaeus*	**ON089400**	1	0	0	OM939693	1	0	0	**OM990891**	GGAGGC	GG	AAAGGTGGAGGC	KGGG	I	4	AAA	K
13GBMX021020	GB		unknown	Norfolk	52.687027	0.994806	estimated	*Lepus europaeus*	**ON089404**	1	0	0	OM939694	1	0	0	**OM990895**	GGAGGC	GG	AAAGGTGGAGGC	KGGG	I	2	AAA	K
14GBMX031020	GB		unknown	Suffolk	52.212865	1.055274	estimated	*Lepus europaeus*	**ON089409**	0	0	1	NA	1	0	0	OM990895	GGAGGC	GG	AAAGGTGGAGGC	KGGG	I	2	AAA	K
15GBMX041020	GB		unknown	Norfolk	52.687027	0.994806	estimated	*Lepus europaeus*	**ON089412**	1	0	0	OM939693	1	0	0	**OM990902**	GGAGGC	GG	AAAGGTGGAGGC	KGGG	I	2	AAA	K
16GBFX051020	GB		unknown	Suffolk	52.212865	1.055274	estimated	*Lepus europaeus*	**ON089418**	1	0	0	**OM939697**	1	0	0	**OM990910**	GGAGGC	GG	AAAGGTGGAGGC	KGGG	I	5	AAA	K
17GBMX061020	GB		unknown	Suffolk	52.212865	1.055274	estimated	*Lepus europaeus*	**ON089421**	0	0	1	NA	0	0	1	NA	NA	NA	NA	NA	NA	NA	NA	NA
18GBMX071020	GB		unknown	Suffolk	52.212865	1.055274	estimated	*Lepus europaeus*	**ON089424**	1	0	0	OM939697	0	1	0	NA	NA	NA	NA	NA	NA	NA	NA	NA
19GBMX081020	GB		unknown	Wiltshire	51.322980	-1.931893	estimated	*Oryctolagus cuniculus*	ON089430	0	1	0	NA	0	0	1	NA	NA	NA	NA	NA	NA	NA	NA	NA
20GBFX011120	GB		unknown	East Anglia	52.203795	0.554277	estimated	*Lepus europaeus*	**ON089427**	0	1	0	NA	0	0	1	NA	NA	NA	NA	NA	NA	NA	NA	NA
21GBFX021120	GB		unknown	East Anglia	52.203795	0.554277	estimated	*Oryctolagus cuniculus*	**ON089430**	0	0	1	NA	0	0	1	NA	NA	NA	NA	NA	NA	NA	NA	NA
22GBFX031120	GB		unknown	East Anglia	52.203795	0.554277	estimated	*Oryctolagus cuniculus*	**ON089433**	0	0	1	NA	0	0	1	NA	NA	NA	NA	NA	NA	NA	NA	NA
23GBFX041120	GB		unknown	East Anglia	52.203795	0.554277	estimated	*Oryctolagus cuniculus*	**ON089435**	0	1	0	NA	0	0	1	NA	NA	NA	NA	NA	NA	NA	NA	NA
24GBFX051120	GB		unknown	East Anglia	52.203795	0.554277	estimated	*Lepus europaeus*	**ON089437**	0	0	1	NA	0	0	1	NA	NA	NA	NA	NA	NA	NA	NA	NA
25GBFX061120	GB		unknown	East Anglia	52.203795	0.554277	estimated	*Oryctolagus cuniculus*	**ON089438**	0	0	1	NA	0	0	1	NA	NA	NA	NA	NA	NA	NA	NA	NA
G6	CZ		unknown	Brno	49.17717	16.56802	estimated	*Lepus europaeus*	NA	1	0	0	OM939694	1	0	0	**OM991075**	GGAGGC	GG	AAAGGTGGAGGC	KGGG	I	5	AAA	K
G7	CZ		unknown	Brno	49.17717	16.56802	estimated	*Lepus europaeus*	NA	1	0	0	OM939694	1	0	0	**OM991076**	GGAGGC	GG	AAAGGTGGAGGC	KGGG	I	6	AAA	K
G11	CZ		unknown	Brno	49.17717	16.56802	estimated	*Lepus europaeus*	NA	1	0	0	OM939694	1	0	0	**OM991077**	GGAGGCAAAGGTAAAGGC	GGKGKG	AAAGGTGGAGGC	KGGG	NA	1	AAA	K
G15	CZ		unknown	Brno	49.17717	16.56802	estimated	*Lepus europaeus*	NA	1	0	0	OM939694	1	0	0	**OM991078**	GGAGGCAAAGGTAAAGGC	GGKGKG	AAAGGTGGAGGC	KGGG	NA	1	AAA	K
G21	CZ		unknown	Brno	49.17717	16.56802	estimated	*Lepus europaeus*	NA	1	0	0	**OM939695**	1	0	0	**OM991079**	GGAGGC	GG	AAAGGTGGAGGC	KGGG	I	5	AAAGGCAAA	KGK
G22_5	CZ		unknown	Brno	49.17717	16.56802	estimated	*Lepus europaeus*	NA	NA	NA	NA	NA	NA	NA	NA	**OM991082**	GGAGGCAAAGGTAAAGGC	GGKGKG	AAAGGTGGAGGC	KGGG	NA	1	AAA	K
G22_1	CZ		unknown	Brno	49.17717	16.56802	estimated	*Lepus europaeus*	NA	NA	NA	NA	NA	NA	NA	NA	**OM991081**	GGAGGCAAAGGTAAAGGC	GGKGKG	AAAGGTGGAGGC	KGGG	NA	1	AAA	K
G22	CZ		unknown	Brno	49.17717	16.56802	estimated	*Lepus europaeus*	NA	1	0	0	OM939695	1	0	0	**OM991080**	GGAGGC	GG	AAAGGTGGAGGC	KGGG	I	4	AAA	K
G25	CZ		unknown	Brno	49.17717	16.56802	estimated	*Lepus europaeus*	NA	1	0	0	OM939694	1	0	0	**OM991083**	GGAGGCAAAGGTAAAGGC	GGKGKG	AAAGGTGGAGGC	KGGG	NA	1	AAA	K
G29	CZ		unknown	Brno	49.17717	16.56802	estimated	*Lepus europaeus*	NA	1	0	0	OM939695	1	0	0	**OM991084**	GGAGGC	GG	AAAGGTGGAGGC	KGGG	I	4	AAAGGCAAA	KGK
G30	CZ		unknown	Brno	49.17717	16.56802	estimated	*Lepus europaeus*	NA	1	0	0	OM939693	1	0	0	**OM991085**	GGAGGC	GG	AAAGGTGGAGGC	KGGG	I	4	AAA	K
G31	CZ		unknown	Brno	49.17717	16.56802	estimated	*Lepus europaeus*	NA	0	0	1	NA	1	0	0	**OM991086**	GGAGGC	GG	AAAGGTGGAGGC	KGGG	I	4	AAAGGCAAA	KGK
G36	CZ		unknown	Brno	49.17717	16.56802	estimated	*Lepus europaeus*	NA	1	0	0	OM939695	1	0	0	**OM991087**	GGAGGC	GG	AAAGGTGGAGGC	KGGG	I	5	AAAGGCAAA	KGK
G40	CZ		unknown	Brno	49.17717	16.56802	estimated	*Lepus europaeus*	NA	1	0	0	OM939695	1	0	0	**OM991088**	GGAGGC	GG	AAAGGTGGAGGC	KGGG	I	3	AAAGGCAAA	KGK
G43	CZ		unknown	Brno	49.17717	16.56802	estimated	*Lepus europaeus*	NA	1	0	0	OM939695	1	0	0	**OM991089**	GGAGGC	GG	AAAGGTGGAGGC	KGGG	I	5	AAAGGCAAA	KGK
G44	CZ		unknown	Brno	49.17717	16.56802	estimated	*Lepus europaeus*	NA	1	0	0	OM939695	1	0	0	OM991084	GGAGGC	GG	AAAGGTGGAGGC	KGGG	I	4	AAAGGCAAA	KGK
G60	CZ		unknown	Brno	49.17717	16.56802	estimated	*Lepus europaeus*	NA	1	0	0	OM939695	1	0	0	**OM991090**	GGAGGC	GG	AAAGGTGGAGGC	KGGG	I	5	AAAGGCAAA	KGK
01BVM1131118	GER-CW		0	Bad Vilbel	50.20873	8.73453	accurate	*Lepus europaeus*	NA	0	0	1	NA	0	0	1	NA	NA	NA	NA	NA	NA	NA	NA	NA
02BVM1131118	GER-CW		0	Bad Vilbel	50.20873	8.73453	accurate	*Lepus europaeus*	NA	0	0	1	NA	0	0	1	NA	NA	NA	NA	NA	NA	NA	NA	NA
03BVF1131118	GER-CW		0	Bad Vilbel	50.20873	8.73453	accurate	*Lepus europaeus*	NA	0	0	1	NA	0	0	1	NA	NA	NA	NA	NA	NA	NA	NA	NA
04BVF1131118	GER-CW		0	Bad Vilbel	50.20873	8.73453	accurate	*Lepus europaeus*	NA	0	0	1	NA	0	0	1	NA	NA	NA	NA	NA	NA	NA	NA	NA
05BVF2131118	GER-CW		1	Bad Vilbel	50.20873	8.73453	accurate	*Lepus europaeus*	NA	1	0	0	OM939693	1	0	0	**OM990866**	GGAGGC	GG	AAAGGTGGAGGC	KGGG	I	3	AAA	K
06BVF1131118	GER-CW		0	Bad Vilbel	50.20873	8.73453	accurate	*Lepus europaeus*	NA	0	1	0	NA	0	1	0	NA	NA	NA	NA	NA	NA	NA	NA	NA
07BVM2131118	GER-CW		0	Bad Vilbel	50.20873	8.73453	accurate	*Lepus europaeus*	NA	1	0	0	**OM939700**	1	0	0	**OM990874**	NA	NA	AAAGGCAAAGGT	KGKG	II	3	AAAGGCAAA	KGK
08BVF1131118	GER-CW		0	Bad Vilbel	50.20873	8.73453	accurate	*Lepus europaeus*	NA	1	0	0	OM939693	1	0	0	**OM990880**	GGAGGC	GG	AAAGGTGGAGGC	KGGG	I	2	AAA	K
09BVM1131118	GER-CW		0	Bad Vilbel	50.20873	8.73453	accurate	*Lepus europaeus*	NA	0	0	1	NA	0	0	1	NA	NA	NA	NA	NA	NA	NA	NA	NA
10BVF1131118	GER-CW		0	Bad Vilbel	50.20873	8.73453	accurate	*Lepus europaeus*	NA	0	0	1	NA	0	0	1	NA	NA	NA	NA	NA	NA	NA	NA	NA
11BVF1131118	GER-CW		0	Bad Vilbel	50.20873	8.73453	accurate	*Lepus europaeus*	NA	1	0	0	OM939693	1	0	0	**OM990886**	GGAGGC	GG	AAAGGTGGAGGC	KGGG	I	4	AAA	K
12BVM1131118	GER-CW		0	Bad Vilbel	50.20873	8.73453	accurate	*Lepus europaeus*	NA	0	0	1	NA	0	0	1	NA	NA	NA	NA	NA	NA	NA	NA	NA
13BVM1131118	GER-CW		0	Bad Vilbel	50.20873	8.73453	accurate	*Lepus europaeus*	NA	1	0	0	OM939694	1	0	0	**OM990893**	NA	NA	AAAGGCAAAGGT	KGKG	II	3	AAAGGCAAA	KGK
14BVF1131118	GER-CW		0	Bad Vilbel	50.20873	8.73453	accurate	*Lepus europaeus*	NA	0	1	0	NA	0	1	0	NA	NA	NA	NA	NA	NA	NA	NA	NA
15BVF1131118	GER-CW		0	Bad Vilbel	50.20873	8.73453	accurate	*Lepus europaeus*	NA	0	1	0	NA	0	1	0	NA	NA	NA	NA	NA	NA	NA	NA	NA
16BVF1131118	GER-CW		0	Bad Vilbel	50.20873	8.73453	accurate	*Lepus europaeus*	NA	0	0	1	NA	0	0	1	NA	NA	NA	NA	NA	NA	NA	NA	NA
17BVF1131118	GER-CW		0	Bad Vilbel	50.20873	8.73453	accurate	*Lepus europaeus*	NA	0	0	1	NA	0	0	1	NA	NA	NA	NA	NA	NA	NA	NA	NA
18BVM2131118	GER-CW		1	Bad Vilbel	50.20873	8.73453	accurate	*Lepus europaeus*	NA	1	0	0	OM939694	1	0	0	**OM990916**	NA	NA	AAAGGCAAAGGT	KGKG	II	3	AAAGGCAAA	KGK
19BVM1131118	GER-CW		0	Bad Vilbel	50.20873	8.73453	accurate	*Lepus europaeus*	NA	1	0	0	OM939694	1	0	0	**OM990917**	GGAGGC	GG	AAAGGTGGAGGC	KGGG	I	5	AAA	K
20BVF2131118	GER-CW		1	Bad Vilbel	50.20873	8.73453	accurate	*Lepus europaeus*	NA	0	1	0	NA	1	0	0	OM990866	GGAGGC	GG	AAAGGTGGAGGC	KGGG	I	3	AAA	K
21BVF1131118	GER-CW		0	Bad Vilbel	50.20873	8.73453	accurate	*Lepus europaeus*	NA	0	1	0	NA	0	1	0	NA	NA	NA	NA	NA	NA	NA	NA	NA
22BVF1131118	GER-CW		0	Bad Vilbel	50.20873	8.73453	accurate	*Lepus europaeus*	NA	1	0	0	OM939693	1	0	0	**OM990922**	NA	NA	AAAGGCGGAGGT	KGGG	I	4	AAAGGCAAA	KGK
23BVF1131118	GER-CW		0	Bad Vilbel	50.20873	8.73453	accurate	*Lepus europaeus*	NA	1	0	0	**OM939702**	1	0	0	**OM990927**	GGAGGC	GG	AAAGGTGGAGGC	KGGG	I	2	AAA	K
24BVF1131118	GER-CW		0	Bad Vilbel	50.20873	8.73453	accurate	*Lepus europaeus*	NA	0	0	1	NA	0	0	1	NA	NA	NA	NA	NA	NA	NA	NA	NA
25BVM1131118	GER-CW		0	Bad Vilbel	50.20873	8.73453	accurate	*Lepus europaeus*	NA	0	0	1	NA	0	0	1	NA	NA	NA	NA	NA	NA	NA	NA	NA

26BVM1131118	GER-CW		0	Bad Vilbel	50.20873	8.73453	accurate	*Lepus europaeus*	NA	0	0	1	NA	0	0	1	NA	NA	NA	NA	NA	NA	NA	NA	NA
27BVF1131118	GER-CW		0	Bad Vilbel	50.20873	8.73453	accurate	*Lepus europaeus*	NA	0	1	0	NA	0	0	1	NA	NA	NA	NA	NA	NA	NA	NA	NA
28BVF1131118	GER-CW		0	Bad Vilbel	50.20873	8.73453	accurate	*Lepus europaeus*	NA	0	1	0	NA	0	1	0	NA	NA	NA	NA	NA	NA	NA	NA	NA
29BVM1131118	GER-CW		0	Bad Vilbel	50.20873	8.73453	accurate	*Lepus europaeus*	NA	1	0	0	OM939693	1	0	0	**OM990939**	GGAGGC	GG	AAAGGTGGAGGC	KGGG	NA	1	AGAGGTGGAGGCAAA	RGGGK
30BVM1131118	GER-CW		0	Bad Vilbel	50.20873	8.73453	accurate	*Lepus europaeus*	NA	0	1	0	NA	0	1	0	NA	NA	NA	NA	NA	NA	NA	NA	NA
31BVM1131118	GER-CW		0	Bad Vilbel	50.20873	8.73453	accurate	*Lepus europaeus*	NA	0	0	1	NA	0	0	1	NA	NA	NA	NA	NA	NA	NA	NA	NA
32BVF1131118	GER-CW		0	Bad Vilbel	50.20873	8.73453	accurate	*Lepus europaeus*	NA	0	1	0	NA	0	1	0	NA	NA	NA	NA	NA	NA	NA	NA	NA
33BVF1131118	GER-CW		0	Bad Vilbel	50.20873	8.73453	accurate	*Lepus europaeus*	NA	0	0	1	NA	0	0	1	NA	NA	NA	NA	NA	NA	NA	NA	NA
34BVF1131118	GER-CW		0	Bad Vilbel	50.20873	8.73453	accurate	*Lepus europaeus*	NA	1	0	0	OM939701	1	0	0	**OM990946**	GGAGGC	GG	AAAGGTGGAGGC	KGGG	I	6	AAA	K
35BVM1131118	GER-CW		0	Bad Vilbel	50.20873	8.73453	accurate	*Lepus europaeus*	NA	0	0	1	NA	0	0	1	NA	NA	NA	NA	NA	NA	NA	NA	NA
36BVM1131118	GER-CW		0	Bad Vilbel	50.20873	8.73453	accurate	*Lepus europaeus*	NA	1	0	0	OM939693	1	0	0	**OM990948**	GGAGGC	GG	AAAGGTGGAGGC	KGGG	I	2	AAA	K
37BVF1131118	GER-CW		0	Bad Vilbel	50.20873	8.73453	accurate	*Lepus europaeus*	NA	1	0	0	OM939694	1	0	0	**OM990951**	GGAGGC	GG	AAAGGTGGAGGC	KGGG	I	2	AAA	K
38BVM1131118	GER-CW		0	Bad Vilbel	50.20873	8.73453	accurate	*Lepus europaeus*	NA	0	1	0	NA	0	1	0	NA	NA	NA	NA	NA	NA	NA	NA	NA
39BVM1131118	GER-CW		0	Bad Vilbel	50.20873	8.73453	accurate	*Lepus europaeus*	NA	1	0	0	OM939702	1	0	0	**OM990953**	NA	NA	AAAGGCGGAGGT	KGGG	I	6	AAAGGCAAA	KGK
40BVM1131118	GER-CW		0	Bad Vilbel	50.20873	8.73453	accurate	*Lepus europaeus*	NA	0	0	1	NA	0	1	0	NA	NA	NA	NA	NA	NA	NA	NA	NA
41BVM1141118	GER-CW		0	Bad Vilbel	50.20873	8.73453	accurate	*Lepus europaeus*	NA	0	0	1	NA	0	0	1	NA	NA	NA	NA	NA	NA	NA	NA	NA
42BVF1141118	GER-CW		0	Bad Vilbel	50.20873	8.73453	accurate	*Lepus europaeus*	NA	0	0	1	NA	0	0	1	NA	NA	NA	NA	NA	NA	NA	NA	NA
43BVF1141118	GER-CW		0	Bad Vilbel	50.20873	8.73453	accurate	*Lepus europaeus*	NA	0	0	1	NA	0	0	1	NA	NA	NA	NA	NA	NA	NA	NA	NA
44BVF1141118	GER-CW		0	Bad Vilbel	50.20873	8.73453	accurate	*Lepus europaeus*	NA	0	0	1	NA	0	0	1	NA	NA	NA	NA	NA	NA	NA	NA	NA
45BVM1141118	GER-CW		0	Bad Vilbel	50.20873	8.73453	accurate	*Lepus europaeus*	NA	0	0	1	NA	0	0	1	NA	NA	NA	NA	NA	NA	NA	NA	NA
46BVM1141118	GER-CW		0	Bad Vilbel	50.20873	8.73453	accurate	*Lepus europaeus*	NA	0	0	1	NA	0	0	1	NA	NA	NA	NA	NA	NA	NA	NA	NA
47BVF1141118	GER-CW		0	Bad Vilbel	50.20873	8.73453	accurate	*Lepus europaeus*	NA	0	0	1	NA	0	0	1	NA	NA	NA	NA	NA	NA	NA	NA	NA
48BVF1141118	GER-CW		0	Bad Vilbel	50.20873	8.73453	accurate	*Lepus europaeus*	NA	0	1	0	NA	0	0	1	NA	NA	NA	NA	NA	NA	NA	NA	NA
49BVF1141118	GER-CW		0	Bad Vilbel	50.20873	8.73453	accurate	*Lepus europaeus*	NA	0	0	1	NA	0	0	1	NA	NA	NA	NA	NA	NA	NA	NA	NA
50BVM1141118	GER-CW		0	Bad Vilbel	50.20873	8.73453	accurate	*Lepus europaeus*	NA	1	0	0	OM939694	1	0	0	**OM990969**	GAAGGC	EG	AAAGGTGGAGGC	KGGG	I	2	AAAGGCAAA	KGK
51BVF1141118	GER-CW		0	Bad Vilbel	50.20873	8.73453	accurate	*Lepus europaeus*	NA	0	1	0	NA	0	1	0	NA	NA	NA	NA	NA	NA	NA	NA	NA
52BVM1141118	GER-CW		0	Bad Vilbel	50.20873	8.73453	accurate	*Lepus europaeus*	NA	0	0	1	NA	0	0	1	NA	GGAGGC	GG	AAAGGTGGAGGC	KGGG	I	3	AAA	K
53BVM1141118	GER-CW		0	Bad Vilbel	50.20873	8.73453	accurate	*Lepus europaeus*	NA	0	0	1	NA	0	0	1	NA	NA	NA	NA	NA	NA	NA	NA	NA
54BVM1141118	GER-CW		0	Bad Vilbel	50.20873	8.73453	accurate	*Lepus europaeus*	NA	0	1	0	NA	0	0	1	NA	NA	NA	NA	NA	NA	NA	NA	NA
55BVM1141118	GER-CW		0	Bad Vilbel	50.20873	8.73453	accurate	*Lepus europaeus*	NA	0	1	0	NA	0	0	1	NA	NA	NA	NA	NA	NA	NA	NA	NA
56BVF1141118	GER-CW		0	Bad Vilbel	50.20873	8.73453	accurate	*Lepus europaeus*	NA	0	1	0	NA	0	0	1	NA	NA	NA	NA	NA	NA	NA	NA	NA
57BVM1141118	GER-CW		0	Bad Vilbel	50.20873	8.73453	accurate	*Lepus europaeus*	NA	0	0	1	NA	1	0	0	**OM990975**	GGAGGC	GG	AAAGGTGGAGGC	KGGG	I	3	AAA	K
58BVF1141118	GER-CW		0	Bad Vilbel	50.20873	8.73453	accurate	*Lepus europaeus*	NA	0	0	1	NA	0	0	1	NA	NA	NA	NA	NA	NA	NA	NA	NA
59BVM1141118	GER-CW		0	Bad Vilbel	50.20873	8.73453	accurate	*Lepus europaeus*	NA	0	1	0	NA	0	0	1	NA	NA	NA	NA	NA	NA	NA	NA	NA
60BVM1141118	GER-CW		0	Bad Vilbel	50.20873	8.73453	accurate	*Lepus europaeus*	NA	0	0	1	NA	0	0	1	NA	NA	NA	NA	NA	NA	NA	NA	NA
61BVM1141118	GER-CW		0	Bad Vilbel	50.20873	8.73453	accurate	*Lepus europaeus*	NA	0	0	1	NA	0	0	1	NA	NA	NA	NA	NA	NA	NA	NA	NA
62BVF1141118	GER-CW		0	Bad Vilbel	50.20873	8.73453	accurate	*Lepus europaeus*	NA	0	1	0	NA	0	0	1	NA	NA	NA	NA	NA	NA	NA	NA	NA
63BVM1141118	GER-CW		0	Bad Vilbel	50.20873	8.73453	accurate	*Lepus europaeus*	NA	0	1	0	NA	0	0	1	NA	NA	NA	NA	NA	NA	NA	NA	NA
64BVF1141118	GER-CW		0	Bad Vilbel	50.20873	8.73453	accurate	*Lepus europaeus*	NA	0	0	1	NA	0	0	1	NA	NA	NA	NA	NA	NA	NA	NA	NA
65BVF1141118	GER-CW		0	Bad Vilbel	50.20873	8.73453	accurate	*Lepus europaeus*	NA	1	0	0	OM939694	1	0	0	**OM990988**	GGAGGC	GG	AAAGGTGGAGGC	KGGG	I	5	AAA	K
66BVF1141118	GER-CW		0	Bad Vilbel	50.20873	8.73453	accurate	*Lepus europaeus*	NA	0	1	0	NA	0	0	1	NA	NA	NA	NA	NA	NA	NA	NA	NA
67BVF1121119	GER-CW		0	Bad Vilbel	50.20873	8.73453	accurate	*Lepus europaeus*	NA	0	0	1	NA	0	0	1	NA	NA	NA	NA	NA	NA	NA	NA	NA
68BVM1121119	GER-CW		0	Bad Vilbel	50.20873	8.73453	accurate	*Lepus europaeus*	NA	1	0	0	OM939694	1	0	0	**OM990994**	NA	NA	AAAGGCAAAGGT	KGKG	II	3	AAAGGCAAA	KGK
69BVM2121119	GER-CW		0	Bad Vilbel	50.20873	8.73453	accurate	*Lepus europaeus*	NA	0	1	0	NA	0	0	1	NA	NA	NA	NA	NA	NA	NA	NA	NA
70BVM1121119	GER-CW		0	Bad Vilbel	50.20873	8.73453	accurate	*Lepus europaeus*	NA	0	0	1	NA	0	0	1	NA	NA	NA	NA	NA	NA	NA	NA	NA
71BVF1121119	GER-CW		0	Bad Vilbel	50.20873	8.73453	accurate	*Lepus europaeus*	NA	0	1	0	NA	0	1	0	NA	NA	NA	NA	NA	NA	NA	NA	NA
72BVF1121119	GER-CW		0	Bad Vilbel	50.20873	8.73453	accurate	*Lepus europaeus*	NA	1	0	0	OM939693	1	0	0	OM990866	GGAGGC	GG	AAAGGTGGAGGC	KGGG	I	3	AAA	K
73BVF1121119	GER-CW		0	Bad Vilbel	50.20873	8.73453	accurate	*Lepus europaeus*	NA	0	0	1	NA	0	0	1	NA	NA	NA	NA	NA	NA	NA	NA	NA
74BVM1121119	GER-CW		0	Bad Vilbel	50.20873	8.73453	accurate	*Lepus europaeus*	NA	1	0	0	OM939694	1	0	0	**OM990998**	NA	NA	AAAGGCAAAGGT	KGKG	II	3	AAAGGCAAA	KGK
75BVF1121119	GER-CW		0	Bad Vilbel	50.20873	8.73453	accurate	*Lepus europaeus*	NA	0	0	1	NA	0	0	1	NA	NA	NA	NA	NA	NA	NA	NA	NA
76BVM1121119	GER-CW		0	Bad Vilbel	50.20873	8.73453	accurate	*Lepus europaeus*	NA	0	0	1	NA	0	0	1	NA	NA	NA	NA	NA	NA	NA	NA	NA
77BVF1121119	GER-CW		0	Bad Vilbel	50.20873	8.73453	accurate	*Lepus europaeus*	NA	0	1	0	NA	0	1	0	NA	NA	NA	NA	NA	NA	NA	NA	NA
78BVM1121119	GER-CW		0	Bad Vilbel	50.20873	8.73453	accurate	*Lepus europaeus*	NA	0	0	1	NA	0	0	1	NA	NA	NA	NA	NA	NA	NA	NA	NA
79BVF1121119	GER-CW		0	Bad Vilbel	50.20873	8.73453	accurate	*Lepus europaeus*	NA	0	1	0	NA	0	1	0	NA	NA	NA	NA	NA	NA	NA	NA	NA
80BVF1121119	GER-CW		0	Bad Vilbel	50.20873	8.73453	accurate	*Lepus europaeus*	NA	0	0	1	NA	0	0	1	NA	NA	NA	NA	NA	NA	NA	NA	NA
81BVF1121119	GER-CW		0	Bad Vilbel	50.20873	8.73453	accurate	*Lepus europaeus*	NA	0	0	1	NA	0	0	1	NA	NA	NA	NA	NA	NA	NA	NA	NA
82BVM1121119	GER-CW		0	Bad Vilbel	50.20873	8.73453	accurate	*Lepus europaeus*	NA	0	1	0	NA	0	1	0	NA	NA	NA	NA	NA	NA	NA	NA	NA
83BVF1121119	GER-CW		0	Bad Vilbel	50.20873	8.73453	accurate	*Lepus europaeus*	NA	0	1	0	NA	0	1	0	NA	NA	NA	NA	NA	NA	NA	NA	NA
84BVF1121119	GER-CW		0	Bad Vilbel	50.20873	8.73453	accurate	*Lepus europaeus*	NA	0	0	1	NA	0	0	1	NA	NA	NA	NA	NA	NA	NA	NA	NA
85BVM1121119	GER-CW		0	Bad Vilbel	50.20873	8.73453	accurate	*Lepus europaeus*	NA	0	0	1	NA	0	0	1	NA	NA	NA	NA	NA	NA	NA	NA	NA
87BVM1121119	GER-CW		0	Bad Vilbel	50.20873	8.73453	accurate	*Lepus europaeus*	NA	0	0	1	NA	0	0	1	NA	NA	NA	NA	NA	NA	NA	NA	NA
88BVF1121119	GER-CW		0	Bad Vilbel	50.20873	8.73453	accurate	*Lepus europaeus*	NA	0	1	0	NA	0	1	0	NA	NA	NA	NA	NA	NA	NA	NA	NA
89BVF1121119	GER-CW		0	Bad Vilbel	50.20873	8.73453	accurate	*Lepus europaeus*	NA	0	0	1	NA	0	0	1	NA	NA	NA	NA	NA	NA	NA	NA	NA
90BVM1121119	GER-CW		0	Bad Vilbel	50.20873	8.73453	accurate	*Lepus europaeus*	NA	0	0	1	NA	0	0	1	NA	NA	NA	NA	NA	NA	NA	NA	NA
91BVM1121119	GER-CW		0	Bad Vilbel	50.20873	8.73453	accurate	*Lepus europaeus*	NA	0	0	1	NA	0	0	1	NA	NA	NA	NA	NA	NA	NA	NA	NA
92BVF1121119	GER-CW		0	Bad Vilbel	50.20873	8.73453	accurate	*Lepus europaeus*	NA	0	1	0	NA	0	1	0	NA	NA	NA	NA	NA	NA	NA	NA	NA
93BVF1121119	GER-CW		0	Bad Vilbel	50.20873	8.73453	accurate	*Lepus europaeus*	NA	0	0	1	NA	0	0	1	NA	NA	NA	NA	NA	NA	NA	NA	NA
94BVM1121119	GER-CW		0	Bad Vilbel	50.20873	8.73453	accurate	*Lepus europaeus*	NA	0	1	0	NA	1	0	0	**OM991017**	GGAGGC	GG	AAAGGTGGAGGC	KGGG	I	1	AAA	K
95BVM1121119	GER-CW		0	Bad Vilbel	50.20873	8.73453	accurate	*Lepus europaeus*	NA	1	0	0	OM939693	1	0	0	**OM991018**	GGAGGC	GG	AAAGGTGGAGGC	KGGG	I	3	AAA	K
96BVF1121119	GER-CW		0	Bad Vilbel	50.20873	8.73453	accurate	*Lepus europaeus*	NA	0	0	1	NA	0	0	1	NA	NA	NA	NA	NA	NA	NA	NA	NA
97BVF1121119	GER-CW		0	Bad Vilbel	50.20873	8.73453	accurate	*Lepus europaeus*	NA	0	0	1	NA	0	0	1	NA	NA	NA	NA	NA	NA	NA	NA	NA
98BVF1121119	GER-CW		0	Bad Vilbel	50.20873	8.73453	accurate	*Lepus europaeus*	NA	0	0	1	NA	1	0	0	**OM991023**	GGAGGCAAAGTTGGAGGC	GGKVGG	AAAGGTGGAGGC	KGGG	NA	1	AAA	K
99BVF1121119	GER-CW		0	Bad Vilbel	50.20873	8.73453	accurate	*Lepus europaeus*	NA	0	0	1	NA	0	0	1	NA	NA	NA	NA	NA	NA	NA	NA	NA
100BVF1121119	GER-CW		0	Bad Vilbel	50.20873	8.73453	accurate	*Lepus europaeus*	NA	1	0	0	OM939694	1	0	0	**OM991025**	GGAGGC	GG	AAAGGTGGAGGC	KGGG	I	4	AAA	K
101BVF1121119	GER-CW		0	Bad Vilbel	50.20873	8.73453	accurate	*Lepus europaeus*	NA	0	0	1	NA	0	0	1	NA	NA	NA	NA	NA	NA	NA	NA	NA
102BVF1121119	GER-CW		0	Bad Vilbel	50.20873	8.73453	accurate	*Lepus europaeus*	NA	0	0	1	NA	0	0	1	NA	NA	NA	NA	NA	NA	NA	NA	NA
103BVF1131119	GER-CW		0	Bad Vilbel	50.20873	8.73453	accurate	*Lepus europaeus*	NA	0	0	1	NA	0	0	1	NA	NA	NA	NA	NA	NA	NA	NA	NA
104BVF1131119	GER-CW		0	Bad Vilbel	50.20873	8.73453	accurate	*Lepus europaeus*	NA	0	0	1	NA	0	0	1	NA	NA	NA	NA	NA	NA	NA	NA	NA
105BVF1131119	GER-CW		0	Bad Vilbel	50.20873	8.73453	accurate	*Lepus europaeus*	NA	0	0	1	NA	0	0	1	NA	NA	NA	NA	NA	NA	NA	NA	NA
106BVM1131119	GER-CW		0	Bad Vilbel	50.20873	8.73453	accurate	*Lepus europaeus*	NA	0	0	1	NA	0	0	1	NA	NA	NA	NA	NA	NA	NA	NA	NA
107BVM1131119	GER-CW		0	Bad Vilbel	50.20873	8.73453	accurate	*Lepus europaeus*	NA	0	0	1	NA	0	0	1	NA	NA	NA	NA	NA	NA	NA	NA	NA
108BVM1131119	GER-CW		0	Bad Vilbel	50.20873	8.73453	accurate	*Lepus europaeus*	NA	0	0	1	NA	0	0	1	NA	NA	NA	NA	NA	NA	NA	NA	NA
109BVF1131119	GER-CW		0	Bad Vilbel	50.20873	8.73453	accurate	*Lepus europaeus*	NA	0	0	1	NA	0	0	1	NA	NA	NA	NA	NA	NA	NA	NA	NA
110BVF1131119	GER-CW		0	Bad Vilbel	50.20873	8.73453	accurate	*Lepus europaeus*	NA	1	0	0	OM939694	1	0	0	**OM991034**	GGAGGC	GG	AAAGGTGGAGGC	KGGG	I	2	AAA	K
111BVM1131119	GER-CW		0	Bad Vilbel	50.20873	8.73453	accurate	*Lepus europaeus*	NA	0	0	1	NA	0	0	1	NA	NA	NA	NA	NA	NA	NA	NA	NA
112BVM1131119	GER-CW		0	Bad Vilbel	50.20873	8.73453	accurate	*Lepus europaeus*	NA	1	0	0	OM939693	1	0	0	**OM991036**	GAAGGC	EG	AAAGGTGGAGGC	KGGG	I	1	AAAGGCAAA	KGK
113BVF1131119	GER-CW		0	Bad Vilbel	50.20873	8.73453	accurate	*Lepus europaeus*	NA	0	0	1	NA	0	0	1	NA	NA	NA	NA	NA	NA	NA	NA	NA
114BVM2131119	GER-CW		0	Bad Vilbel	50.20873	8.73453	accurate	*Lepus europaeus*	NA	0	1	0	NA	0	1	0	NA	NA	NA	NA	NA	NA	NA	NA	NA
115BVF1131119	GER-CW		0	Bad Vilbel	50.20873	8.73453	accurate	*Lepus europaeus*	NA	1	0	0	OM939693	1	0	0	**OM991038**	GAAGGC	EG	AAAGGTGGAGGC	KGGG	I	2	AAAGGCAAA	KGK
116BVF1131119	GER-CW		0	Bad Vilbel	50.20873	8.73453	accurate	*Lepus europaeus*	NA	0	1	0	NA	0	0	1	NA	NA	NA	NA	NA	NA	NA	NA	NA
117BVM1131119	GER-CW		0	Bad Vilbel	50.20873	8.73453	accurate	*Lepus europaeus*	NA	0	0	1	NA	0	0	1	NA	NA	NA	NA	NA	NA	NA	NA	NA
118BVF1131119	GER-CW		0	Bad Vilbel	50.20873	8.73453	accurate	*Lepus europaeus*	NA	0	0	1	NA	0	0	1	NA	NA	NA	NA	NA	NA	NA	NA	NA
119BVM1201119	GER-CW		0	Niedererlenbach	50.22660	8.71156	accurate	*Lepus europaeus*	NA	1	0	0	OM939694	1	0	0	**OM991041**	GGAGGC	GG	AAAGGTGGAGGC	KGGG	I	4	AAA	K
120BVM1201119	GER-CW		0	Niedererlenbach	50.22660	8.71156	accurate	*Lepus europaeus*	NA	0	1	0	NA	0	1	0	NA	NA	NA	NA	NA	NA	NA	NA	NA
121BVF1201119	GER-CW		0	Niedererlenbach	50.22660	8.71156	accurate	*Lepus europaeus*	NA	0	1	0	NA	0	1	0	NA	NA	NA	NA	NA	NA	NA	NA	NA
122BVF1201119	GER-CW		0	Niedererlenbach	50.22660	8.71156	accurate	*Lepus europaeus*	NA	0	1	0	NA	0	1	0	NA	NA	NA	NA	NA	NA	NA	NA	NA
123BVF1201119	GER-CW		0	Niedererlenbach	50.22660	8.71156	accurate	*Lepus europaeus*	NA	1	0	0	OM939693	1	0	0	**OM991045**	GGAGGC	GG	AAAGGTGGAGGC	KGGG	NA	3	AGAGGTGGAGGCAAA	RGGGK
124BVM1201119	GER-CW		0	Niedererlenbach	50.22660	8.71156	accurate	*Lepus europaeus*	NA	1	0	0	OM939694	1	0	0	**OM991047**	GGAGGC	GG	AAAGGTGGAGGC	KGGG	I	3	AAA	K
125BVM1201119	GER-CW		0	Niedererlenbach	50.22660	8.71156	accurate	*Lepus europaeus*	NA	1	0	0	OM939694	1	0	0	**OM991048**	GGAGGC	GG	AAAGGTGGAGGC	KGGG	I	4	AAA	K
126BVM1201119	GER-CW		0	Niedererlenbach	50.22660	8.71156	accurate	*Lepus europaeus*	NA	0	0	1	NA	0	0	1	NA	NA	NA	NA	NA	NA	NA	NA	NA
127BVM1201119	GER-CW		0	Niedererlenbach	50.22660	8.71156	accurate	*Lepus europaeus*	NA	1	0	0	OM939694	1	0	0	**OM991050**	GGAGGC	GG	AAAGGTAAAGGC	KGKG	II	3	AAA	K
128BVF1201119	GER-CW		0	Niedererlenbach	50.22660	8.71156	accurate	*Lepus europaeus*	NA	0	1	0	NA	1	0	0	**OM991053**	GGAGGC	GG	AAAGGTAAAGGC	KGKG	II	3	AAA	K

129BVM1201119	GER-CW		0	Niedererlenbach	50.22660	8.71156	accurate	*Lepus europaeus*	NA	0	0	1	NA	1	0	0	OM991048	GGAGGC	GG	AAAGGTGGAGGC	KGGG	I	4	AAA	K
130BVF1201119	GER-CW		0	Niedererlenbach	50.22660	8.71156	accurate	*Lepus europaeus*	NA	0	0	1	NA	0	0	1	NA	NA	NA	NA	NA	NA	NA	NA	NA
131BVF1201119	GER-CW		0	Niedererlenbach	50.22660	8.71156	accurate	*Lepus europaeus*	NA	0	0	1	NA	0	0	1	NA	NA	NA	NA	NA	NA	NA	NA	NA
132BVM1201119	GER-CW		0	Niedererlenbach	50.22660	8.71156	accurate	*Lepus europaeus*	NA	0	0	1	NA	0	0	1	NA	NA	NA	NA	NA	NA	NA	NA	NA
133BVM1201119	GER-CW		0	Niedererlenbach	50.22660	8.71156	accurate	*Lepus europaeus*	NA	0	1	0	NA	1	0	0	**OM991058**	GGAGGC	GG	AGAGGTGGAGGC	RGGG	NA	3	AAA	K
134BVM1201119	GER-CW		0	Niedererlenbach	50.22660	8.71156	accurate	*Lepus europaeus*	NA	0	1	0	NA	0	1	0	NA	NA	NA	NA	NA	NA	NA	NA	NA
135BVF1201119	GER-CW		0	Niedererlenbach	50.22660	8.71156	accurate	*Lepus europaeus*	NA	0	0	1	NA	0	0	1	NA	NA	NA	NA	NA	NA	NA	NA	NA
136BVF1201119	GER-CW		0	Niedererlenbach	50.22660	8.71156	accurate	*Lepus europaeus*	NA	0	0	1	NA	0	0	1	NA	NA	NA	NA	NA	NA	NA	NA	NA
137BVM1201119	GER-CW		0	Niedererlenbach	50.22660	8.71156	accurate	*Lepus europaeus*	NA	1	0	0	OM939693	1	0	0	**OM991061**	GAAGGC	EG	AAAGGTGGAGGC	KGGG	I	3	AAAGGCAAA	KGK
138BVM1201119	GER-CW		0	Niedererlenbach	50.22660	8.71156	accurate	*Lepus europaeus*	NA	0	1	0	NA	0	1	0	NA	NA	NA	NA	NA	NA	NA	NA	NA
139BVF1201119	GER-CW		0	Niedererlenbach	50.22660	8.71156	accurate	*Lepus europaeus*	NA	0	1	0	NA	0	1	0	NA	NA	NA	NA	NA	NA	NA	NA	NA
140BVF1201119	GER-CW		0	Niedererlenbach	50.22660	8.71156	accurate	*Lepus europaeus*	NA	0	1	0	NA	0	0	1	NA	NA	NA	NA	NA	NA	NA	NA	NA
141BVF1201119	GER-CW		0	Niedererlenbach	50.22660	8.71156	accurate	*Lepus europaeus*	NA	0	0	1	NA	0	0	1	NA	NA	NA	NA	NA	NA	NA	NA	NA
142BVF1201119	GER-CW		0	Niedererlenbach	50.22660	8.71156	accurate	*Lepus europaeus*	NA	0	0	1	NA	0	0	1	NA	NA	NA	NA	NA	NA	NA	NA	NA
143BVF1201119	GER-CW		0	Niedererlenbach	50.22660	8.71156	accurate	*Lepus europaeus*	NA	0	1	0	NA	0	1	0	NA	NA	NA	NA	NA	NA	NA	NA	NA
144BVF1201119	GER-CW		0	Niedererlenbach	50.22660	8.71156	accurate	*Lepus europaeus*	NA	0	1	0	NA	0	1	0	NA	NA	NA	NA	NA	NA	NA	NA	NA
145BVF1201119	GER-CW		0	Niedererlenbach	50.22660	8.71156	accurate	*Lepus europaeus*	NA	1	0	0	OM939693	1	0	0	**OM991065**	GGAGGC	GG	AAAGGTGGAGGC	KGGG	I	3	AAA	K
146BVM1201119	GER-CW		0	Niedererlenbach	50.22660	8.71156	accurate	*Lepus europaeus*	NA	0	1	0	NA	1	0	0	**OM991067**	GGAGGC	GG	AAAGGTGGAGGC	KGGG	I	2	AAA	K
147BVF1201119	GER-CW		0	Niedererlenbach	50.22660	8.71156	accurate	*Lepus europaeus*	NA	0	1	0	NA	0	1	0	NA	NA	NA	NA	NA	NA	NA	NA	NA
148BVF1201119	GER-CW		0	Niedererlenbach	50.22660	8.71156	accurate	*Lepus europaeus*	NA	0	1	0	NA	0	1	0	NA	NA	NA	NA	NA	NA	NA	NA	NA
149BVM1201119	GER-CW		0	Niedererlenbach	50.22660	8.71156	accurate	*Lepus europaeus*	NA	0	0	1	NA	0	0	1	NA	NA	NA	NA	NA	NA	NA	NA	NA
150BVF1201119	GER-CW		0	Niedererlenbach	50.22660	8.71156	accurate	*Lepus europaeus*	NA	0	1	0	NA	0	0	1	NA	NA	NA	NA	NA	NA	NA	NA	NA
151BVF1201119	GER-CW		0	Niedererlenbach	50.22660	8.71156	accurate	*Lepus europaeus*	NA	1	0	0	OM939704	1	0	0	OM991047	GGAGGC	GG	AAAGGTGGAGGC	KGGG	I	3	AAA	K
152BVM1201119	GER-CW		0	Niedererlenbach	50.22660	8.71156	accurate	*Lepus europaeus*	NA	0	1	0	NA	0	1	0	NA	NA	NA	NA	NA	NA	NA	NA	NA
153BVF1201119	GER-CW		0	Niedererlenbach	50.22660	8.71156	accurate	*Lepus europaeus*	NA	0	0	1	NA	0	0	1	NA	NA	NA	NA	NA	NA	NA	NA	NA
96EBF1311218	GER-CW		0	Hullersen	51.81095	9.80442	accurate	*Lepus europaeus*	NA	0	1	0	NA	0	0	1	NA	NA	NA	NA	NA	NA	NA	NA	NA
97EBF1311218	GER-CW		0	Hullersen	51.81095	9.80442	accurate	*Lepus europaeus*	NA	0	1	0	NA	0	1	0	NA	NA	NA	NA	NA	NA	NA	NA	NA
98EBM1311218	GER-CW		0	Hullersen	51.81095	9.80442	accurate	*Lepus europaeus*	NA	0	0	1	NA	0	0	1	NA	NA	NA	NA	NA	NA	NA	NA	NA
99EBM1311218	GER-CW		0	Hullersen	51.81095	9.80442	accurate	*Lepus europaeus*	NA	0	0	1	NA	0	0	1	NA	NA	NA	NA	NA	NA	NA	NA	NA
100EBF1311218	GER-CW		0	Hullersen	51.81095	9.80442	accurate	*Lepus europaeus*	NA	1	0	0	OM939694	1	0	0	**OM991026**	GGAGGC	GG	AAAGGTGGAGGC	KGGG	I	3	AAAGGCAAA	KGK
101EBF1311218	GER-CW		0	Hullersen	51.81095	9.80442	accurate	*Lepus europaeus*	NA	0	1	0	NA	0	1	0	NA	NA	NA	NA	NA	NA	NA	NA	NA
102EBF1311218	GER-CW		0	Hullersen	51.81095	9.80442	accurate	*Lepus europaeus*	NA	0	1	0	NA	0	1	0	NA	NA	NA	NA	NA	NA	NA	NA	NA
103EBM1311218	GER-CW		0	Hullersen	51.81095	9.80442	accurate	*Lepus europaeus*	NA	0	0	1	NA	0	0	1	NA	NA	NA	NA	NA	NA	NA	NA	NA
104EBF1311218	GER-CW		0	Hullersen	51.81095	9.80442	accurate	*Lepus europaeus*	NA	0	1	0	NA	0	1	0	NA	NA	NA	NA	NA	NA	NA	NA	NA
105EBF1311218	GER-CW		0	Hullersen	51.81095	9.80442	accurate	*Lepus europaeus*	NA	0	1	0	NA	0	1	0	NA	NA	NA	NA	NA	NA	NA	NA	NA
107EBF1311218	GER-CW		0	Hullersen	51.81095	9.80442	accurate	*Lepus europaeus*	NA	0	0	1	NA	1	0	0	**OM991031**	GGAGGC	GG	AAAGGTGGAGGC	KGGG	I	1	AAA	K
108EBF1311218	GER-CW		0	Hullersen	51.81095	9.80442	accurate	*Lepus europaeus*	NA	0	1	0	NA	0	1	0	NA	NA	NA	NA	NA	NA	NA	NA	NA
109EBF1311218	GER-CW		0	Hullersen	51.81095	9.80442	accurate	*Lepus europaeus*	NA	0	0	1	NA	1	0	0	**OM991032**	NA	NA	AAAGGC	KG	NA	3	AAAGGCGGAGGCAAA	KGGGK
111EBF1311218	GER-CW		0	Hullersen	51.81095	9.80442	accurate	*Lepus europaeus*	NA	1	0	0	OM939693	1	0	0	**OM991035**	GGAGGC	GG	AAAGGTGGAGGC	KGGG	I	2	AAA	K
112EBM1311218	GER-CW		0	Hullersen	51.81095	9.80442	accurate	*Lepus europaeus*	NA	0	0	1	NA	0	0	1	NA	NA	NA	NA	NA	NA	NA	NA	NA
113EBF1131218	GER-CW		0	Hullersen	51.81095	9.80442	accurate	*Lepus europaeus*	NA	0	1	0	NA	0	1	0	NA	NA	NA	NA	NA	NA	NA	NA	NA
114EBM1311218	GER-CW		0	Hullersen	51.81095	9.80442	accurate	*Lepus europaeus*	NA	0	0	1	NA	0	0	1	NA	NA	NA	NA	NA	NA	NA	NA	NA
115EBF1301219	GER-CW		0	Hullersen	51.81095	9.80442	accurate	*Lepus europaeus*	NA	0	1	0	NA	0	1	0	NA	NA	NA	NA	NA	NA	NA	NA	NA
116EBF1301219	GER-CW		0	Hullersen	51.81095	9.80442	accurate	*Lepus europaeus*	NA	0	1	0	NA	0	1	0	NA	NA	NA	NA	NA	NA	NA	NA	NA
117EBM1301219	GER-CW		0	Hullersen	51.81095	9.80442	accurate	*Lepus europaeus*	NA	0	0	1	NA	0	0	1	NA	NA	NA	NA	NA	NA	NA	NA	NA
118EBF1301219	GER-CW		0	Hullersen	51.81095	9.80442	accurate	*Lepus europaeus*	NA	0	0	1	NA	1	0	0	**OM991040**	GGAGGC	GG	AAAGGTGGAGGC	KGGG	I	1	AAAGGCAAA	KGK
119EBM1301219	GER-CW		0	Hullersen	51.81095	9.80442	accurate	*Lepus europaeus*	NA	1	0	0	OM939694	1	0	0	**OM991042**	NA	NA	AAAGGC	KG	NA	3	AAAGGCGGAGGCAAA	KGGGK
120EBF1301219	GER-CW		0	Hullersen	51.81095	9.80442	accurate	*Lepus europaeus*	NA	0	0	1	NA	0	0	1	NA	NA	NA	NA	NA	NA	NA	NA	NA
121EBF1301219	GER-CW		0	Hullersen	51.81095	9.80442	accurate	*Lepus europaeus*	NA	0	1	0	NA	0	1	0	NA	NA	NA	NA	NA	NA	NA	NA	NA
122EBF1301219	GER-CW		0	Hullersen	51.81095	9.80442	accurate	*Lepus europaeus*	NA	0	1	0	NA	0	1	0	NA	NA	NA	NA	NA	NA	NA	NA	NA
123EBM1301219	GER-CW		0	Hullersen	51.81095	9.80442	accurate	*Lepus europaeus*	NA	0	0	1	NA	0	0	1	NA	NA	NA	NA	NA	NA	NA	NA	NA
124EBF1301219	GER-CW		0	Hullersen	51.81095	9.80442	accurate	*Lepus europaeus*	NA	0	0	1	NA	0	0	1	NA	NA	NA	NA	NA	NA	NA	NA	NA
125EBF1301219	GER-CW		0	Hullersen	51.81095	9.80442	accurate	*Lepus europaeus*	NA	0	0	1	NA	0	0	1	NA	NA	NA	NA	NA	NA	NA	NA	NA
126EBM1301219	GER-CW		0	Hullersen	51.81095	9.80442	accurate	*Lepus europaeus*	NA	0	0	1	NA	0	0	1	NA	NA	NA	NA	NA	NA	NA	NA	NA
127EBF1301219	GER-CW		0	Hullersen	51.81095	9.80442	accurate	*Lepus europaeus*	NA	0	0	1	NA	0	0	1	NA	NA	NA	NA	NA	NA	NA	NA	NA
128EBF1301219	GER-CW		0	Hullersen	51.81095	9.80442	accurate	*Lepus europaeus*	NA	0	0	1	NA	0	0	1	NA	NA	NA	NA	NA	NA	NA	NA	NA
129EBF1301219	GER-CW		0	Hullersen	51.81095	9.80442	accurate	*Lepus europaeus*	NA	0	0	1	NA	0	0	1	NA	NA	NA	NA	NA	NA	NA	NA	NA
130EBF1301219	GER-CW		0	Hullersen	51.81095	9.80442	accurate	*Lepus europaeus*	NA	0	0	1	NA	0	0	1	NA	NA	NA	NA	NA	NA	NA	NA	NA
131EBM1301219	GER-CW		0	Hullersen	51.81095	9.80442	accurate	*Lepus europaeus*	NA	0	0	1	NA	0	0	1	NA	NA	NA	NA	NA	NA	NA	NA	NA
132EBF1301219	GER-CW		0	Hullersen	51.81095	9.80442	accurate	*Lepus europaeus*	NA	0	0	1	NA	0	0	1	NA	NA	NA	NA	NA	NA	NA	NA	NA
133EBM2301219	GER-CW		0	Hullersen	51.81095	9.80442	accurate	*Lepus europaeus*	NA	0	1	0	NA	0	1	0	NA	NA	NA	NA	NA	NA	NA	NA	NA
134EBF1301219	GER-CW		0	Hullersen	51.81095	9.80442	accurate	*Lepus europaeus*	NA	0	0	1	NA	0	0	1	NA	NA	NA	NA	NA	NA	NA	NA	NA
135EBM1301219	GER-CW		0	Hullersen	51.81095	9.80442	accurate	*Lepus europaeus*	NA	0	0	1	NA	0	0	1	NA	NA	NA	NA	NA	NA	NA	NA	NA
136EBM1301219	GER-CW		0	Hullersen	51.81095	9.80442	accurate	*Lepus europaeus*	NA	1	0	0	OM939694	1	0	0	OM991042	NA	NA	AAAGGC	KG	NA	3	AAAGGCGGAGGCAAA	KGGGK
137EBM1301219	GER-CW		0	Hullersen	51.81095	9.80442	accurate	*Lepus europaeus*	NA	1	0	0	OM939693	1	0	0	**OM991062**	GGAGGC	GG	AAAGGTGGAGGC	KGGG	NA	1	AGGTAGAGGCAAAGGTGGAGGCAAAGGCA	KGRGKGGGKGK
138EBM1301219	GER-CW		0	Hullersen	51.81095	9.80442	accurate	*Lepus europaeus*	NA	0	1	0	NA	0	1	0	NA	NA	NA	NA	NA	NA	NA	NA	NA
139EBM1301219	GER-CW		0	Hullersen	51.81095	9.80442	accurate	*Lepus europaeus*	NA	0	1	0	NA	0	1	0	NA	NA	NA	NA	NA	NA	NA	NA	NA
140EBM1301219	GER-CW		0	Hullersen	51.81095	9.80442	accurate	*Lepus europaeus*	NA	0	1	0	NA	0	0	1	NA	NA	NA	NA	NA	NA	NA	NA	NA
141EBM1301219	GER-CW		0	Hullersen	51.81095	9.80442	accurate	*Lepus europaeus*	NA	0	1	0	NA	0	1	0	NA	NA	NA	NA	NA	NA	NA	NA	NA
142EBM1301219	GER-CW		0	Hullersen	51.81095	9.80442	accurate	*Lepus europaeus*	NA	1	0	0	OM939693	1	0	0	**OM991063**	GGAGGC	GG	AAAGGTGGAGGC	KGGG	NA	1	AAAGGTGGAGACAAAGGTGGAGGCAAA	KGGDKGGGK
143EBF1301219	GER-CW		0	Hullersen	51.81095	9.80442	accurate	*Lepus europaeus*	NA	0	0	1	NA	0	0	1	NA	NA	NA	NA	NA	NA	NA	NA	NA
01BYMX151019	GER-S		unknown	Brackenlohr-Aspachhof	49.55437	10.17586	estimated	*Lepus europaeus*	**ON089363**	1	0	0	OM939696	1	0	0	**OM990856**	GGAGGC	GG	AAAGGTGGAGGC	KGGG	I	3	AAAGGCAAA	KGK
02BYFX151019	GER-S		unknown	Heuberg bei Hilpoltstein	49.18896	11.18922	estimated	*Lepus europaeus*	NA	0	0	1	NA	0	0	1	NA	NA	NA	NA	NA	NA	NA	NA	NA
03BYFX151019	GER-S		unknown	Thundorf	50.20084	10.31887	estimated	*Lepus europaeus*	NA	0	0	1	NA	0	0	1	NA	NA	NA	NA	NA	NA	NA	NA	NA
04BYMX151019	GER-S		unknown	Leutersheim	49.29928	10.41177	estimated	*Lepus europaeus*	NA	0	0	1	NA	0	0	1	NA	NA	NA	NA	NA	NA	NA	NA	NA
05BYFX151019	GER-S		unknown	Eichenhubl	50.19840	10.07510	estimated	*Lepus europaeus*	NA	0	0	1	NA	0	0	1	NA	NA	NA	NA	NA	NA	NA	NA	NA
06BYMX151019	GER-S		unknown	Uffenheim	49.54400	10.23437	estimated	*Lepus europaeus*	NA	0	0	1	NA	0	0	1	NA	NA	NA	NA	NA	NA	NA	NA	NA
07BYMX151019	GER-S		unknown	Hambach	50.09473	10.20797	estimated	*Lepus europaeus*	NA	0	0	1	NA	0	0	1	NA	NA	NA	NA	NA	NA	NA	NA	NA
08BYFX151019	GER-S		unknown	Hambach	50.09473	10.20797	estimated	*Lepus europaeus*	NA	0	0	1	NA	0	0	1	NA	NA	NA	NA	NA	NA	NA	NA	NA
09BYMX151019	GER-S		unknown	Ramsthal	50.13972	10.06906	estimated	*Lepus europaeus*	NA	0	0	1	NA	0	0	1	NA	NA	NA	NA	NA	NA	NA	NA	NA
10BYMX151019	GER-S		unknown	Herreth	50.12689	10.93376	estimated	*Lepus europaeus*	NA	0	0	1	NA	0	0	1	NA	NA	NA	NA	NA	NA	NA	NA	NA
11BYMX151019	GER-S		unknown	Neustadt bei Coburg	50.33181	11.11738	estimated	*Lepus europaeus*	NA	0	0	1	NA	0	0	1	NA	NA	NA	NA	NA	NA	NA	NA	NA
12BYMX151019	GER-S		unknown	Coburg	50.25811	10.96446	estimated	*Lepus europaeus*	NA	0	0	1	NA	0	0	1	NA	NA	NA	NA	NA	NA	NA	NA	NA
13BYFX151019	GER-S		unknown	Itzgrund	50.15000	10.90000	estimated	*Lepus europaeus*	**ON089402**	0	0	1	NA	1	0	0	**OM990894**	GGAGGC	GG	AAAGGTGGAGGC	KGGG	I	3	AAA	K
14BYMX151019	GER-S		unknown	Haarth-Triebsdorf	50.22588	10.97155	estimated	*Lepus europaeus*	NA	0	0	1	NA	0	0	1	NA	NA	NA	NA	NA	NA	NA	NA	NA
15BYFX151019	GER-S		unknown	Ohrenbach	49.46969	10.21036	estimated	*Lepus europaeus*	NA	0	0	1	NA	0	0	1	NA	NA	NA	NA	NA	NA	NA	NA	NA
16BYMX151019	GER-S		unknown	Ohrenbach	49.46969	10.21036	estimated	*Lepus europaeus*	**ON089416**	1	0	0	OM939693	1	0	0	**OM990908**	AGAGGCAAAGGTAGAGGC	RGKGRG	AAAGGTGGAGGC	KGGG	NA	2	AAA	K
17BYFX151019	GER-S		unknown	Herrnsdorf	49.81717	10.86424	estimated	*Lepus europaeus*	NA	0	0	1	NA	0	0	1	NA	NA	NA	NA	NA	NA	NA	NA	NA
18BYFX151019	GER-S		unknown	Tiefenpölz	49.88934	11.11219	estimated	*Lepus europaeus*	NA	0	0	1	NA	0	0	1	NA	NA	NA	NA	NA	NA	NA	NA	NA
19BYFX151019	GER-S		unknown	Weidach	50.25524	10.87941	estimated	*Lepus europaeus*	NA	0	0	1	NA	0	0	1	NA	NA	NA	NA	NA	NA	NA	NA	NA
20BYFX151019	GER-S		unknown	Fürth	49.47726	10.98962	estimated	*Lepus europaeus*	NA	0	0	1	NA	0	0	1	NA	NA	NA	NA	NA	NA	NA	NA	NA
21BYM1281219	GER-S		0	Großeibstadt	50.31070	10.43243	accurate	*Lepus europaeus*	NA	0	0	1	NA	0	0	1	NA	NA	NA	NA	NA	NA	NA	NA	NA
22BYM1281219	GER-S		0	Großeibstadt	50.31070	10.43243	accurate	*Lepus europaeus*	NA	0	0	1	NA	0	0	1	NA	NA	NA	NA	NA	NA	NA	NA	NA
23BYF1281219	GER-S		0	Großeibstadt	50.31070	10.43243	accurate	*Lepus europaeus*	NA	0	0	1	NA	0	0	1	NA	NA	NA	NA	NA	NA	NA	NA	NA
24BYF1281219	GER-S		0	Großeibstadt	50.31070	10.43243	accurate	*Lepus europaeus*	NA	0	0	1	NA	0	0	1	NA	NA	NA	NA	NA	NA	NA	NA	NA
25BYF1281219	GER-S		0	Großeibstadt	50.31070	10.43243	accurate	*Lepus europaeus*	NA	0	0	1	NA	0	0	1	NA	NA	NA	NA	NA	NA	NA	NA	NA
26BYM1281219	GER-S		0	Großeibstadt	50.31070	10.43243	accurate	*Lepus europaeus*	**ON089440**	1	0	0	OM939694	1	0	0	**OM990934**	GGAGGC	GG	AAAGGTGGAGGC	KGGG	I	2	AAA	K
27BYM1281219	GER-S		0	Großeibstadt	50.31070	10.43243	accurate	*Lepus europaeus*	NA	0	0	1	NA	0	0	1	NA	NA	NA	NA	NA	NA	NA	NA	NA
28BYM1281219	GER-S		0	Großeibstadt	50.31070	10.43243	accurate	*Lepus europaeus*	NA	0	0	1	NA	0	0	1	NA	NA	NA	NA	NA	NA	NA	NA	NA
29BYF1281219	GER-S		0	Großeibstadt	50.31070	10.43243	accurate	*Lepus europaeus*	NA	0	1	0	NA	0	0	1	NA	NA	NA	NA	NA	NA	NA	NA	NA
30BYF1281219	GER-S		0	Großeibstadt	50.31070	10.43243	accurate	*Lepus europaeus*	NA	0	0	1	NA	0	0	1	NA	NA	NA	NA	NA	NA	NA	NA	NA
31BYM1281219	GER-S		0	Großeibstadt	50.31070	10.43243	accurate	*Lepus europaeus*	NA	0	0	1	NA	0	0	1	NA	NA	NA	NA	NA	NA	NA	NA	NA

32BYF1281219	GER-S	[redacted]	0	Großeibstadt	50.31070	10.43243	accurate	*Lepus europaeus*	NA	0	1	0	NA	0	1	0	NA	NA	NA	NA	NA	NA	NA	NA	NA
33BYM2281219	GER-S	[redacted]	1	Großeibstadt	50.31070	10.43243	accurate	*Lepus europaeus*	ON089569	0	0	1	NA	1	0	0	**OM990944**	GGAGGC	GG	AAAGGTAAAGGC	KGKG	II	2	AAAGGCAAA	KGK
34BYM1281219	GER-S	[redacted]	0	Großeibstadt	50.31070	10.43243	accurate	*Lepus europaeus*	NA	0	0	1	NA	0	0	1	NA	NA	NA	NA	NA	NA	NA	NA	NA
35BYM1281219	GER-S	[redacted]	0	Großeibstadt	50.31070	10.43243	accurate	*Lepus europaeus*	ON089569	1	0	0	OM939693	1	0	0	**OM990947**	GTAGGC	VG	AAAGGTGGAGGC	KGGG	I	5	AAA	K
36BYM1281219	GER-S	[redacted]	0	Großeibstadt	50.31070	10.43243	accurate	*Lepus europaeus*	NA	0	0	1	NA	0	1	0	NA	NA	NA	NA	NA	NA	NA	NA	NA
37BYF1281219	GER-S	[redacted]	0	Großeibstadt	50.31070	10.43243	accurate	*Lepus europaeus*	NA	0	0	1	NA	0	0	1	NA	NA	NA	NA	NA	NA	NA	NA	NA
38BYF1281219	GER-S	[redacted]	0	Großeibstadt	50.31070	10.43243	accurate	*Lepus europaeus*	NA	0	0	1	NA	0	0	1	NA	NA	NA	NA	NA	NA	NA	NA	NA
39BYFX180220	GER-S	[redacted]	unknown	Günzburg	49.16371	9.43875	estimated	*Lepus europaeus*	**ON089444**	0	1	0	NA	0	0	1	NA	NA	NA	NA	NA	NA	NA	NA	NA
40BYMX180220	GER-S	[redacted]	unknown	Altötting	48.41592	9.91876	estimated	*Lepus europaeus*	**ON089445**	0	1	0	NA	0	0	1	NA	NA	NA	NA	NA	NA	NA	NA	NA
41BYFX180220	GER-S	[redacted]	unknown	Fürstenfeldbruck	48.17049	11.35264	estimated	*Lepus europaeus*	**ON089447**	0	0	1	NA	0	0	1	NA	NA	NA	NA	NA	NA	NA	NA	NA
42BYMX180220	GER-S	[redacted]	unknown	Straubing	48.83078	12.54812	estimated	*Lepus europaeus*	**ON089448**	1	0	0	OM939694	1	0	0	**OM990957**	GGAAGC	GS	AAAGGTGGAGGC	KGGG	I	1	AAA	K
43BYFX180220	GER-S	[redacted]	unknown	Dachau	48.36077	11.37430	estimated	*Lepus europaeus*	NA	0	0	1	NA	0	0	1	NA	NA	NA	NA	NA	NA	NA	NA	NA
44BYMX180220	GER-S	[redacted]	unknown	Freising	48.46852	11.80994	estimated	*Lepus europaeus*	NA	0	0	1	NA	0	0	1	NA	NA	NA	NA	NA	NA	NA	NA	NA
45BYMX180220	GER-S	[redacted]	unknown	Pfaffenhofen a.d. Ilm	48.53149	11.50543	estimated	*Lepus europaeus*	**ON089452**	1	0	0	OM939693	1	0	0	**OM990962**	GGAGGC	GG	AAAGGTGGAGGC	KGGG	I	2	AAA	K
46BYMX281019	GER-S	[redacted]	unknown	Freising	48.46670	11.50816	estimated	*Lepus europaeus*	NA	0	0	1	NA	0	0	1	NA	NA	NA	NA	NA	NA	NA	NA	NA
47BYMX311019	GER-S	[redacted]	unknown	Rosenheim	48.02688	12.09234	estimated	*Lepus europaeus*	NA	0	0	1	NA	0	0	1	NA	NA	NA	NA	NA	NA	NA	NA	NA
48BYXX041119	GER-S	[redacted]	unknown	Pfaffenhofen a.d. Ilm	48.53149	11.50543	estimated	*Lepus europaeus*	NA	0	0	1	NA	0	0	1	NA	NA	NA	NA	NA	NA	NA	NA	NA
49BYFX051119	GER-S	[redacted]	unknown	Freising	48.30000	11.61670	estimated	*Lepus europaeus*	NA	0	0	1	NA	0	0	1	NA	NA	NA	NA	NA	NA	NA	NA	NA
50BYFX051119	GER-S	[redacted]	unknown	Dingolfing	48.63008	12.49774	estimated	*Oryctolagus cuniculus*	**ON089458**	0	0	1	NA	0	0	1	NA	NA	NA	NA	NA	NA	NA	NA	NA
51BYFX051119	GER-S	[redacted]	unknown	Dingolfing	48.63008	12.49774	estimated	*Oryctolagus cuniculus*	ON089430	0	0	1	NA	0	0	1	NA	NA	NA	NA	NA	NA	NA	NA	NA
52BYXX061119	GER-S	[redacted]	unknown	Aichach-Friedberg	48.46115	11.11267	estimated	*Lepus europaeus*	NA	0	0	1	NA	0	0	1	NA	NA	NA	NA	NA	NA	NA	NA	NA
53BYFX061119	GER-S	[redacted]	unknown	Aichach-Friedberg	48.38819	11.08933	estimated	*Lepus europaeus*	NA	0	0	1	NA	0	0	1	NA	NA	NA	NA	NA	NA	NA	NA	NA
54BYMX061119	GER-S	[redacted]	unknown	Landsberg am Lech	48.24490	10.98638	estimated	*Lepus europaeus*	NA	0	0	1	NA	0	1	0	NA	NA	NA	NA	NA	NA	NA	NA	NA
55BYMX061119	GER-S	[redacted]	unknown	Neu-Ulm	48.31641	10.06886	estimated	*Lepus europaeus*	**ON089463**	0	0	1	NA	1	0	0	**OM990973**	NA	NA	AAAGGCCAAGGT	KGQG	NA	2	AAAGGCAAA	KGK
56BYFX061119	GER-S	[redacted]	unknown	Bayreuth	49.93969	11.72245	estimated	*Lepus europaeus*	NA	0	0	1	NA	0	0	1	NA	NA	NA	NA	NA	NA	NA	NA	NA
57BYMX071119	GER-S	[redacted]	unknown	Dingolfing	48.73481	12.75064	estimated	*Lepus europaeus*	NA	0	0	1	NA	0	0	1	NA	NA	NA	NA	NA	NA	NA	NA	NA
58BYMX071119	GER-S	[redacted]	unknown	München	48.13667	11.40323	estimated	*Lepus europaeus*	ON089569	0	1	0	NA	0	1	0	NA	NA	NA	NA	NA	NA	NA	NA	NA
59BYFX071119	GER-S	[redacted]	unknown	Neuburg a.d. Donau	48.73720	11.17953	estimated	*Lepus europaeus*	**ON089468**	0	1	0	NA	0	0	1	NA	NA	NA	NA	NA	NA	NA	NA	NA
60BYFX071119	GER-S	[redacted]	unknown	Fürstenfeldbruck	48.17049	11.35264	estimated	*Lepus europaeus*	NA	0	0	1	NA	0	0	1	NA	NA	NA	NA	NA	NA	NA	NA	NA
61BYUX121119	GER-S	[redacted]	unknown	Freising	48.40083	11.74396	estimated	*Lepus europaeus*	NA	0	0	1	NA	0	0	1	NA	NA	NA	NA	NA	NA	NA	NA	NA
62BYMX121119	GER-S	[redacted]	unknown	Fürstenfeldbruck	48.25517	11.09523	estimated	*Lepus europaeus*	**ON089473**	0	0	1	NA	0	0	1	NA	NA	NA	NA	NA	NA	NA	NA	NA
63BYMX121119	GER-S	[redacted]	unknown	Donauwörth	48.66303	10.82585	estimated	*Oryctolagus cuniculus*	**ON089474**	0	0	1	NA	0	0	1	NA	NA	NA	NA	NA	NA	NA	NA	NA
64BYFX131119	GER-S	[redacted]	unknown	Eichstätt	48.94921	11.39486	estimated	*Lepus europaeus*	ON089569	1	0	0	OM939694	1	0	0	**OM990984**	GGAGGC	GG	AAAGGTGGAGGC	KGGG	I	3	AAA	K
65BYFX131119	GER-S	[redacted]	unknown	Eichstätt	48.81932	11.31970	estimated	*Lepus europaeus*	NA	0	0	1	NA	0	0	1	NA	NA	NA	NA	NA	NA	NA	NA	NA
66BYFX141119	GER-S	[redacted]	unknown	Donauwörth	48.68238	10.81359	estimated	*Oryctolagus cuniculus f. domestica*	**ON089481**	0	0	1	NA	0	0	1	NA	NA	NA	NA	NA	NA	NA	NA	NA
67BYFX141119	GER-S	[redacted]	unknown	Donauwörth	48.68238	10.81359	estimated	*Oryctolagus cuniculus f. domestica*	**ON089483**	0	0	1	NA	0	0	1	NA	NA	NA	NA	NA	NA	NA	NA	NA
68BYFX271119	GER-S	[redacted]	unknown	Traunstein	47.93410	12.73261	estimated	*Lepus europaeus*	**ON089485**	1	0	0	OM939693	1	0	0	**OM990995**	GGAGTC	GV	AAAGGTAAAGGC	KGKG	NA	1	(AAAGGT)2AAAGGCAAA	(KG)2KGK
69BYMX271119	GER-S	[redacted]	unknown	Landsberg am Lech	48.00909	10.97147	estimated	*Lepus europaeus*	**ON089487**	0	1	0	NA	0	1	0	NA	NA	NA	NA	NA	NA	NA	NA	NA
70BYMX271119	GER-S	[redacted]	unknown	Landsberg am Lech	48.05208	10.87126	estimated	*Lepus europaeus*	**ON089489**	1	0	0	OM939693	1	0	0	**OM990996**	GGAGGC	GG	AAAGGTGGAGGC	KGGG	I	2	AAA	K
71BYFX281119	GER-S	[redacted]	unknown	Eichstätt	48.80430	11.05686	estimated	*Lepus europaeus*	NA	0	0	1	NA	0	0	1	NA	NA	NA	NA	NA	NA	NA	NA	NA
72BYFX291119	GER-S	[redacted]	unknown	Fürstenfeldbruck	48.21527	11.21822	estimated	*Lepus europaeus*	NA	0	0	1	NA	0	0	1	NA	NA	NA	NA	NA	NA	NA	NA	NA
73BYFX291119	GER-S	[redacted]	unknown	Fürstenfeldbruck	48.20531	11.33805	estimated	*Lepus europaeus*	NA	0	0	1	NA	0	0	1	NA	NA	NA	NA	NA	NA	NA	NA	NA
74BYFX031219	GER-S	[redacted]	unknown	Aichach	48.26804	11.04612	estimated	*Lepus europaeus*	**ON089493**	0	0	1	NA	1	0	0	**OM990999**	GGAGTC	GV	AAAGGTAAAGGC	KGKG	NA	1	(AAAGGT)2AAAGGCAAA	(KG)2KGK
75BYMX051219	GER-S	[redacted]	unknown	Dillingen a.d. Donau	48.53020	10.69599	estimated	*Lepus europaeus*	NA	0	0	1	NA	0	0	1	NA	NA	NA	NA	NA	NA	NA	NA	NA
76BYFX061219	GER-S	[redacted]	unknown	Freyung-Grafenau	48.73002	13.60158	estimated	*Lepus europaeus*	**ON089497**	0	1	0	NA	0	1	0	NA	NA	NA	NA	NA	NA	NA	NA	NA
77BYFX061219	GER-S	[redacted]	unknown	Freyung-Grafenau	48.81543	13.55591	estimated	*Lepus europaeus*	NA	0	0	1	NA	0	0	1	NA	NA	NA	NA	NA	NA	NA	NA	NA
78BYFX101219	GER-S	[redacted]	unknown	Erding	48.32241	11.84644	estimated	*Lepus europaeus*	**ON089501**	0	1	0	NA	0	1	0	NA	NA	NA	NA	NA	NA	NA	NA	NA
79BYMX101219	GER-S	[redacted]	unknown	Neuburg a.d. Donau	48.68227	11.28775	estimated	*Lepus europaeus*	**ON089504**	0	1	0	NA	0	1	0	NA	NA	NA	NA	NA	NA	NA	NA	NA
80BYMX111219	GER-S	[redacted]	unknown	Landshut	48.53622	12.15166	estimated	*Oryctolagus cuniculus*	**ON089507**	0	0	1	NA	0	0	1	NA	NA	NA	NA	NA	NA	NA	NA	NA
81BYMX111219	GER-S	[redacted]	unknown	Pfaffenhofen a.d. Ilm	48.69423	11.63565	estimated	*Lepus europaeus*	NA	0	0	1	NA	0	0	1	NA	NA	NA	NA	NA	NA	NA	NA	NA
82BYFX161219	GER-S	[redacted]	unknown	Eichstätt	48.84945	11.64416	estimated	*Lepus europaeus*	**ON089509**	0	1	0	NA	0	1	0	NA	NA	NA	NA	NA	NA	NA	NA	NA
83BYMX171219	GER-S	[redacted]	unknown	Straubing	48.88198	12.56972	estimated	*Lepus europaeus*	NA	0	0	1	NA	0	0	1	NA	NA	NA	NA	NA	NA	NA	NA	NA
84BYMX171219	GER-S	[redacted]	unknown	Aichach	48.24490	10.98638	estimated	*Lepus europaeus*	NA	0	0	1	NA	0	0	1	NA	NA	NA	NA	NA	NA	NA	NA	NA
85BYFX191219	GER-S	[redacted]	unknown	Rosenheim	47.82943	12.12684	estimated	*Lepus europaeus*	**ON089513**	0	1	0	NA	1	0	0	**OM991011**	GGAGGC	GG	AAAGGTGGAGGC	KGGG	I	4	AAA	K
86BYMX191219	GER-S	[redacted]	unknown	Erding	48.27782	11.84509	estimated	*Lepus europaeus*	NA	0	0	1	NA	0	0	1	NA	NA	NA	NA	NA	NA	NA	NA	NA
87BYMX191219	GER-S	[redacted]	unknown	Augsburg	48.29006	10.65647	estimated	*Lepus europaeus*	NA	0	0	1	NA	0	0	1	NA	NA	NA	NA	NA	NA	NA	NA	NA
88BYFX231219	GER-S	[redacted]	unknown	Fürstenfeldbruck	48.20531	11.33805	estimated	*Lepus europaeus*	**ON089514**	0	1	0	NA	0	0	1	NA	NA	NA	NA	NA	NA	NA	NA	NA
89BYFX030120	GER-S	[redacted]	unknown	Erding	48.25892	11.97252	estimated	*Lepus europaeus*	**ON089516**	1	0	0	OM939693	1	0	0	**OM991013**	NA	NA	AAAGGCAAAGGT	KGKG	NA	1	AAAGGCAAAGGTGGAGGCAAA	KGKGGGK
90BYMX080120	GER-S	[redacted]	unknown	Freising	48.35042	11.56159	estimated	*Lepus europaeus*	**ON089515**	0	1	0	NA	0	0	1	NA	NA	NA	NA	NA	NA	NA	NA	NA
91BYFX090120	GER-S	[redacted]	unknown	Deggendorf	48.76168	12.80962	estimated	*Lepus europaeus*	NA	0	0	1	NA	0	0	1	NA	NA	NA	NA	NA	NA	NA	NA	NA
92BYMX130120	GER-S	[redacted]	unknown	Landsberg am Lech	48.05208	10.87126	estimated	*Lepus europaeus*	ON089569	0	0	1	NA	1	0	0	**OM991015**	GTAGGC	VG	AAAGGTGGAGGC	KGGG	I	2	AAA	K
93BYFX130120	GER-S	[redacted]	unknown	Landsberg am Lech	48.21965	10.84108	estimated	*Lepus europaeus*	**ON089517**	0	1	0	NA	0	0	1	NA	NA	NA	NA	NA	NA	NA	NA	NA
94BYMX140120	GER-S	[redacted]	unknown	Freising	48.46670	11.50816	estimated	*Lepus europaeus*	NA	0	0	1	NA	0	0	1	NA	NA	NA	NA	NA	NA	NA	NA	NA
95BYMX160120	GER-S	[redacted]	unknown	Günzburg	48.45390	10.27855	estimated	*Lepus europaeus*	NA	0	0	1	NA	0	0	1	NA	NA	NA	NA	NA	NA	NA	NA	NA
96BYMX160120	GER-S	[redacted]	unknown	Freyung-Grafenau	48.85764	13.39383	estimated	*Lepus europaeus*	**ON089521**	1	0	0	OM939693	1	0	0	**OM991020**	GGAGGC	GG	AAAGGTGGAGGC	KGGG	I	5	AAA	K
97BYFX160120	GER-S	[redacted]	unknown	Freyung-Grafenau	48.85764	13.39383	estimated	*Lepus europaeus*	**ON089522**	0	0	1	NA	1	0	0	**OM991021**	GGAGGC	GG	AAAGGTGGAGGC	KGGG	I	2	AAA	K
98BYMX170120	GER-S	[redacted]	unknown	Landshut	48.53622	12.15166	estimated	*Oryctolagus cuniculus*	**ON089524**	0	0	1	NA	1	0	0	**OM991024**	GGAGGC	GG	AAAGGTGGAGGC	KGGG	I	5	AAA	K
99BYFX170120	GER-S	[redacted]	unknown	Landshut	48.53622	12.15166	estimated	*Oryctolagus cuniculus*	**ON089526**	0	0	1	NA	0	0	1	NA	NA	NA	NA	NA	NA	NA	NA	NA
100BYMX240120	GER-S	[redacted]	unknown	Deggendorf	48.91524	12.81327	estimated	*Lepus europaeus*	NA	0	0	1	NA	0	0	1	NA	NA	NA	NA	NA	NA	NA	NA	NA
101BYFX240120	GER-S	[redacted]	unknown	Deggendorf	48.84094	12.96878	estimated	*Lepus europaeus*	NA	0	0	1	NA	0	0	1	NA	NA	NA	NA	NA	NA	NA	NA	NA
102BYFX171019	GER-S	[redacted]	unknown	Niederhof	49.31300	12.14125	estimated	*Lepus europaeus*	**ON089527**	1	0	0	OM939693	1	0	0	**OM991027**	GTAGGC	VG	AAAGGTGGAGGC	KGGG	I	3	AAA	K
103BYMX171019	GER-S	[redacted]	unknown	Guttenberg	50.15499	11.56462	estimated	*Lepus europaeus*	NA	0	0	1	NA	0	0	1	NA	NA	NA	NA	NA	NA	NA	NA	NA
104BYFX171019	GER-S	[redacted]	unknown	Eckersdorf	49.93375	11.50087	estimated	*Lepus europaeus*	**ON089529**	0	1	0	NA	0	0	1	NA	NA	NA	NA	NA	NA	NA	NA	NA
105BYFX171019	GER-S	[redacted]	unknown	Eckersdorf	49.93375	11.50087	estimated	*Lepus europaeus*	NA	0	0	1	NA	0	0	1	NA	NA	NA	NA	NA	NA	NA	NA	NA
106BYFX181019	GER-S	[redacted]	unknown	Creussen	49.84385	11.62528	estimated	*Lepus europaeus*	**ON089531**	1	0	0	OM939693	1	0	0	**OM991030**	GGAGGC	GG	AAAGGTGGAGGC	KGGG	I	3	AAA	K
107BYFX181019	GER-S	[redacted]	unknown	Würzburg	49.79245	9.93297	estimated	*Lepus europaeus*	NA	0	0	1	NA	0	0	1	NA	NA	NA	NA	NA	NA	NA	NA	NA
108BYFX211019	GER-S	[redacted]	unknown	Baiersdorf	49.65709	11.03386	estimated	*Lepus europaeus*	NA	0	0	1	NA	0	0	1	NA	NA	NA	NA	NA	NA	NA	NA	NA
109BYFX221019	GER-S	[redacted]	unknown	Hohholz	49.59274	10.71624	estimated	*Lepus europaeus*	NA	0	0	1	NA	0	0	1	NA	NA	NA	NA	NA	NA	NA	NA	NA
110BYFX241019	GER-S	[redacted]	unknown	Birkensee bei Kitzingen	49.71799	10.27842	estimated	*Lepus europaeus*	**ON089536**	0	0	1	NA	1	0	0	OM991030	GGAGGC	GG	AAAGGTGGAGGC	KGGG	I	3	AAA	K
111BYFX241019	GER-S	[redacted]	unknown	Rödelsee bei Kitzingen	49.72839	10.24637	estimated	*Lepus europaeus*	NA	0	0	1	NA	0	0	1	NA	NA	NA	NA	NA	NA	NA	NA	NA
112BYFX241019	GER-S	[redacted]	unknown	Exerzierplatz Erlangen	49.58503	11.02781	estimated	*Oryctolagus cuniculus*	**ON089538**	0	0	1	NA	0	0	1	NA	NA	NA	NA	NA	NA	NA	NA	NA
113BYMX241019	GER-S	[redacted]	unknown	Fladungen	50.52104	10.14585	estimated	*Lepus europaeus*	NA	0	0	1	NA	0	0	1	NA	NA	NA	NA	NA	NA	NA	NA	NA
114BYFX281019	GER-S	[redacted]	unknown	Rauschensteig Wunsiedel	50.07835	12.01612	estimated	*Lepus europaeus*	NA	0	1	0	NA	0	0	1	NA	NA	NA	NA	NA	NA	NA	NA	NA
115BYFX250220	GER-S	[redacted]	unknown	Kitzingen	49.73398	10.16122	estimated	*Lepus europaeus*	NA	0	0	1	NA	0	0	1	NA	NA	NA	NA	NA	NA	NA	NA	NA
116BYFX291019	GER-S	[redacted]	unknown	Rednitzhembach	49.30149	11.07830	estimated	*Lepus europaeus*	**ON089540**	0	1	0	NA	0	0	1	NA	NA	NA	NA	NA	NA	NA	NA	NA
117BYFX311019	GER-S	[redacted]	unknown	Schwandorf	49.32619	12.10927	estimated	*Lepus europaeus*	**ON089542**	0	1	0	NA	0	0	1	NA	NA	NA	NA	NA	NA	NA	NA	NA
118BYFX041119	GER-S	[redacted]	unknown	Herreth	50.12689	10.93376	estimated	*Lepus europaeus*	**ON089543**	0	1	0	NA	0	0	1	NA	NA	NA	NA	NA	NA	NA	NA	NA
119BYFX041119	GER-S	[redacted]	unknown	Lichtenau	49.28051	10.68364	estimated	*Lepus europaeus*	**ON089544**	0	1	0	NA	0	0	1	NA	NA	NA	NA	NA	NA	NA	NA	NA
120BYFX031119	GER-S	[redacted]	unknown	Offenhausen	49.44951	11.41406	estimated	*Lepus europaeus*	**ON089546**	0	1	0	NA	0	0	1	NA	NA	NA	NA	NA	NA	NA	NA	NA
121BYMX021119	GER-S	[redacted]	unknown	Sulzbach-Rosenberg	49.49925	11.74703	estimated	*Lepus europaeus*	**ON089547**	0	1	0	NA	0	1	0	NA	NA	NA	NA	NA	NA	NA	NA	NA
122BYFX061119	GER-S	[redacted]	unknown	Adlholz	49.58019	11.83886	estimated	*Lepus europaeus*	**ON089548**	0	0	1	NA	1	0	0	**OM991044**	GTAGGC(AAAGGTGTAGGC)2	VG(KGVG)2	AAAGGTGGAGGC	KGGG	NA	1	AAA	K
123BYFX061119	GER-S	[redacted]	unknown	Creidlitz	50.23402	10.98068	estimated	*Lepus europaeus*	NA	0	0	1	NA	0	0	1	NA	NA	NA	NA	NA	NA	NA	NA	NA
124BYFX071119	GER-S	[redacted]	unknown	Cham	49.21782	12.66638	estimated	*Lepus europaeus*	NA	0	0	1	NA	0	0	1	NA	NA	NA	NA	NA	NA	NA	NA	NA
125BYFX071119	GER-S	[redacted]	unknown	Pempfling	49.26534	12.61139	estimated	*Lepus europaeus*	**ON089550**	0	0	1	NA	1	0	0	OM990885	GGAGGC	GG	AAAGGTGGAGGC	KGGG	I	3	AAA	K
126BYFX071119	GER-S	[redacted]	unknown	Alpenberg	49.24197	10.96387	estimated	*Lepus europaeus*	NA	0	0	1	NA	0	0	1	NA	NA	NA	NA	NA	NA	NA	NA	NA
127BYFX081119	GER-S	[redacted]	unknown	Bubach	49.26165	12.07817	estimated	*Lepus europaeus*	**ON089552**	0	1	0	NA	1	0	0	**OM991051**	GGAGGC	GG	AAAGGTGGAGGC	KGGG	I	4	AAA	K
128BYFX241019	GER-S	[redacted]	unknown	Friedenfels	49.88285	12.10160	estimated	*Lepus europaeus*	NA	0	0	1	NA	0	0	1	NA	NA	NA	NA	NA	NA	NA	NA	NA
129BYXX250220	GER-S	[redacted]	unknown	Holzberghof	50.44063	10.00840	estimated	*Lepus europaeus*	NA	0	0	1	NA	0	0	1	NA	NA	NA	NA	NA	NA	NA	NA	NA
130BYFX031219	GER-S	[redacted]	unknown	Fürth	49.47726	10.98962	estimated	*Lepus europaeus*	NA	0	0	1	NA	0	0	1	NA	NA	NA	NA	NA	NA	NA	NA	NA
131BYMX121219	GER-S	[redacted]	unknown	Hohentau	49.87803	12.33720	estimated	*Lepus europaeus*	NA	0	0	1	NA	0	0	1	NA	NA	NA	NA	NA	NA	NA	NA	NA
132BYMX030120	GER-S	[redacted]	unknown	Buchenbach bei Erlangen	49.59197	10.96242	estimated	*Lepus europaeus*	**ON089556**	1	0	0	OM939693	1	0	0	**OM991057**	GGAGGC	GG	AAAGGTGGAGGC	KGGG	I	3	AAA	K
133BYMX030120	GER-S	[redacted]	unknown	Traitsching	49.15361	12.64703	estimated	*Lepus europaeus*	NA	0	0	1	NA	0	0	1	NA	NA	NA	NA	NA	NA	NA	NA	NA

134BYMX090120	GER-S		unknown	Donnersdorf	49.96972	10.41892	estimated	*Lepus europaeus*	NA	0	0	1	NA	0	1	0	NA	NA	NA	NA	NA	NA	NA	NA	NA
135BYFX230120	GER-S		unknown	Wackersdorf	49.31377	12.17892	estimated	*Lepus europaeus*	**ON089557**	0	0	1	NA	1	0	0	OM990885	GGAGGC	GG	AAAGGTGGAGGC	KGGG	I	3	AAA	K
136BYMX120220	GER-S		unknown	Madenhausen	50.13667	10.30056	estimated	*Lepus europaeus*	NA	0	0	1	NA	0	0	1	NA	NA	NA	NA	NA	NA	NA	NA	NA
137BYMX130220	GER-S		unknown	Oettingen	48.95246	10.60368	estimated	*Lepus europaeus*	NA	0	0	1	NA	0	0	1	NA	NA	NA	NA	NA	NA	NA	NA	NA
138BYMX250220	GER-S		unknown	Buckenhof Erlangen	49.59158	11.05101	estimated	*Lepus europaeus*	NA	0	0	1	NA	0	0	1	NA	NA	NA	NA	NA	NA	NA	NA	NA
01SHFX240419	GER-N		unknown	Schleswig Holstein	54.12985	8.85870	estimated	*Lepus europaeus*	**ON089365**	1	0	0	OM939694	1	0	0	**OM990859**	GGAGGC	GG	AAAGGTGGAGGC	KGGG	I	3	AAA	K
02SHMX270419	GER-N		unknown	Schleswig Holstein	54.12985	8.85870	estimated	*Lepus europaeus*	NA	0	0	1	NA	0	1	0	NA	NA	NA	NA	NA	NA	NA	NA	NA
03SHFX280519	GER-N		unknown	Schleswig Holstein	54.12985	8.85870	estimated	*Lepus europaeus*	NA	0	0	1	NA	0	0	1	NA	NA	NA	NA	NA	NA	NA	NA	NA
04SHFX020919	GER-N		unknown	Schleswig Holstein	54.12985	8.85870	estimated	*Lepus europaeus*	NA	0	0	1	NA	0	0	1	NA	NA	NA	NA	NA	NA	NA	NA	NA
05SHFX020919	GER-N		unknown	Schleswig Holstein	54.12985	8.85870	estimated	*Lepus europaeus*	NA	0	0	1	NA	0	0	1	NA	NA	NA	NA	NA	NA	NA	NA	NA
06SHMX030919	GER-N		unknown	Schleswig Holstein	54.12985	8.85870	estimated	*Lepus europaeus*	NA	0	0	1	NA	0	0	1	NA	NA	NA	NA	NA	NA	NA	NA	NA
07SHMX030919	GER-N		unknown	Schleswig Holstein	54.12985	8.85870	estimated	*Lepus europaeus*	NA	0	0	1	NA	0	0	1	NA	NA	NA	NA	NA	NA	NA	NA	NA
08SHFX040919	GER-N		unknown	Schleswig Holstein	54.12985	8.85870	estimated	*Lepus europaeus*	NA	0	0	1	NA	0	0	1	NA	NA	NA	NA	NA	NA	NA	NA	NA
09SHMX120919	GER-N		unknown	Schleswig Holstein	54.12985	8.85870	estimated	*Lepus europaeus*	NA	0	0	1	NA	0	0	1	NA	NA	NA	NA	NA	NA	NA	NA	NA
10SHMX120919	GER-N		unknown	Schleswig Holstein	54.12985	8.85870	estimated	*Lepus europaeus*	NA	0	0	1	NA	0	0	1	NA	NA	NA	NA	NA	NA	NA	NA	NA
11SHFX021019	GER-N		unknown	Schleswig Holstein	54.12985	8.85870	estimated	*Lepus europaeus*	NA	0	0	1	NA	0	0	1	NA	NA	NA	NA	NA	NA	NA	NA	NA
12SHMX151019	GER-N		unknown	Schleswig Holstein	54.12985	8.85870	estimated	*Lepus europaeus*	NA	0	0	1	NA	0	0	1	NA	NA	NA	NA	NA	NA	NA	NA	NA
13SHMX151019	GER-N		unknown	Schleswig Holstein	54.12985	8.85870	estimated	*Lepus europaeus*	**ON089406**	0	1	0	NA	0	1	0	NA	NA	NA	NA	NA	NA	NA	NA	NA
14SHMX090919	GER-N		unknown	Schleswig Holstein	54.12985	8.85870	estimated	*Lepus europaeus*	NA	0	0	1	NA	0	0	1	NA	NA	NA	NA	NA	NA	NA	NA	NA
15SHFX030719	GER-N		unknown	Schleswig Holstein	54.12985	8.85870	estimated	*Lepus europaeus*	**ON089414**	0	0	1	NA	1	0	0	**OM990905**	AGAGGC	RG	AAAGGTGGAGGC	KGGG	I	2	AAA	K
16SHMX030719	GER-N		unknown	Schleswig Holstein	54.12985	8.85870	estimated	*Lepus europaeus*	NA	0	0	1	NA	0	0	1	NA	NA	NA	NA	NA	NA	NA	NA	NA
17SHFX030719	GER-N		unknown	Schleswig Holstein	54.12985	8.85870	estimated	*Lepus europaeus*	NA	0	0	1	NA	0	1	0	NA	NA	NA	NA	NA	NA	NA	NA	NA
18SHFX030719	GER-N		unknown	Schleswig Holstein	54.12985	8.85870	estimated	*Lepus europaeus*	NA	0	0	1	NA	0	0	1	NA	NA	NA	NA	NA	NA	NA	NA	NA
46SHF1091119	GER-N		0	Tetenbüll	54.376432	8.834058	estimated	*Lepus europaeus*	NA	0	0	1	NA	0	0	1	NA	NA	NA	NA	NA	NA	NA	NA	NA
47SHM1091119	GER-N		0	Tetenbüll	54.376432	8.834058	estimated	*Lepus europaeus*	**ON089455**	0	1	0	NA	0	1	0	NA	NA	NA	NA	NA	NA	NA	NA	NA
48SHM1091119	GER-N		0	Tetenbüll	54.376432	8.834058	estimated	*Lepus europaeus*	**ON089456**	0	0	1	NA	1	0	0	**OM990966**	GGAGGC	GG	AAACAGGGAGGC	KQGG	III	3	AAA	K
49SHM1091119	GER-N		0	Tetenbüll	54.376432	8.834058	estimated	*Lepus europaeus*	NA	0	0	1	NA	0	1	0	NA	NA	NA	NA	NA	NA	NA	NA	NA
50SHM1091119	GER-N		0	Tetenbüll	54.376432	8.834058	estimated	*Lepus europaeus*	**ON089460**	0	0	1	NA	1	0	0	**OM990971**	GGAGGC	GG	AAAGGTGGAGGC	KGGG	I	1	AAAGGCAAA	KGK
51SHF1091119	GER-N		0	Tetenbüll	54.376432	8.834058	estimated	*Lepus europaeus*	NA	0	0	1	NA	0	0	1	NA	NA	NA	NA	NA	NA	NA	NA	NA
52SHM1091119	GER-N		0	Tetenbüll	54.376432	8.834058	estimated	*Lepus europaeus*	NA	0	0	1	NA	0	0	1	NA	NA	NA	NA	NA	NA	NA	NA	NA
53SHM1091119	GER-N		0	Tetenbüll	54.376432	8.834058	estimated	*Lepus europaeus*	NA	0	0	1	NA	0	0	1	NA	NA	NA	NA	NA	NA	NA	NA	NA
54SHF1091119	GER-N		0	Tetenbüll	54.376432	8.834058	estimated	*Lepus europaeus*	NA	0	0	1	NA	0	1	0	NA	NA	NA	NA	NA	NA	NA	NA	NA
55SHF1091119	GER-N		0	Tetenbüll	54.376432	8.834058	estimated	*Lepus europaeus*	**ON089464**	0	0	1	NA	0	1	0	NA	NA	NA	NA	NA	NA	NA	NA	NA
56SHF1101119	GER-N		0	Fehmarn	54.471025	11.139297	estimated	*Lepus europaeus*	**ON089465**	0	0	1	NA	1	0	0	**OM990974**	GGAGGC	GG	AAAGGAGGC	KGG	IV	3	AAA	K
57SHM1101119	GER-N		0	Fehmarn	54.471025	11.139297	estimated	*Lepus europaeus*	**ON089466**	0	0	1	NA	1	0	0	OM990974	GGAGGC	GG	AAAGGAGGC	KGG	IV	3	AAA	K
58SHF1101119	GER-N		0	Fehmarn	54.471025	11.139297	estimated	*Lepus europaeus*	**ON089467**	0	0	1	NA	1	0	0	**OM990977**	GTAGGC	VG	AAAGGTGGAGGC	KGGG	I	4	AAA	K
59SHF1101119	GER-N		0	Fehmarn	54.471025	11.139297	estimated	*Lepus europaeus*	NA	0	0	1	NA	0	0	1	NA	NA	NA	NA	NA	NA	NA	NA	NA
60SHF1101119	GER-N		0	Fehmarn	54.471025	11.139297	estimated	*Lepus europaeus*	**ON089470**	0	0	1	NA	1	0	0	OM990966	GGAGGC	GG	AAACAGGGAGGC	KQGG	III	3	AAA	K
61SHF1101119	GER-N		0	Fehmarn	54.471025	11.139297	estimated	*Lepus europaeus*	**ON089472**	0	0	1	NA	1	0	0	**OM990982**	GGAGGC	GG	AAAGGTGGAGGC	KGGG	I	6	AAA	K
62SHM1101119	GER-N		0	Fehmarn	54.471025	11.139297	estimated	*Lepus europaeus*	NA	0	0	1	NA	0	0	1	NA	NA	NA	NA	NA	NA	NA	NA	NA
63SHF1101119	GER-N		0	Fehmarn	54.471025	11.139297	estimated	*Lepus europaeus*	**ON089475**	0	0	1	NA	1	0	0	**OM990983**	GGAGGC	GG	AAAGGTGGAGGC	KGGG	I	2	AAA	K
64SHM1101119	GER-N		0	Fehmarn	54.471025	11.139297	estimated	*Lepus europaeus*	**ON089478**	0	0	1	NA	1	0	0	**OM990987**	GGAGGC	GG	AAAGGTGGAGGC	KGGG	NA	3	AAAGGAGGCAAA	KGGK
65SHM1151119	GER-N		0	Neu Duvenstedt	54.358635	9.702435	estimated	*Lepus europaeus*	**ON089480**	0	0	1	NA	1	0	0	**OM990990**	GGAGGC	GG	AAAGGTGGAGGC	KGGG	I	3	AAAGGCAAA	KGK
66SHM1151119	GER-N		0	Neu Duvenstedt	54.358635	9.702435	estimated	*Lepus europaeus*	**ON089482**	0	0	1	NA	1	0	0	**OM990991**	GGAGGC	GG	AAAGGAGGC	KGG	IV	3	AAA	K
67SHF1151119	GER-N		0	Neu Duvenstedt	54.358635	9.702435	estimated	*Lepus europaeus*	**ON089484**	0	0	1	NA	1	0	0	**OM990993**	GTAGGC	VG	AAAGGTGGAGGC	KGGG	NA	4	AAAGGAGGCAAA	KGGK
68SHF1151119	GER-N		0	Neu Duvenstedt	54.358635	9.702435	estimated	*Lepus europaeus*	**ON089486**	0	0	1	NA	1	0	0	OM990990	GGAGGC	GG	AAAGGTGGAGGC	KGGG	I	3	AAAGGCAAA	KGK
69SHF1151119	GER-N		0	Neu Duvenstedt	54.358635	9.702435	estimated	*Lepus europaeus*	**ON089488**	1	0	0	OM939694	1	0	0	OM990990	GGAGGC	GG	AAAGGTGGAGGC	KGGG	I	3	AAAGGCAAA	KGK
70SHF1161119	GER-N		0	Föhr	54.718197	8.502957	estimated	*Lepus europaeus*	**ON089490**	0	1	0	NA	0	1	0	NA	NA	NA	NA	NA	NA	NA	NA	NA
71SHF1161119	GER-N		0	Föhr	54.718197	8.502957	estimated	*Lepus europaeus*	**ON089491**	1	0	0	OM939694	1	0	0	**OM990997**	GGAGGC	GG	AAAGGTGGAGGC	KGGG	I	2	AAA	K
72SHF1161119	GER-N		0	Föhr	54.718197	8.502957	estimated	*Lepus europaeus*	**ON089492**	0	0	1	NA	1	0	0	OM990997	GGAGGC	GG	AAAGGTGGAGGC	KGGG	I	2	AAA	K
73SHM1161119	GER-N		0	Föhr	54.718197	8.502957	estimated	*Lepus europaeus*	NA	0	0	1	NA	0	1	0	NA	NA	NA	NA	NA	NA	NA	NA	NA
74SHM1161119	GER-N		0	Föhr	54.718197	8.502957	estimated	*Lepus europaeus*	**ON089495**	0	0	1	NA	1	0	0	OM990997	GGAGGC	GG	AAAGGTGGAGGC	KGGG	I	2	AAA	K
75SHM1161119	GER-N		0	Föhr	54.718197	8.502957	estimated	*Lepus europaeus*	**ON089496**	0	0	1	NA	1	0	0	**OM991001**	GGAGGC	GG	AAACAGGGAGGC	KQGG	III	1	AAA	K
76SHM1161119	GER-N		0	Föhr	54.718197	8.502957	estimated	*Lepus europaeus*	NA	0	1	0	NA	0	1	0	NA	NA	NA	NA	NA	NA	NA	NA	NA
77SHM1161119	GER-N		0	Föhr	54.718197	8.502957	estimated	*Lepus europaeus*	**ON089500**	0	0	1	NA	1	0	0	OM991001	GGAGGC	GG	AAACAGGGAGGC	KQGG	III	1	AAA	K
78SHM1161119	GER-N		0	Föhr	54.718197	8.502957	estimated	*Lepus europaeus*	**ON089503**	0	1	0	NA	1	0	0	**OM991004**	GGAGGCAAAGGTAGAGGC	GGKGRG	AAAGGTGGAGGC	KGGG	NA	1	AAA	K
79SHM1161119	GER-N		0	Föhr	54.718197	8.502957	estimated	*Lepus europaeus*	NA	0	0	1	NA	0	0	1	NA	NA	NA	NA	NA	NA	NA	NA	NA
80SHF1231119	GER-N		0	Elpersbüttel	54.066881	9.032187	estimated	*Lepus europaeus*	NA	0	0	1	NA	0	0	1	NA	NA	NA	NA	NA	NA	NA	NA	NA
81SHF1231119	GER-N		0	Elpersbüttel	54.066881	9.032187	estimated	*Lepus europaeus*	**ON089508**	0	0	1	NA	1	0	0	**OM991008**	GGAGGC	GG	AAAGGTGGAGGC	KGGG	I	1	AAA	K
82SHF1231119	GER-N		0	Elpersbüttel	54.066881	9.032187	estimated	*Lepus europaeus*	**ON089510**	0	0	1	NA	1	0	0	**OM991009**	GGAGGC	GG	AAAGGTGGAGGC	KGGG	I	2	AAA	K
83SHM1231119	GER-N		0	Elpersbüttel	54.066881	9.032187	estimated	*Lepus europaeus*	NA	0	0	1	NA	0	0	1	NA	NA	NA	NA	NA	NA	NA	NA	NA
84SHF1231119	GER-N		0	Elpersbüttel	54.066881	9.032187	estimated	*Lepus europaeus*	**ON089512**	0	0	1	NA	1	0	0	**OM991010**	GGAGGC	GG	AAAGGAGGC	KGG	IV	2	AAA	K
85SHF1231119	GER-N		0	Elpersbüttel	54.066881	9.032187	estimated	*Lepus europaeus*	NA	0	0	1	NA	1	0	0	OM990997	GGAGGC	GG	AAAGGTGGAGGC	KGGG	I	2	AAA	K
86SHF1231119	GER-N		0	Elpersbüttel	54.066881	9.032187	estimated	*Lepus europaeus*	NA	0	0	1	NA	0	0	1	NA	NA	NA	NA	NA	NA	NA	NA	NA
87SHF1231119	GER-N		0	Elpersbüttel	54.066881	9.032187	estimated	*Lepus europaeus*	NA	0	0	1	NA	0	0	1	NA	NA	NA	NA	NA	NA	NA	NA	NA
88SHF1231119	GER-N		0	Elpersbüttel	54.066881	9.032187	estimated	*Lepus europaeus*	NA	0	0	1	NA	0	0	1	NA	NA	NA	NA	NA	NA	NA	NA	NA
89SHM1231119	GER-N		0	Elpersbüttel	54.066881	9.032187	estimated	*Lepus europaeus*	NA	0	0	1	NA	0	0	1	NA	NA	NA	NA	NA	NA	NA	NA	NA
90SHM1241119	GER-N		0	Vaalermoor	53.980052	9.332179	estimated	*Lepus europaeus*	NA	0	0	1	NA	0	0	1	NA	NA	NA	NA	NA	NA	NA	NA	NA
91SHF1241119	GER-N		0	Vaalermoor	53.980052	9.332179	estimated	*Lepus europaeus*	NA	0	0	1	NA	0	0	1	NA	NA	NA	NA	NA	NA	NA	NA	NA
92SHM1241119	GER-N		0	Vaalermoor	53.980052	9.332179	estimated	*Lepus europaeus*	NA	0	0	1	NA	0	0	1	NA	NA	NA	NA	NA	NA	NA	NA	NA
93SHF1241119	GER-N		0	Vaalermoor	53.980052	9.332179	estimated	*Lepus europaeus*	**ON089519**	1	0	0	OM939694	1	0	0	**OM991016**	GGAGGC	GG	AAAGGTGGAGGC	KGGG	I	3	AAA	K
94SHF1071219	GER-N		0	Friedrichskoog	54.006452	8.911301	estimated	*Lepus europaeus*	NA	0	0	1	NA	0	0	1	NA	NA	NA	NA	NA	NA	NA	NA	NA
95SHM1071219	GER-N		0	Friedrichskoog	54.006453	8.911302	estimated	*Lepus europaeus*	**ON089520**	0	0	1	NA	1	0	0	**OM991019**	GGAGGC	GG	AAAGGTGGAGGC	KGGG	I	3	AAA	K
96SHM1071219	GER-N		0	Friedrichskoog	54.006454	8.911303	estimated	*Lepus europaeus*	NA	0	0	1	NA	0	0	1	NA	NA	NA	NA	NA	NA	NA	NA	NA
97SHM1241119	GER-N		0	Vaalermoor	53.980052	9.332179	estimated	*Lepus europaeus*	**ON089523**	1	0	0	OM939694	1	0	0	**OM991022**	GGAGGC	GG	AAAGGTGGAGGC	KGGG	I	1	AAA	K
98SHF1071219	GER-N		0	Friedrichskoog	54.006452	8.911301	estimated	*Lepus europaeus*	NA	0	0	1	NA	0	0	1	NA	NA	NA	NA	NA	NA	NA	NA	NA
99SHM1071219	GER-N		0	Friedrichskoog	54.006452	8.911301	estimated	*Lepus europaeus*	NA	0	0	1	NA	0	0	1	NA	NA	NA	NA	NA	NA	NA	NA	NA
100SHM1071219	GER-N		0	Friedrichskoog	54.006452	8.911301	estimated	*Lepus europaeus*	NA	0	0	1	NA	0	0	1	NA	NA	NA	NA	NA	NA	NA	NA	NA
101SHF1071219	GER-N		0	Friedrichskoog	54.006452	8.911301	estimated	*Lepus europaeus*	NA	0	0	1	NA	0	0	1	NA	NA	NA	NA	NA	NA	NA	NA	NA
102SHF1071219	GER-N		0	Friedrichskoog	54.006452	8.911301	estimated	*Lepus europaeus*	**ON089528**	0	1	0	NA	1	0	0	**OM991028**	GGAGGC	GG	AAAGGTGGAGGC	KGGG	I	3	AAA	K
103SHM1071219	GER-N		0	Friedrichskoog	54.006452	8.911301	estimated	*Lepus europaeus*	NA	0	0	1	NA	0	0	1	NA	NA	NA	NA	NA	NA	NA	NA	NA
104SHF1071219	GER-N		0	Friedrichskoog	54.006452	8.911301	estimated	*Lepus europaeus*	**ON089530**	0	1	0	NA	1	0	0	**OM991029**	GGAGGC	GG	AAAGGTGGAGGC	KGGG	NA	2	AAAGGAGGCAAA	KGGK
105SHF1091219	GER-N		0	Wesselburener Deichhausen	54.181110	8.941514	estimated	*Lepus europaeus*	NA	0	0	1	NA	0	1	0	NA	NA	NA	NA	NA	NA	NA	NA	NA
106SHM1111219	GER-N		0	Kreis Pinneberg	53.719178	9.757192	estimated	*Oryctolagus cuniculus*	NA	0	0	1	NA	0	1	0	NA	NA	NA	NA	NA	NA	NA	NA	NA
107SHM1091219	GER-N		0	Klein Nordende	53.722232	9.673377	estimated	*Oryctolagus cuniculus*	**ON089533**	0	0	1	NA	0	0	1	NA	NA	NA	NA	NA	NA	NA	NA	NA
108SHM1091219	GER-N		0	Kreis Pinneberg	53.719178	9.757192	estimated	*Oryctolagus cuniculus*	**ON089534**	0	0	1	NA	0	0	1	NA	NA	NA	NA	NA	NA	NA	NA	NA
109SHF1111219	GER-N		0	Fehmarn	54.471025	11.139297	estimated	*Lepus europaeus*	NA	0	0	1	NA	0	0	1	NA	NA	NA	NA	NA	NA	NA	NA	NA
110SHF1111219	GER-N		0	Fehmarn	54.471025	11.139297	estimated	*Lepus europaeus*	NA	0	0	1	NA	0	0	1	NA	NA	NA	NA	NA	NA	NA	NA	NA
111SHF1141219	GER-N		0	Haale	54.173280	9.545860	estimated	*Lepus europaeus*	**ON089537**	0	1	0	NA	0	1	0	NA	NA	NA	NA	NA	NA	NA	NA	NA
112SHF1141219	GER-N		0	Haale	54.173280	9.545860	estimated	*Lepus europaeus*	**ON089539**	0	0	1	NA	1	0	0	**OM991037**	GGAGGC	GG	AAAGGTGGAGGC	KGGG	I	2	AAAGGCAAA	KGK
113SHM1141219	GER-N		0	Haale	54.173280	9.545860	estimated	*Lepus europaeus*	NA	0	0	1	NA	0	0	1	NA	NA	NA	NA	NA	NA	NA	NA	NA
114SHM1141219	GER-N		0	Haale	54.173280	9.545860	estimated	*Lepus europaeus*	NA	0	0	1	NA	0	0	1	NA	NA	NA	NA	NA	NA	NA	NA	NA
115SHM1141219	GER-N		0	Haale	54.173280	9.545860	estimated	*Lepus europaeus*	NA	0	0	1	NA	0	0	1	NA	NA	NA	NA	NA	NA	NA	NA	NA
116SHF2141219	GER-N		1	Haale	54.173280	9.545860	estimated	*Lepus europaeus*	**ON089541**	0	0	1	NA	1	0	0	**OM991039**	GGAGGC	GG	AAAGGTGGAGGC	KGGG	NA	3	(AAAGGAGGC)2AAA	(KGG)2K
117SHM1141219	GER-N		0	Haale	54.173280	9.545860	estimated	*Lepus europaeus*	NA	0	0	1	NA	0	0	1	NA	NA	NA	NA	NA	NA	NA	NA	NA
118SHM1141219	GER-N		0	Haale	54.173280	9.545860	estimated	*Lepus europaeus*	NA	0	0	1	NA	0	0	1	NA	NA	NA	NA	NA	NA	NA	NA	NA
119SHF2141219	GER-N		1	Haale	54.173280	9.545860	estimated	*Lepus europaeus*	**ON089545**	1	0	0	OM939694	1	0	0	**OM991043**	GGAGGC	GG	AAAGGTGGAGGC	KGGG	I	2	AAA	K
120SHM1141219	GER-N		0	Haale	54.173280	9.545860	estimated	*Lepus europaeus*	NA	0	0	1	NA	0	0	1	NA	NA	NA	NA	NA	NA	NA	NA	NA
121SHF1131219	GER-N		0	Fehmarn	54.472036	11.017582	estimated	*Lepus europaeus*	NA	0	0	1	NA	0	0	1	NA	NA	NA	NA	NA	NA	NA	NA	NA
122SHM1131219	GER-N		0	Fehmarn	54.471025	11.139297	estimated	*Lepus europaeus*	NA	0	0	1	NA	0	0	1	NA	NA	NA	NA	NA	NA	NA	NA	NA
123SHM1131219	GER-N		0	Blomesche wildnis	53.803419	9.453468	estimated	*Lepus europaeus*	**ON089549**	0	0	1	NA	1	0	0	**OM991046**	GGAGGC	GG	AAAGGTGGAGGC	KGGG	I	2	AAA	K
124SHM1050220	GER-N		0	Wolmersdorf	54.074844	9.108853	estimated	*Lepus europaeus*	NA	0	0	1	NA	0	0	1	NA	NA	NA	NA	NA	NA	NA	NA	NA
125SHM1030120	GER-N		0	Witzwort	54.408839	9.002065	estimated	*Lepus europaeus*	NA	0	0	1	NA	0	0	1	NA	NA	NA	NA	NA	NA	NA	NA	NA
126SHM1091119	GER-N		0	Tetenbüll	54.376432	8.834058	estimated	*Lepus europaeus*	**ON089551**	0	1	0	NA	1	0	0	OM990987	GGAGGC	GG	AAAGGTGGAGGC	KGGG	NA	3	AAAGGAGGCAAA	KGGK

127SHF1101119	GER-N		0	Fehmarn	54.471025	11.139297	estimated	*Lepus europaeus*	NA	0	0	1	NA	0	0	1	NA	NA	NA	NA	NA	NA	NA	NA	NA
128SHFX170620	GER-N		unknown	Altwittenbek	54.12985	8.85870	estimated	*Lepus europaeus*	NA	0	0	1	NA	0	0	1	NA	NA	NA	NA	NA	NA	NA	NA	NA
129SHUX270121	GER-N		unknown	Schleswig Holstein	54.12985	8.85870	estimated	*Lepus europaeus*	NA	0	0	1	NA	0	0	1	NA	NA	NA	NA	NA	NA	NA	NA	NA
130SHMX011220	GER-N		unknown	Wolmersdorf	54.12985	8.85870	estimated	*Lepus europaeus*	NA	0	0	1	NA	0	0	1	NA	NA	NA	NA	NA	NA	NA	NA	NA
143SHFX301120	GER-N		unknown	Vaalermoor	54.12985	8.85870	estimated	*Lepus europaeus*	**ON089555**	0	1	0	NA	1	0	0	**OM991064**	GGAGGC	GG	AAAGGTGGAGGC	KGGG	I	3	AAA	K
144SHFX301120	GER-N		unknown	Vaalermoor	54.12985	8.85870	estimated	*Lepus europaeus*	**ON089558**	1	0	0	OM939694	0	1	0	NA	NA	NA	NA	NA	NA	NA	NA	NA
145SHMX301120	GER-N		unknown	Vaalermoor	54.12985	8.85870	estimated	*Lepus europaeus*	**ON089559**	1	0	0	OM939694	1	0	0	**OM991066**	GGAGGC	GG	AAAGGTGGAGGC	KGGG	I	3	AAA	K
147SHMX301120	GER-N		unknown	Vaalermoor	54.12985	8.85870	estimated	*Lepus europaeus*	**ON089560**	0	0	1	NA	0	1	0	NA	NA	NA	NA	NA	NA	NA	NA	NA
149SHMX301120	GER-N		unknown	Tetenbüll	54.12985	8.85870	estimated	*Lepus europaeus*	**ON089561**	0	0	1	NA	0	1	0	NA	NA	NA	NA	NA	NA	NA	NA	NA
151SHFX301120	GER-N		unknown	Tetenbüll	54.12985	8.85870	estimated	*Lepus europaeus*	**ON089562**	0	1	0	NA	1	0	0	**OM991068**	GTAGGC	VG	AAAGGTGGAGGC	KGGG	I	3	AAA	K
157SHFX301120	GER-N		unknown	Tetenbüll	54.12985	8.85870	estimated	*Lepus europaeus*	ON089569	0	1	0	NA	1	0	0	**OM991069**	GGAGGC	GG	AAAGGTGGAGGC	KGGG	NA	2	AAACGGGAAGGCAAA	KRGGK
158SHFX301120	GER-N		unknown	Vaalermoor	54.12985	8.85870	estimated	*Lepus europaeus*	NA	0	0	1	NA	0	0	1	NA	NA	NA	NA	NA	NA	NA	NA	NA
171SHFX121220	GER-N		unknown	Elpersbüttel	54.12985	8.85870	estimated	*Lepus europaeus*	**ON089563**	0	1	0	NA	0	1	0	NA	NA	NA	NA	NA	NA	NA	NA	NA
172SHMX121220	GER-N		unknown	Elpersbüttel	54.12985	8.85870	estimated	*Lepus europaeus*	**ON089564**	0	1	0	NA	0	1	0	NA	NA	NA	NA	NA	NA	NA	NA	NA
173SHMX121220	GER-N		unknown	Elpersbüttel	54.12985	8.85870	estimated	*Lepus europaeus*	NA	0	0	1	NA	0	0	1	NA	NA	NA	NA	NA	NA	NA	NA	NA
174SHMX121220	GER-N		unknown	Elpersbüttel	54.12985	8.85870	estimated	*Lepus europaeus*	**ON089565**	1	0	0	OM939694	1	0	0	**OM991070**	GGAGGC	GG	AAAGGTGGAGGC	KGGG	I	3	AAA	K
175SHFX121220	GER-N		unknown	Elpersbüttel	54.12985	8.85870	estimated	*Lepus europaeus*	**ON089566**	1	0	0	OM939694	0	1	0	NA	NA	NA	NA	NA	NA	NA	NA	NA
176SHMX121220	GER-N		unknown	Elpersbüttel	54.12985	8.85870	estimated	*Lepus europaeus*	NA	0	0	1	NA	0	0	1	NA	NA	NA	NA	NA	NA	NA	NA	NA
177SHFX121220	GER-N		unknown	Elpersbüttel	54.12985	8.85870	estimated	*Lepus europaeus*	NA	0	0	1	NA	0	0	1	NA	NA	NA	NA	NA	NA	NA	NA	NA
178SHMX121220	GER-N		unknown	Elpersbüttel	54.12985	8.85870	estimated	*Lepus europaeus*	NA	0	0	1	NA	0	0	1	NA	NA	NA	NA	NA	NA	NA	NA	NA
179SHMX121220	GER-N		unknown	Elpersbüttel	54.12985	8.85870	estimated	*Lepus europaeus*	**ON089567**	1	0	0	OM939693	1	0	0	**OM991071**	GGAGGC	GG	AAAGGTGGAGGC	KGGG	I	6	AAA	K
180SHMX121220	GER-N		unknown	Haale	54.12985	8.85870	estimated	*Lepus europaeus*	NA	0	0	1	NA	0	0	1	NA	NA	NA	NA	NA	NA	NA	NA	NA
181SHMX121220	GER-N		unknown	Haale	54.12985	8.85870	estimated	*Lepus europaeus*	**ON089568**	0	1	0	NA	0	1	0	NA	NA	NA	NA	NA	NA	NA	NA	NA
182SHFX121220	GER-N		unknown	Haale	54.12985	8.85870	estimated	*Lepus europaeus*	NA	0	0	1	NA	0	0	1	NA	NA	NA	NA	NA	NA	NA	NA	NA
183SHMX121220	GER-N		unknown	Haale	54.12985	8.85870	estimated	*Lepus europaeus*	ON089399	1	0	0	**OM939722**	0	1	0	NA	NA	NA	NA	NA	NA	NA	NA	NA
184SHMX121220	GER-N		unknown	Haale	54.12985	8.85870	estimated	*Lepus europaeus*	**ON089569**	0	1	0	NA	1	0	0	**OM991072**	GGAGGC	GG	AAAGGTGGAGGC	KGGG	I	4	AAA	K
185SHFX121220	GER-N		unknown	Haale	54.12985	8.85870	estimated	*Lepus europaeus*	NA	0	0	1	NA	0	0	1	NA	NA	NA	NA	NA	NA	NA	NA	NA
186SHMX121220	GER-N		unknown	Haale	54.12985	8.85870	estimated	*Lepus europaeus*	**ON089570**	1	0	0	**OM939721**	0	1	0	NA	NA	NA	NA	NA	NA	NA	NA	NA
187SHFX121220	GER-N		unknown	Haale	54.12985	8.85870	estimated	*Lepus europaeus*	**ON089571**	1	0	0	OM939694	1	0	0	**OM991073**	GGAGGC	GG	AAAGGTGGAGGC	KGGG	I	5	AAA	K
188SHMX121220	GER-N		unknown	Haale	54.12985	8.85870	estimated	*Lepus europaeus*	**ON089572**	0	1	0	NA	1	0	0	**OM991074**	GGAGGC	GG	AAAGGTGGAGGC	KGGG	I	3	AAA	K
189SHMX121220	GER-N		unknown	Haale	54.12985	8.85870	estimated	*Lepus europaeus*	NA	0	0	1	NA	0	0	1	NA	NA	NA	NA	NA	NA	NA	NA	NA
190SHFX080121	GER-N		unknown	Busenwurth	54.12985	8.85870	estimated	*Lepus europaeus*	ON089569	1	0	0	OM939694	0	1	0	NA	NA	NA	NA	NA	NA	NA	NA	NA
191SHFX270121	GER-N		unknown	Schleswig Holstein	54.12985	8.85870	estimated	*Lepus europaeus*	**ON089573**	0	0	1	NA	1	0	0	OM991070	GGAGGC	GG	AAAGGTGGAGGC	KGGG	I	3	AAA	K
01KSMX021119	GER-CW		unknown	Espenau	51.39134	9.45820	estimated	*Lepus europaeus*	**ON089364**	0	1	0	NA	0	1	0	NA	NA	NA	NA	NA	NA	NA	NA	NA
02KSFX021119	GER-CW		unknown	Espenau	51.39134	9.45820	estimated	*Lepus europaeus*	**ON089370**	1	0	0	**OM939710**	1	0	0	**OM990861**	GGAGGC	GG	AAAGGTGGAGGC	KGGG	I	3	AAA	K
01DNTWF1151019	GER-CW		0	Göttingen	51.56115	9.95845	accurate	*Oryctolagus cuniculus f. domestica*	NA	0	0	1	NA	0	0	1	NA	NA	NA	NA	NA	NA	NA	NA	NA
02DNTWF1100120	GER-CW		0	Göttingen	51.56115	9.95845	accurate	*Lepus europaeus*	NA	1	0	0	OM939694	1	0	0	OM990858	GGAGGC	GG	AAAGGTGGAGGC	KGGG	I	1	AAA	K
03DNTWF1100320	GER-CW		0	Göttingen	51.56115	9.95845	accurate	*Oryctolagus cuniculus f. domestica*	NA	0	0	1	NA	0	0	1	NA	NA	NA	NA	NA	NA	NA	NA	NA
01GIF2290120	GER-CW		1	*University Giessen*	50.572683	8.658077	estimated	*Oryctolagus cuniculus f. domestica*	NA	1	0	0	OM939694	1	0	0	**OM990858**	GGAGGC	GG	AAAGGTGGAGGC	KGGG	I	1	AAA	K
02GIFX261119	GER-CW		unknown	Gießen/ WinGERtshecke	50.56598	8.65653	estimated	*Oryctolagus cuniculus*	**ON089368**	0	0	1	NA	0	0	1	NA	NA	NA	NA	NA	NA	NA	NA	NA
01SOM1161119	GER-CW		0	Geseke	51.63935	8.46921	accurate	*Lepus europaeus*	NA	0	0	1	NA	0	0	1	NA	NA	NA	NA	NA	NA	NA	NA	NA
02SOM1161119	GER-CW		0	Geseke	51.63935	8.46921	accurate	*Lepus europaeus*	NA	0	0	1	NA	0	0	1	NA	NA	NA	NA	NA	NA	NA	NA	NA
03SOF1161119	GER-CW		0	Geseke	51.63935	8.46921	accurate	*Lepus europaeus*	NA	0	1	0	NA	0	0	1	NA	NA	NA	NA	NA	NA	NA	NA	NA
04SOF1161119	GER-CW		0	Geseke	51.63935	8.46921	accurate	*Lepus europaeus*	NA	0	0	1	NA	0	0	1	NA	NA	NA	NA	NA	NA	NA	NA	NA
05SOM1161119	GER-CW		0	Geseke	51.63935	8.46921	accurate	*Lepus europaeus*	NA	0	0	1	NA	0	0	1	NA	NA	NA	NA	NA	NA	NA	NA	NA
06SOM1161119	GER-CW		0	Geseke	51.63935	8.46921	accurate	*Lepus europaeus*	NA	0	0	1	NA	0	0	1	NA	NA	NA	NA	NA	NA	NA	NA	NA
07SOF1161119	GER-CW		0	Geseke	51.63935	8.46921	accurate	*Lepus europaeus*	NA	1	0	0	OM939694	1	0	0	**OM990879**	GGAGGCAAAGGTAGAGGC	GGKGRG	AAAGGTGGAGGC	KGGG	NA	1	AAA	K
08SOM1161119	GER-CW		0	Geseke	51.63935	8.46921	accurate	*Lepus europaeus*	NA	0	0	1	NA	0	0	1	NA	NA	NA	NA	NA	NA	NA	NA	NA
09SOM1161119	GER-CW		0	Geseke	51.63935	8.46921	accurate	*Lepus europaeus*	NA	0	0	1	NA	0	0	1	NA	NA	NA	NA	NA	NA	NA	NA	NA
10SOF1161119	GER-CW		0	Geseke	51.63935	8.46921	accurate	*Lepus europaeus*	NA	0	0	1	NA	0	0	1	NA	NA	NA	NA	NA	NA	NA	NA	NA
11SOM1161119	GER-CW		0	Geseke	51.63935	8.46921	accurate	*Lepus europaeus*	NA	0	0	1	NA	0	0	1	NA	NA	NA	NA	NA	NA	NA	NA	NA
12SOF1161119	GER-CW		0	Geseke	51.63935	8.46921	accurate	*Lepus europaeus*	NA	0	0	1	NA	0	0	1	NA	NA	NA	NA	NA	NA	NA	NA	NA
13SOM1161119	GER-CW		0	Geseke	51.63935	8.46921	accurate	*Lepus europaeus*	NA	1	0	0	**OM939711**	1	0	0	**OM990897**	GGAGGC	GG	AAAGGTGGAGGC	KGGG	I	3	AAA	K
14SOF1161119	GER-CW		0	Geseke	51.63935	8.46921	accurate	*Lepus europaeus*	NA	0	0	1	NA	0	0	1	NA	NA	NA	NA	NA	NA	NA	NA	NA
15SOF1161119	GER-CW		0	Geseke	51.63935	8.46921	accurate	*Lepus europaeus*	NA	0	0	1	NA	0	0	1	NA	NA	NA	NA	NA	NA	NA	NA	NA
16SOM1161119	GER-CW		0	Geseke	51.63935	8.46921	accurate	*Lepus europaeus*	NA	0	0	1	NA	0	0	1	NA	NA	NA	NA	NA	NA	NA	NA	NA
17SOM1161119	GER-CW		0	Geseke	51.63935	8.46921	accurate	*Lepus europaeus*	NA	0	0	1	NA	0	0	1	NA	NA	NA	NA	NA	NA	NA	NA	NA
18SOF1161119	GER-CW		0	Geseke	51.63935	8.46921	accurate	*Lepus europaeus*	NA	0	0	1	NA	0	0	1	NA	NA	NA	NA	NA	NA	NA	NA	NA
19SOM1161119	GER-CW		0	Geseke	51.63935	8.46921	accurate	*Lepus europaeus*	NA	0	0	1	NA	0	0	1	NA	NA	NA	NA	NA	NA	NA	NA	NA
20SOM1161119	GER-CW		0	Geseke	51.63935	8.46921	accurate	*Lepus europaeus*	NA	0	1	0	NA	0	1	0	NA	NA	NA	NA	NA	NA	NA	NA	NA
21SOF1161119	GER-CW		0	Geseke	51.63935	8.46921	accurate	*Lepus europaeus*	NA	0	0	1	NA	0	0	1	NA	NA	NA	NA	NA	NA	NA	NA	NA
22SOF1161119	GER-CW		0	Geseke	51.63935	8.46921	accurate	*Lepus europaeus*	NA	0	0	1	NA	0	0	1	NA	NA	NA	NA	NA	NA	NA	NA	NA
23SOF1161119	GER-CW		0	Geseke	51.63935	8.46921	accurate	*Lepus europaeus*	NA	0	1	0	NA	0	1	0	NA	NA	NA	NA	NA	NA	NA	NA	NA
24SOM1161119	GER-CW		0	Geseke	51.63935	8.46921	accurate	*Lepus europaeus*	NA	0	1	0	NA	0	1	0	NA	NA	NA	NA	NA	NA	NA	NA	NA
01LRM1231119	GER-S		0	Mahlberg	48.29240	7.79968	accurate	*Lepus europaeus*	NA	0	1	0	NA	0	0	1	NA	NA	NA	NA	NA	NA	NA	NA	NA
02LRF1231119	GER-S		0	Mahlberg	48.29240	7.79968	accurate	*Lepus europaeus*	NA	0	0	1	NA	0	0	1	NA	NA	NA	NA	NA	NA	NA	NA	NA
03LRM1231119	GER-S		0	Mahlberg	48.29240	7.79968	accurate	*Lepus europaeus*	NA	0	1	0	NA	0	0	1	NA	NA	NA	NA	NA	NA	NA	NA	NA
04LRF1231119	GER-S		0	Mahlberg	48.29240	7.79968	accurate	*Lepus europaeus*	NA	0	0	1	NA	0	0	1	NA	NA	NA	NA	NA	NA	NA	NA	NA
05LRF1231119	GER-S		0	Mahlberg	48.29240	7.79968	accurate	*Lepus europaeus*	NA	0	0	1	NA	0	0	1	NA	NA	NA	NA	NA	NA	NA	NA	NA
06LRF1231119	GER-S		0	Mahlberg	48.29240	7.79968	accurate	*Lepus europaeus*	NA	0	0	1	NA	0	0	1	NA	NA	NA	NA	NA	NA	NA	NA	NA
07LRF1231119	GER-S		0	Mahlberg	48.29240	7.79968	accurate	*Lepus europaeus*	NA	1	0	0	OM939693	1	0	0	**OM990878**	GGAGGC	GG	AAAGGTGGAGGC	KGGG	I	1	AAA	K
08LRM1231119	GER-S		0	Mahlberg	48.29240	7.79968	accurate	*Lepus europaeus*	NA	0	0	1	NA	0	0	1	NA	NA	NA	NA	NA	NA	NA	NA	NA
09LRF1231119	GER-S		0	Mahlberg	48.29240	7.79968	accurate	*Lepus europaeus*	NA	1	0	0	OM939693	1	0	0	**OM990882**	GGAGGC	GG	AAAGGTGGAGGC	KGGG	I	2	AAA	K
10LRF1231119	GER-S		0	Mahlberg	48.29240	7.79968	accurate	*Lepus europaeus*	NA	0	1	0	NA	0	1	0	NA	NA	NA	NA	NA	NA	NA	NA	NA
11LRM1231119	GER-S		0	Mahlberg	48.29240	7.79968	accurate	*Lepus europaeus*	NA	0	0	1	NA	0	0	1	NA	NA	NA	NA	NA	NA	NA	NA	NA
12LRM1231119	GER-S		0	Mahlberg	48.29240	7.79968	accurate	*Lepus europaeus*	NA	0	0	1	NA	0	0	1	NA	NA	NA	NA	NA	NA	NA	NA	NA
13LRM1231119	GER-S		0	Mahlberg	48.29240	7.79968	accurate	*Lepus europaeus*	NA	0	0	1	NA	0	0	1	NA	NA	NA	NA	NA	NA	NA	NA	NA
14LRM1231119	GER-S		0	Mahlberg	48.29240	7.79968	accurate	*Lepus europaeus*	NA	0	0	1	NA	0	0	1	NA	NA	NA	NA	NA	NA	NA	NA	NA
01FZM1301119	GER-CW		0	Fritzlar	51.10312	9.32375	accurate	*Lepus europaeus*	NA	1	0	0	**OM939694**	1	0	0	**OM990857**	GGAGGC	GG	AAAGGTGGAGGC	KGGG	I	3	AAA	K
02FZF1301119	GER-CW		0	Fritzlar	51.10312	9.32375	accurate	*Lepus europaeus*	NA	0	0	1	NA	0	0	1	NA	NA	NA	NA	NA	NA	NA	NA	NA
03FZF1301119	GER-CW		0	Fritzlar	51.10312	9.32375	accurate	*Lepus europaeus*	NA	0	0	1	NA	0	0	1	NA	NA	NA	NA	NA	NA	NA	NA	NA
04FZM1301119	GER-CW		0	Fritzlar	51.10312	9.32375	accurate	*Lepus europaeus*	NA	1	0	0	**OM939706**	0	1	0	NA	NA	NA	NA	NA	NA	NA	NA	NA
05FZM1301119	GER-CW		0	Fritzlar	51.10312	9.32375	accurate	*Lepus europaeus*	NA	1	0	0	OM939693	1	0	0	**OM990867**	NA	NA	AAAGGCGGAGGC	KGGG	I	4	AAAGGCAAA	KGK
06FZM1301119	GER-CW		0	Fritzlar	51.10312	9.32375	accurate	*Lepus europaeus*	NA	0	0	1	NA	0	0	1	NA	NA	NA	NA	NA	NA	NA	NA	NA
07FZF1301119	GER-CW		0	Fritzlar	51.10312	9.32375	accurate	*Lepus europaeus*	NA	0	1	0	NA	0	1	0	NA	NA	NA	NA	NA	NA	NA	NA	NA
08FZF1301119	GER-CW		0	Fritzlar	51.10312	9.32375	accurate	*Lepus europaeus*	NA	0	0	1	NA	0	0	1	NA	NA	NA	NA	NA	NA	NA	NA	NA
09FZM1301119	GER-CW		0	Fritzlar	51.10312	9.32375	accurate	*Lepus europaeus*	NA	0	1	0	NA	0	1	0	NA	NA	NA	NA	NA	NA	NA	NA	NA
10FZM1301119	GER-CW		0	Fritzlar	51.10312	9.32375	accurate	*Lepus europaeus*	NA	0	0	1	NA	0	0	1	NA	NA	NA	NA	NA	NA	NA	NA	NA
11FZM1301119	GER-CW		0	Fritzlar	51.10312	9.32375	accurate	*Lepus europaeus*	NA	1	0	0	OM939693	1	0	0	**OM990888**	GGAGGCAAAGGTAGAGGC	GGKGRG	AAAGGTGGAGGC	KGGG	NA	1	AAA	K
12FZM1301119	GER-CW		0	Fritzlar	51.10312	9.32375	accurate	*Lepus europaeus*	NA	0	0	1	NA	0	0	1	NA	NA	NA	NA	NA	NA	NA	NA	NA
13FZM1301119	GER-CW		0	Fritzlar	51.10312	9.32375	accurate	*Lepus europaeus*	NA	0	0	1	NA	0	0	1	NA	NA	NA	NA	NA	NA	NA	NA	NA
14FZF1301119	GER-CW		0	Fritzlar	51.10312	9.32375	accurate	*Lepus europaeus*	NA	1	0	0	OM939694	1	0	0	**OM990898**	GGAGGC	GG	AAAGGTGGAGGC	KGGG	I	4	AAA	K
15FZM1301119	GER-CW		0	Fritzlar	51.10312	9.32375	accurate	*Lepus europaeus*	NA	1	0	0	OM939694	1	0	0	**OM990901**	NA	NA	AAAGGCCAAGGT	KGQG	NA	2	AAAGGCAAA	KGK
16FZM1301119	GER-CW		0	Fritzlar	51.10312	9.32375	accurate	*Lepus europaeus*	NA	1	0	0	OM939693	1	0	0	**OM990909**	NA	NA	AAAGGCGGAGGT	KGGG	I	3	AAAGGCAAA	KGK
17FZF1301119	GER-CW		0	Fritzlar	51.10312	9.32375	accurate	*Lepus europaeus*	NA	1	0	0	**OM939724**	1	0	0	**OM990912**	NA	NA	AAAGGCGGAGGT	KGGG	I	1	AAAGGCAAA	KGK
18FZM1301119	GER-CW		0	Fritzlar	51.10312	9.32375	accurate	*Lepus europaeus*	NA	0	0	1	NA	0	0	1	NA	NA	NA	NA	NA	NA	NA	NA	NA
19FZM1301119	GER-CW		0	Fritzlar	51.10312	9.32375	accurate	*Lepus europaeus*	NA	0	0	1	NA	0	0	1	NA	NA	NA	NA	NA	NA	NA	NA	NA
01FBF1041219	GER-CW		0	Nieder-Wöllstadt	50.26985	8.77197	accurate	*Lepus europaeus*	NA	0	0	1	NA	0	0	1	NA	NA	NA	NA	NA	NA	NA	NA	NA
02FBM1041219	GER-CW		0	Nieder-Wöllstadt	50.26985	8.77197	accurate	*Lepus europaeus*	NA	1	0	0	**OM939693**	1	0	0	**OM990860**	GGAGGC	GG	AAAGGTGGAGGC	KGGG	I	4	AAA	K
03FBF1041219	GER-CW		0	Nieder-Wöllstadt	50.26985	8.77197	accurate	*Lepus europaeus*	NA	0	0	1	NA	0	0	1	NA	NA	NA	NA	NA	NA	NA	NA	NA
04FBM1041219	GER-CW		0	Nieder-Wöllstadt	50.26985	8.77197	accurate	*Lepus europaeus*	NA	0	1	0	NA	1	0	0	OM990860	GGAGGC	GG	AAAGGTGGAGGC	KGGG	I	4	AAA	K
05FBM1041219	GER-CW		0	Nieder-Wöllstadt	50.26985	8.77197	accurate	*Lepus europaeus*	NA	0	1	0	NA	0	1	0	NA	NA	NA	NA	NA	NA	NA	NA	NA
06FBM1041219	GER-CW		0	Nieder-Wöllstadt	50.26985	8.77197	accurate	*Lepus europaeus*	NA	0	0	1	NA	0	0	1	NA	NA	NA	NA	NA	NA	NA	NA	NA

07FBM1041219	GER-CW		0	Nieder-Wöllstadt	50.26985	8.77197	accurate	*Lepus europaeus*	NA	0	1	0	NA	0	0	1	NA	NA	NA	NA	NA	NA	NA	NA	NA
08FBM1041219	GER-CW		0	Nieder-Wöllstadt	50.26985	8.77197	accurate	*Lepus europaeus*	NA	0	0	1	NA	0	0	1	NA	NA	NA	NA	NA	NA	NA	NA	NA
09FBF1041219	GER-CW		0	Nieder-Wöllstadt	50.26985	8.77197	accurate	*Lepus europaeus*	NA	0	0	1	NA	0	0	1	NA	NA	NA	NA	NA	NA	NA	NA	NA
10FBF1041219	GER-CW		0	Nieder-Wöllstadt	50.26985	8.77197	accurate	*Lepus europaeus*	NA	0	0	1	NA	0	0	1	NA	NA	NA	NA	NA	NA	NA	NA	NA
11FBM1041219	GER-CW		0	Nieder-Wöllstadt	50.26985	8.77197	accurate	*Lepus europaeus*	NA	0	1	0	NA	0	1	0	NA	NA	NA	NA	NA	NA	NA	NA	NA
12FBF1041219	GER-CW		0	Nieder-Wöllstadt	50.26985	8.77197	accurate	*Lepus europaeus*	NA	0	0	1	NA	0	0	1	NA	NA	NA	NA	NA	NA	NA	NA	NA
13FBF1041219	GER-CW		0	Nieder-Wöllstadt	50.26985	8.77197	accurate	*Lepus europaeus*	NA	0	0	1	NA	0	0	1	NA	NA	NA	NA	NA	NA	NA	NA	NA
14FBM1041219	GER-CW		0	Nieder-Wöllstadt	50.26985	8.77197	accurate	*Lepus europaeus*	NA	0	0	1	NA	0	0	1	NA	NA	NA	NA	NA	NA	NA	NA	NA
15FBF1041219	GER-CW		0	Nieder-Wöllstadt	50.26985	8.77197	accurate	*Lepus europaeus*	NA	0	0	1	NA	0	0	1	NA	NA	NA	NA	NA	NA	NA	NA	NA
16FBF1041219	GER-CW		0	Nieder-Wöllstadt	50.26985	8.77197	accurate	*Lepus europaeus*	NA	0	0	1	NA	0	0	1	NA	NA	NA	NA	NA	NA	NA	NA	NA
17FBM1041219	GER-CW		0	Nieder-Wöllstadt	50.26985	8.77197	accurate	*Lepus europaeus*	NA	0	0	1	NA	0	0	1	NA	NA	NA	NA	NA	NA	NA	NA	NA
18FBM1041219	GER-CW		0	Nieder-Wöllstadt	50.26985	8.77197	accurate	*Lepus europaeus*	NA	0	1	0	NA	0	1	0	NA	NA	NA	NA	NA	NA	NA	NA	NA
19FBF1041219	GER-CW		0	Nieder-Wöllstadt	50.26985	8.77197	accurate	*Lepus europaeus*	NA	0	0	1	NA	0	0	1	NA	NA	NA	NA	NA	NA	NA	NA	NA
20FBF1041219	GER-CW		0	Nieder-Wöllstadt	50.26985	8.77197	accurate	*Lepus europaeus*	NA	0	0	1	NA	0	0	1	NA	NA	NA	NA	NA	NA	NA	NA	NA
21FBM1041219	GER-CW		0	Nieder-Wöllstadt	50.26985	8.77197	accurate	*Lepus europaeus*	NA	0	1	0	NA	0	0	1	NA	NA	NA	NA	NA	NA	NA	NA	NA
22FBM1041219	GER-CW		0	Nieder-Wöllstadt	50.26985	8.77197	accurate	*Lepus europaeus*	NA	0	1	0	NA	0	1	0	NA	NA	NA	NA	NA	NA	NA	NA	NA
23FBM1041219	GER-CW		0	Nieder-Wöllstadt	50.26985	8.77197	accurate	*Lepus europaeus*	NA	0	0	1	NA	1	0	0	**OM990929**	NA	NA	AAAGGCGGAGGT	KGGG	I	5	AAAGGCAAA	KGK
24FBM1041219	GER-CW		0	Nieder-Wöllstadt	50.26985	8.77197	accurate	*Lepus europaeus*	NA	1	0	0	OM939693	1	0	0	**OM990931**	GGAGGC	GG	AAAGGTGGAGGC	KGGG	I	3	AAA	K
25FBF1041219	GER-CW		0	Nieder-Wöllstadt	50.26985	8.77197	accurate	*Lepus europaeus*	NA	0	0	1	NA	0	1	0	NA	NA	NA	NA	NA	NA	NA	NA	NA
26FBF1041219	GER-CW		0	Nieder-Wöllstadt	50.26985	8.77197	accurate	*Lepus europaeus*	NA	0	1	0	NA	0	0	1	NA	NA	NA	NA	NA	NA	NA	NA	NA
27FBF1041219	GER-CW		0	Nieder-Wöllstadt	50.26985	8.77197	accurate	*Lepus europaeus*	NA	0	0	1	NA	0	0	1	NA	NA	NA	NA	NA	NA	NA	NA	NA
28FBF1041219	GER-CW		0	Nieder-Wöllstadt	50.26985	8.77197	accurate	*Lepus europaeus*	NA	0	0	1	NA	0	0	1	NA	NA	NA	NA	NA	NA	NA	NA	NA
29FBM1041219	GER-CW		0	Nieder-Wöllstadt	50.26985	8.77197	accurate	*Lepus europaeus*	NA	0	0	1	NA	0	0	1	NA	NA	NA	NA	NA	NA	NA	NA	NA
30FBM1041219	GER-CW		0	Nieder-Wöllstadt	50.26985	8.77197	accurate	*Lepus europaeus*	NA	0	1	0	NA	0	1	0	NA	NA	NA	NA	NA	NA	NA	NA	NA
31FBM1041219	GER-CW		0	Nieder-Wöllstadt	50.26985	8.77197	accurate	*Lepus europaeus*	NA	1	0	0	**OM939705**	1	0	0	**OM990941**	GGAGGC	GG	AAAGGTGGAGGC	KGGG	I	2	AAAGGCAAA	KGK
32FBM1041219	GER-CW		0	Nieder-Wöllstadt	50.26985	8.77197	accurate	*Lepus europaeus*	NA	1	0	0	OM939705	1	0	0	**OM990942**	GGAGGC	GG	AAAGGTGGAGGC	KGGG	NA	4	AAACGGGGAGGCAAA	KRGGK
33FBF1041219	GER-CW		0	Nieder-Wöllstadt	50.26985	8.77197	accurate	*Lepus europaeus*	NA	0	0	1	NA	1	0	0	**OM990945**	GGAGGC	GG	AAAGGTGGAGGC	KGGG	I	6	AAA	K
34FBM1041219	GER-CW		0	Nieder-Wöllstadt	50.26985	8.77197	accurate	*Lepus europaeus*	NA	0	1	0	NA	0	1	0	NA	NA	NA	NA	NA	NA	NA	NA	NA
35FBM1041219	GER-CW		0	Nieder-Wöllstadt	50.26985	8.77197	accurate	*Lepus europaeus*	NA	0	1	0	NA	0	1	0	NA	NA	NA	NA	NA	NA	NA	NA	NA
36FBM1041219	GER-CW		0	Nieder-Wöllstadt	50.26985	8.77197	accurate	*Lepus europaeus*	NA	1	0	0	OM939694	1	0	0	**OM990949**	GGAGGC	GG	AAAGGTGGAGGC	KGGG	I	1	AAAGGCAAA	KGK
37FBF1041219	GER-CW		0	Nieder-Wöllstadt	50.26985	8.77197	accurate	*Lepus europaeus*	NA	0	0	1	NA	0	0	1	NA	NA	NA	NA	NA	NA	NA	NA	NA
38FBM1041219	GER-CW		0	Nieder-Wöllstadt	50.26985	8.77197	accurate	*Lepus europaeus*	NA	1	0	0	OM939693	1	0	0	OM990921	GGAGGC	GG	AAAGGTGGAGGC	KGGG	I	1	AAA	K
39FBF1041219	GER-CW		0	Nieder-Wöllstadt	50.26985	8.77197	accurate	*Lepus europaeus*	NA	1	0	0	OM939693	1	0	0	OM990860	GGAGGC	GG	AAAGGTGGAGGC	KGGG	I	4	AAA	K
40FBF1041219	GER-CW		0	Nieder-Wöllstadt	50.26985	8.77197	accurate	*Lepus europaeus*	NA	0	0	1	NA	0	0	1	NA	NA	NA	NA	NA	NA	NA	NA	NA
41FBM1041219	GER-CW		0	Nieder-Wöllstadt	50.26985	8.77197	accurate	*Lepus europaeus*	NA	0	0	1	NA	0	1	0	NA	NA	NA	NA	NA	NA	NA	NA	NA
42FBM1041219	GER-CW		0	Nieder-Wöllstadt	50.26985	8.77197	accurate	*Lepus europaeus*	NA	0	1	0	NA	0	0	1	NA	NA	NA	NA	NA	NA	NA	NA	NA
43FBF1041219	GER-CW		0	Nieder-Wöllstadt	50.26985	8.77197	accurate	*Lepus europaeus*	NA	1	0	0	OM939694	1	0	0	OM990942	GGAGGC	GG	AAAGGTGGAGGC	KGGG	NA	4	AAACGGGGAGGCAAA	KRGGK
44FBM1041219	GER-CW		0	Nieder-Wöllstadt	50.26985	8.77197	accurate	*Lepus europaeus*	NA	0	0	1	NA	0	0	1	NA	NA	NA	NA	NA	NA	NA	NA	NA
45FBF1041219	GER-CW		0	Nieder-Wöllstadt	50.26985	8.77197	accurate	*Lepus europaeus*	NA	0	0	1	NA	0	0	1	NA	NA	NA	NA	NA	NA	NA	NA	NA
46FBM1041219	GER-CW		0	Nieder-Wöllstadt	50.26985	8.77197	accurate	*Lepus europaeus*	NA	0	0	1	NA	0	0	1	NA	NA	NA	NA	NA	NA	NA	NA	NA
47FBM1041219	GER-CW		1	Nieder-Wöllstadt	50.26985	8.77197	accurate	*Lepus europaeus*	NA	0	1	0	NA	1	0	0	**OM990964**	GGAGGC	GG	AAAGGTGGAGGC	KGGG	I	4	AAA	K
48FBF1041219	GER-CW		0	Nieder-Wöllstadt	50.26985	8.77197	accurate	*Lepus europaeus*	NA	0	0	1	NA	0	0	1	NA	NA	NA	NA	NA	NA	NA	NA	NA
49FBM1041219	GER-CW		0	Nieder-Wöllstadt	50.26985	8.77197	accurate	*Lepus europaeus*	NA	0	1	0	NA	0	1	0	NA	NA	NA	NA	NA	NA	NA	NA	NA
50FBF1041219	GER-CW		0	Nieder-Wöllstadt	50.26985	8.77197	accurate	*Lepus europaeus*	NA	0	0	1	NA	0	0	1	NA	NA	NA	NA	NA	NA	NA	NA	NA
51FBF1041219	GER-CW		0	Nieder-Wöllstadt	50.26985	8.77197	accurate	*Lepus europaeus*	NA	0	1	0	NA	0	1	0	NA	NA	NA	NA	NA	NA	NA	NA	NA
52FBM1041219	GER-CW		0	Nieder-Wöllstadt	50.26985	8.77197	accurate	*Lepus europaeus*	NA	0	0	1	NA	0	1	0	NA	NA	NA	NA	NA	NA	NA	NA	NA
53FBM1041219	GER-CW		0	Nieder-Wöllstadt	50.26985	8.77197	accurate	*Lepus europaeus*	NA	0	1	0	NA	0	0	1	NA	NA	NA	NA	NA	NA	NA	NA	NA
54FBM1041219	GER-CW		0	Nieder-Wöllstadt	50.26985	8.77197	accurate	*Lepus europaeus*	NA	0	0	1	NA	0	0	1	NA	NA	NA	NA	NA	NA	NA	NA	NA
55FBM1041219	GER-CW		0	Nieder-Wöllstadt	50.26985	8.77197	accurate	*Lepus europaeus*	NA	0	0	1	NA	0	0	1	NA	NA	NA	NA	NA	NA	NA	NA	NA
01AUF1061219	GER-N		0	Riepe	53.37852	7.31262	accurate	*Lepus europaeus*	NA	1	0	0	**OM939698**	1	0	0	**OM990854**	GGAGGC	GG	AAAGGTGGAGGC	KGGG	I	4	AAA	K
02AUF1061219	GER-N		0	Riepe	53.37852	7.31262	accurate	*Lepus europaeus*	NA	1	0	0	OM939698	1	0	0	OM990854	GGAGGC	GG	AAAGGTGGAGGC	KGGG	I	4	AAA	K
03AUM1061219	GER-N		0	Riepe	53.37852	7.31262	accurate	*Lepus europaeus*	NA	0	1	0	NA	0	0	1	NA	NA	NA	NA	NA	NA	NA	NA	NA
04AUF1061219	GER-N		0	Riepe	53.37852	7.31262	accurate	*Lepus europaeus*	NA	0	0	1	NA	0	0	1	NA	NA	NA	NA	NA	NA	NA	NA	NA
05AUF1061219	GER-N		0	Riepe	53.37852	7.31262	accurate	*Lepus europaeus*	NA	0	0	1	NA	0	0	1	NA	NA	NA	NA	NA	NA	NA	NA	NA
06AUF1061219	GER-N		0	Riepe	53.37852	7.31262	accurate	*Lepus europaeus*	NA	1	0	0	**OM939719**	1	0	0	**OM990870**	GGAGGC	GG	AAAGGTGGAGGC	KGGG	NA	1	AAAGGTAGAGGCAAA	KGRGK
07AUF1061219	GER-N		0	Riepe	53.37852	7.31262	accurate	*Lepus europaeus*	NA	0	1	0	NA	0	1	0	NA	NA	NA	NA	NA	NA	NA	NA	NA
08AUF1061219	GER-N		0	Riepe	53.37852	7.31262	accurate	*Lepus europaeus*	NA	0	0	1	NA	0	0	1	NA	NA	NA	NA	NA	NA	NA	NA	NA
09AUF1061219	GER-N		0	Riepe	53.37852	7.31262	accurate	*Lepus europaeus*	NA	0	0	1	NA	0	0	1	NA	NA	NA	NA	NA	NA	NA	NA	NA
10AUF1061219	GER-N		0	Riepe	53.37852	7.31262	accurate	*Lepus europaeus*	NA	0	0	1	NA	1	0	0	**OM990883**	GGAGGC	GG	AAAGGTGGAGGC	KGGG	NA	1	(AAAAGTGGAGGC)2AAA	(KSGG)2K
11AUF1061219	GER-N		0	Riepe	53.37852	7.31262	accurate	*Lepus europaeus*	NA	1	0	0	OM939698	1	0	0	OM990854	GGAGGC	GG	AAAGGTGGAGGC	KGGG	I	4	AAA	K
12AUM1061219	GER-N		0	Riepe	53.37852	7.31262	accurate	*Lepus europaeus*	NA	0	0	1	NA	0	0	1	NA	NA	NA	NA	NA	NA	NA	NA	NA
13AUM1061219	GER-N		0	Riepe	53.37852	7.31262	accurate	*Lepus europaeus*	NA	0	0	1	NA	0	0	1	NA	NA	NA	NA	NA	NA	NA	NA	NA
14AUM1061219	GER-N		0	Riepe	53.37852	7.31262	accurate	*Lepus europaeus*	NA	0	0	1	NA	0	0	1	NA	NA	NA	NA	NA	NA	NA	NA	NA
15AUF1061219	GER-N		0	Riepe	53.37852	7.31262	accurate	*Lepus europaeus*	NA	0	0	1	NA	0	0	1	NA	NA	NA	NA	NA	NA	NA	NA	NA
16AUF1061219	GER-N		0	Riepe	53.37852	7.31262	accurate	*Lepus europaeus*	NA	1	0	0	OM939694	1	0	0	**OM990906**	GGAGGC	GG	AAAGGTGGAGGC	KGGG	I	1	AAA	K
17AUF1061219	GER-N		0	Riepe	53.37852	7.31262	accurate	*Lepus europaeus*	NA	0	0	1	NA	0	0	1	NA	NA	NA	NA	NA	NA	NA	NA	NA
18AUF1061219	GER-N		0	Riepe	53.37852	7.31262	accurate	*Lepus europaeus*	NA	0	0	1	NA	0	0	1	NA	NA	NA	NA	NA	NA	NA	NA	NA
19AUF1061219	GER-N		0	Riepe	53.37852	7.31262	accurate	*Lepus europaeus*	NA	0	0	1	NA	0	0	1	NA	NA	NA	NA	NA	NA	NA	NA	NA
20AUF1061219	GER-N		0	Riepe	53.37852	7.31262	accurate	*Lepus europaeus*	NA	0	0	1	NA	0	0	1	NA	NA	NA	NA	NA	NA	NA	NA	NA
21AUM1061219	GER-N		0	Riepe	53.37852	7.31262	accurate	*Lepus europaeus*	NA	0	0	1	NA	0	0	1	NA	NA	NA	NA	NA	NA	NA	NA	NA
22AUF1061219	GER-N		0	Riepe	53.37852	7.31262	accurate	*Lepus europaeus*	NA	0	0	1	NA	0	0	1	NA	NA	NA	NA	NA	NA	NA	NA	NA
23AUM1061219	GER-N		0	Riepe	53.37852	7.31262	accurate	*Lepus europaeus*	NA	1	0	0	**OM939709**	1	0	0	**OM990926**	GGAGGC	GG	AAAGGTGGAGGC	KGGG	I	2	AAA	K
24AUM1061219	GER-N		0	Riepe	53.37852	7.31262	accurate	*Lepus europaeus*	NA	0	0	1	NA	0	0	1	NA	NA	NA	NA	NA	NA	NA	NA	NA
25AUF1061219	GER-N		0	Riepe	53.37852	7.31262	accurate	*Lepus europaeus*	NA	0	0	1	NA	0	0	1	NA	NA	NA	NA	NA	NA	NA	NA	NA
26AUF1061219	GER-N		0	Riepe	53.37852	7.31262	accurate	*Lepus europaeus*	NA	0	0	1	NA	0	0	1	NA	NA	NA	NA	NA	NA	NA	NA	NA
27AUM1061219	GER-N		0	Riepe	53.37852	7.31262	accurate	*Lepus europaeus*	NA	1	0	0	OM939693	1	0	0	**OM990936**	GGAGGC	GG	AAAGGTGGAGGC	KGGG	I	4	AAA	K
28AUM2061219	GER-N		1	Riepe	53.37852	7.31262	accurate	*Lepus europaeus*	NA	0	0	1	NA	0	1	0	NA	NA	NA	NA	NA	NA	NA	NA	NA
29AUF1061219	GER-N		0	Riepe	53.37852	7.31262	accurate	*Lepus europaeus*	NA	1	0	0	OM939698	1	0	0	**OM990938**	GGAGGC	GG	AAAGGTGGAGGC	KGGG	I	5	AAA	K
30AUM1061219	GER-N		0	Riepe	53.37852	7.31262	accurate	*Lepus europaeus*	NA	0	0	1	NA	0	1	0	NA	NA	NA	NA	NA	NA	NA	NA	NA
31AUF1061219	GER-N		0	Riepe	53.37852	7.31262	accurate	*Lepus europaeus*	NA	0	1	0	NA	0	1	0	NA	NA	NA	NA	NA	NA	NA	NA	NA
32AUF1061219	GER-N		0	Riepe	53.37852	7.31262	accurate	*Lepus europaeus*	NA	0	0	1	NA	0	0	1	NA	NA	NA	NA	NA	NA	NA	NA	NA
33AUM1061219	GER-N		0	Riepe	53.37852	7.31262	accurate	*Lepus europaeus*	NA	0	1	0	NA	0	1	0	NA	NA	NA	NA	NA	NA	NA	NA	NA
34AUF1061219	GER-N		0	Riepe	53.37852	7.31262	accurate	*Lepus europaeus*	NA	0	0	1	NA	0	0	1	NA	NA	NA	NA	NA	NA	NA	NA	NA
35AUF1061219	GER-N		0	Riepe	53.37852	7.31262	accurate	*Lepus europaeus*	NA	0	1	0	NA	0	0	1	NA	NA	NA	NA	NA	NA	NA	NA	NA
36AUM1061219	GER-N		0	Riepe	53.37852	7.31262	accurate	*Lepus europaeus*	NA	0	0	1	NA	0	0	1	NA	NA	NA	NA	NA	NA	NA	NA	NA
37AUF1061219	GER-N		0	Riepe	53.37852	7.31262	accurate	*Lepus europaeus*	NA	0	0	1	NA	0	0	1	NA	NA	NA	NA	NA	NA	NA	NA	NA
38AUM1061219	GER-N		0	Riepe	53.37852	7.31262	accurate	*Lepus europaeus*	NA	1	0	0	OM939693	1	0	0	**OM990952**	GGAGGC	GG	AAAGGTGGAGGC	KGGG	I	2	AAA	K
39AUF1061219	GER-N		0	Riepe	53.37852	7.31262	accurate	*Lepus europaeus*	NA	0	0	1	NA	0	0	1	NA	NA	NA	NA	NA	NA	NA	NA	NA
40AUF1061219	GER-N		0	Riepe	53.37852	7.31262	accurate	*Lepus europaeus*	NA	0	1	0	NA	0	1	0	NA	NA	NA	NA	NA	NA	NA	NA	NA
41AUF1061219	GER-N		0	Riepe	53.37852	7.31262	accurate	*Lepus europaeus*	NA	0	1	0	NA	0	1	0	NA	NA	NA	NA	NA	NA	NA	NA	NA
42AUM1061219	GER-N		0	Riepe	53.37852	7.31262	accurate	*Lepus europaeus*	NA	1	0	0	OM939694	1	0	0	**OM990956**	GGAGGC	GG	AAAGGTGGAGGC	KGGG	I	5	AAA	K
43AUF1061219	GER-N		0	Riepe	53.37852	7.31262	accurate	*Lepus europaeus*	NA	0	1	0	NA	0	1	0	NA	NA	NA	NA	NA	NA	NA	NA	NA
44AUM2061219	GER-N		1	Riepe	53.37852	7.31262	accurate	*Lepus europaeus*	NA	0	1	0	NA	0	1	0	NA	NA	NA	NA	NA	NA	NA	NA	NA
45AUF1061219	GER-N		0	Riepe	53.37852	7.31262	accurate	*Lepus europaeus*	NA	0	0	1	NA	1	0	0	**OM990961**	GGAGGC	GG	AAAGGTGGAGGC	KGGG	I	3	AAA	K
46AUF1061219	GER-N		0	Riepe	53.37852	7.31262	accurate	*Lepus europaeus*	NA	0	0	1	NA	0	0	1	NA	NA	NA	NA	NA	NA	NA	NA	NA
47AUM1061219	GER-N		0	Riepe	53.37852	7.31262	accurate	*Lepus europaeus*	NA	0	1	0	NA	0	1	0	NA	NA	NA	NA	NA	NA	NA	NA	NA
48AUF1061219	GER-N		0	Riepe	53.37852	7.31262	accurate	*Lepus europaeus*	NA	0	0	1	NA	0	0	1	NA	NA	NA	NA	NA	NA	NA	NA	NA
49AUM1061219	GER-N		0	Riepe	53.37852	7.31262	accurate	*Lepus europaeus*	NA	0	0	1	NA	1	0	0	**OM990967**	NA	NA	AAAGGC	KG	V	3	AAAGGCAAA	KGK
50AUF1061219	GER-N		0	Riepe	53.37852	7.31262	accurate	*Lepus europaeus*	NA	1	0	0	OM939693	1	0	0	**OM990968**	GGAGGC	GG	AAAGGTGGAGGC	KGGG	I	3	AAA	K
51AUM1061219	GER-N		0	Riepe	53.37852	7.31262	accurate	*Lepus europaeus*	NA	0	0	1	NA	0	0	1	NA	NA	NA	NA	NA	NA	NA	NA	NA
52AUM1061219	GER-N		0	Riepe	53.37852	7.31262	accurate	*Lepus europaeus*	NA	0	1	0	NA	0	0	1	NA	NA	NA	NA	NA	NA	NA	NA	NA
53AUM1061219	GER-N		0	Riepe	53.37852	7.31262	accurate	*Lepus europaeus*	NA	0	1	0	NA	0	1	0	NA	NA	NA	NA	NA	NA	NA	NA	NA
54AUM1061219	GER-N		0	Riepe	53.37852	7.31262	accurate	*Lepus europaeus*	NA	0	0	1	NA	1	0	0	**OM990972**	GGAGGC	GG	AAAGGTGGAGGC	KGGG	NA	2	AAAAGTGGAGGCAAA	KSGGK

55AUM1061219	GER-N		0	Riepe	53.37852	7.31262	accurate	*Lepus europaeus*	NA	0	0	1	NA	0	0	1	NA	NA	NA	NA	NA	NA	NA	NA	NA
56AUM1061219	GER-N		0	Riepe	53.37852	7.31262	accurate	*Lepus europaeus*	NA	0	0	1	NA	0	0	1	NA	NA	NA	NA	NA	NA	NA	NA	NA
57AUM1061219	GER-N		0	Riepe	53.37852	7.31262	accurate	*Lepus europaeus*	NA	0	0	1	NA	0	0	1	NA	NA	NA	NA	NA	NA	NA	NA	NA
58AUF1061219	GER-N		0	Riepe	53.37852	7.31262	accurate	*Lepus europaeus*	NA	0	0	1	NA	1	0	0	**OM990976**	GGAGGC	GG	AAAGGTGGAGGC	KGGG	I	3	AAA	K
59AUF1061219	GER-N		0	Riepe	53.37852	7.31262	accurate	*Lepus europaeus*	NA	1	0	0	OM939694	1	0	0	**OM990978**	GGAGTCAAAGGTGGAGTC	GVKGGV	AAAGGTGGAGGC	KGGG	NA	1	AAA	K
60AUM1061219	GER-N		0	Riepe	53.37852	7.31262	accurate	*Lepus europaeus*	NA	0	0	1	NA	0	0	1	NA	NA	NA	NA	NA	NA	NA	NA	NA
61AUF1061219	GER-N		0	Riepe	53.37852	7.31262	accurate	*Lepus europaeus*	NA	1	0	0	OM939694	1	0	0	**OM990980**	GGAGTC	GV	AAAGGTGGAGGC	KGGG	I	2	AAA	K
01IZF1071219	GER-N		0	Nienborstel	54.13250	9.60602	accurate	*Lepus europaeus*	NA	0	0	1	NA	0	0	1	NA	NA	NA	NA	NA	NA	NA	NA	NA
02IZF1071219	GER-N		0	Nienborstel	54.13250	9.60602	accurate	*Lepus europaeus*	NA	0	1	0	NA	0	1	0	NA	NA	NA	NA	NA	NA	NA	NA	NA
03IZM1071219	GER-N		0	Nienborstel	54.13250	9.60602	accurate	*Lepus europaeus*	NA	0	0	1	NA	0	0	1	NA	NA	NA	NA	NA	NA	NA	NA	NA
04IZF1071219	GER-N		0	Nienborstel	54.13250	9.60602	accurate	*Lepus europaeus*	NA	0	1	0	NA	1	0	0	**OM990864**	GGAGGC	GG	AAAGGTGGAGGC	KGGG	I	3	AAA	K
05IZF1071219	GER-N		0	Nienborstel	54.13250	9.60602	accurate	*Lepus europaeus*	NA	0	0	1	NA	0	0	1	NA	NA	NA	NA	NA	NA	NA	NA	NA
06IZF1071219	GER-N		0	Nienborstel	54.13250	9.60602	accurate	*Lepus europaeus*	NA	1	0	0	OM939694	1	0	0	**OM990872**	GGAGGC	GG	AAAGGTGGAGGC	KGGG	NA	2	(AAAGGAGGC)2AAA	(KGG)2K
07IZF1071219	GER-N		0	Nienborstel	54.13250	9.60602	accurate	*Lepus europaeus*	NA	0	0	1	NA	1	0	0	**OM990877**	GGAGGC	GG	AAAGGAGGC	KGG	IV	3	AAA	K
08IZF1071219	GER-N		0	Nienborstel	54.13250	9.60602	accurate	*Lepus europaeus*	NA	0	1	0	NA	0	1	0	NA	NA	NA	NA	NA	NA	NA	NA	NA
09IZF1071219	GER-N		0	Nienborstel	54.13250	9.60602	accurate	*Lepus europaeus*	NA	1	0	0	OM939694	1	0	0	OM990877	GGAGGC	GG	AAAGGAGGC	KGG	IV	3	AAA	K
10IZM1071219	GER-N		0	Nienborstel	54.13250	9.60602	accurate	*Lepus europaeus*	NA	0	0	1	NA	0	0	1	NA	NA	NA	NA	NA	NA	NA	NA	NA
11IZF1071219	GER-N		0	Nienborstel	54.13250	9.60602	accurate	*Lepus europaeus*	NA	1	0	0	OM939693	1	0	0	**OM990889**	GGAGGC	GG	AAAGGTGGAGGC	KGGG	I	2	AAA	K
12IZF1071219	GER-N		0	Nienborstel	54.13250	9.60602	accurate	*Lepus europaeus*	NA	0	0	1	NA	0	0	1	NA	NA	NA	NA	NA	NA	NA	NA	NA
13IZM1071219	GER-N		0	Nienborstel	54.13250	9.60602	accurate	*Lepus europaeus*	NA	0	1	0	NA	0	0	1	NA	NA	NA	NA	NA	NA	NA	NA	NA
14IZF1071219	GER-N		0	Nienborstel	54.13250	9.60602	accurate	*Lepus europaeus*	NA	1	0	0	OM939694	1	0	0	**OM990899**	AGAGGC	RG	AAAGGTGGAGGC	KGGG	I	4	AAAGGCAAA	KGK
15IZF1071219	GER-N		0	Nienborstel	54.13250	9.60602	accurate	*Lepus europaeus*	NA	1	0	0	OM939693	1	0	0	**OM990903**	GGAGGC	GG	AAAGGTGGAGGC	KGGG	I	2	AAA	K
16IZM1071219	GER-N		0	Nienborstel	54.13250	9.60602	accurate	*Lepus europaeus*	NA	1	0	0	OM939694	1	0	0	OM990864	GGAGGC	GG	AAAGGTGGAGGC	KGGG	I	3	AAA	K
17IZF1071219	GER-N		0	Nienborstel	54.13250	9.60602	accurate	*Lepus europaeus*	NA	1	0	0	OM939694	1	0	0	**OM990913**	GGAGGC	GG	AAAGGTGGAGGC	KGGG	I	5	AAA	K
18IZF1071219	GER-N		0	Nienborstel	54.13250	9.60602	accurate	*Lepus europaeus*	NA	0	0	1	NA	0	0	1	NA	NA	NA	NA	NA	NA	NA	NA	NA
19IZM1071219	GER-N		0	Nienborstel	54.13250	9.60602	accurate	*Lepus europaeus*	NA	0	0	1	NA	0	0	1	NA	NA	NA	NA	NA	NA	NA	NA	NA
20IZM1071219	GER-N		0	Nienborstel	54.13250	9.60602	accurate	*Lepus europaeus*	NA	0	1	0	NA	0	1	0	NA	NA	NA	NA	NA	NA	NA	NA	NA
21IZF1071219	GER-N		0	Jahrsdorf	54.06483	9.63205	accurate	*Lepus europaeus*	NA	0	1	0	NA	0	1	0	NA	NA	NA	NA	NA	NA	NA	NA	NA
22IZF1071219	GER-N		0	Jahrsdorf	54.06483	9.63205	accurate	*Lepus europaeus*	NA	1	0	0	OM939694	1	0	0	**OM990924**	GGAGGC	GG	AAAGGTGGAGGC	KGGG	I	3	AAA	K
23IZF1071219	GER-N		0	Jahrsdorf	54.06483	9.63205	accurate	*Lepus europaeus*	NA	0	0	1	NA	0	0	1	NA	NA	NA	NA	NA	NA	NA	NA	NA
24IZF2071219	GER-N		1	Jahrsdorf	54.06483	9.63205	accurate	*Lepus europaeus*	NA	1	0	0	OM939693	1	0	0	OM990903	GGAGGC	GG	AAAGGTGGAGGC	KGGG	I	2	AAA	K
25IZF1071219	GER-N		0	Jahrsdorf	54.06483	9.63205	accurate	*Lepus europaeus*	NA	0	0	1	NA	0	0	1	NA	NA	NA	NA	NA	NA	NA	NA	NA
26IZM1071219	GER-N		0	Jahrsdorf	54.06483	9.63205	accurate	*Lepus europaeus*	NA	1	0	0	**OM939703**	1	0	0	**OM990935**	GGAGGC	GG	AAAGGTGGAGGC	KGGG	I	1	AAA	K
27IZF1071219	GER-N		0	Jahrsdorf	54.06483	9.63205	accurate	*Lepus europaeus*	NA	0	0	1	NA	0	0	1	NA	NA	NA	NA	NA	NA	NA	NA	NA
28IZF1071219	GER-N		0	Jahrsdorf	54.06483	9.63205	accurate	*Lepus europaeus*	NA	1	0	0	OM939693	1	0	0	**OM990937**	GGAGGC	GG	AAAGGTGGAGGC	KGGG	I	4	AAA	K
29IZM1071219	GER-N		0	Jahrsdorf	54.06483	9.63205	accurate	*Lepus europaeus*	NA	0	1	0	NA	0	1	0	NA	NA	NA	NA	NA	NA	NA	NA	NA
30IZF1071219	GER-N		0	Jahrsdorf	54.06483	9.63205	accurate	*Lepus europaeus*	NA	1	0	0	OM939694	1	0	0	**OM990940**	GGAGGC	GG	AAAGGTGGAGGC	KGGG	I	3	AAA	K
32IZM1071219	GER-N		0	Nienborstel	54.13250	9.60602	accurate	*Lepus europaeus*	NA	0	1	0	NA	1	0	0	**OM990943**	GGAGGC	GG	AAAGGTGGAGGC	KGGG	NA	1	AAAGGAAGC(AAAGGAGGC)2AAA	KGS(KGG)2K
33IZM1071219	GER-N		0	Nienborstel	54.13250	9.60602	accurate	*Lepus europaeus*	NA	0	1	0	NA	0	1	0	NA	NA	NA	NA	NA	NA	NA	NA	NA
34IZM1071219	GER-N		0	Nienborstel	54.13250	9.60602	accurate	*Lepus europaeus*	NA	0	0	1	NA	0	0	1	NA	NA	NA	NA	NA	NA	NA	NA	NA
35IZM1071219	GER-N		0	Nienborstel	54.13250	9.60602	accurate	*Lepus europaeus*	NA	0	0	1	NA	0	0	1	NA	NA	NA	NA	NA	NA	NA	NA	NA
36IZF1071219	GER-N		0	Nienborstel	54.13250	9.60602	accurate	*Lepus europaeus*	NA	1	0	0	OM939703	1	0	0	**OM990950**	GGAGGC	GG	AAAGGTGGAGGC	KGGG	I	2	AAA	K
37IZM1071219	GER-N		0	Nienborstel	54.13250	9.60602	accurate	*Lepus europaeus*	NA	0	0	1	NA	0	0	1	NA	NA	NA	NA	NA	NA	NA	NA	NA
38IZM1071219	GER-N		0	Nienborstel	54.13250	9.60602	accurate	*Lepus europaeus*	NA	0	1	0	NA	0	1	0	NA	NA	NA	NA	NA	NA	NA	NA	NA
39IZF1071219	GER-N		0	Nienborstel	54.13250	9.60602	accurate	*Lepus europaeus*	NA	1	0	0	OM939693	1	0	0	**OM990954**	GGAGGC	GG	NA	NA	NA	NA	AAA	K
40IZF1071219	GER-N		0	Nienborstel	54.13250	9.60602	accurate	*Lepus europaeus*	NA	0	1	0	NA	0	0	1	NA	NA	NA	NA	NA	NA	NA	NA	NA
41IZF1071219	GER-N		0	Nienborstel	54.13250	9.60602	accurate	*Lepus europaeus*	NA	0	0	1	NA	0	0	1	NA	NA	NA	NA	NA	NA	NA	NA	NA
42IZF1071219	GER-N		0	Nienborstel	54.13250	9.60602	accurate	*Lepus europaeus*	NA	0	0	1	NA	1	0	0	**OM990958**	GGAGGC	GG	AAACAGGGAGGC	KQGG	III	1	AAA	K
43IZF1071219	GER-N		0	Nienborstel	54.13250	9.60602	accurate	*Lepus europaeus*	NA	0	1	0	NA	0	1	0	NA	NA	NA	NA	NA	NA	NA	NA	NA
44IZF1071219	GER-N		0	Nienborstel	54.13250	9.60602	accurate	*Lepus europaeus*	NA	1	0	0	OM939693	1	0	0	**OM990960**	GGAGGC	GG	AAACAGGGAGGC	KQGG	III	2	AAA	K
45IZF1071219	GER-N		0	Nienborstel	54.13250	9.60602	accurate	*Lepus europaeus*	NA	0	0	1	NA	0	0	1	NA	NA	NA	NA	NA	NA	NA	NA	NA
01PEM1181219	GER-CW		0	Bierbergen	52.23260	10.09678	accurate	*Lepus europaeus*	NA	0	0	1	NA	0	0	1	NA	NA	NA	NA	NA	NA	NA	NA	NA
02PEF1181219	GER-CW		0	Bierbergen	52.23260	10.09678	accurate	*Lepus europaeus*	NA	0	0	1	NA	0	0	1	NA	NA	NA	NA	NA	NA	NA	NA	NA
03PEF1181219	GER-CW		1	Bierbergen	52.23260	10.09678	accurate	*Lepus europaeus*	NA	1	0	0	**OM939723**	1	0	0	**OM990862**	GGAGGC	GG	AAAGGTGGAGGC	KGGG	I	2	AAA	K
04PEM1181219	GER-CW		0	Bierbergen	52.23260	10.09678	accurate	*Lepus europaeus*	NA	0	1	0	NA	0	0	1	NA	NA	NA	NA	NA	NA	NA	NA	NA
05PEM1181219	GER-CW		0	Bierbergen	52.23260	10.09678	accurate	*Lepus europaeus*	NA	0	0	1	NA	0	0	1	NA	NA	NA	NA	NA	NA	NA	NA	NA
06PEF1181219	GER-CW		0	Bierbergen	52.23260	10.09678	accurate	*Lepus europaeus*	NA	1	0	0	**OM939716**	1	0	0	**OM990873**	AAAGGCGGAGGC	KGGG	AAAGGC	KG	NA	2	AAAGGCAAA	KGK
07PEM1181219	GER-CW		0	Bierbergen	52.23260	10.09678	accurate	*Lepus europaeus*	NA	0	0	1	NA	0	0	1	NA	NA	NA	NA	NA	NA	NA	NA	NA
08PEF1181219	GER-CW		0	Bierbergen	52.23260	10.09678	accurate	*Lepus europaeus*	NA	0	0	1	NA	0	0	1	NA	NA	NA	NA	NA	NA	NA	NA	NA
09PEM1181219	GER-CW		0	Bierbergen	52.23260	10.09678	accurate	*Lepus europaeus*	NA	0	0	1	NA	0	0	1	NA	NA	NA	NA	NA	NA	NA	NA	NA
10PEF1181219	GER-CW		0	Bierbergen	52.23260	10.09678	accurate	*Lepus europaeus*	NA	0	0	1	NA	0	0	1	NA	NA	NA	NA	NA	NA	NA	NA	NA
11PEF1181219	GER-CW		0	Bierbergen	52.23260	10.09678	accurate	*Lepus europaeus*	NA	1	0	0	OM939693	1	0	0	**OM990890**	AAAGGCGGAGGC	KGGG	AAAGGC	KG	NA	4	AAA	K
12PEF1181219	GER-CW		0	Bierbergen	52.23260	10.09678	accurate	*Lepus europaeus*	NA	0	0	1	NA	1	0	0	**OM990892**	AAAGGCGGAGGC	KGGG	AAAGGC	KG	NA	4	AAA	K
13PEF1181219	GER-CW		0	Bierbergen	52.23260	10.09678	accurate	*Lepus europaeus*	NA	0	0	1	NA	0	0	1	NA	NA	NA	NA	NA	NA	NA	NA	NA
14PEM1181219	GER-CW		0	Bierbergen	52.23260	10.09678	accurate	*Lepus europaeus*	NA	0	0	1	NA	0	0	1	NA	NA	NA	NA	NA	NA	NA	NA	NA
15PEF1181219	GER-CW		0	Bierbergen	52.23260	10.09678	accurate	*Lepus europaeus*	NA	0	0	1	NA	0	0	1	NA	NA	NA	NA	NA	NA	NA	NA	NA
16PEF1181219	GER-CW		0	Bierbergen	52.23260	10.09678	accurate	*Lepus europaeus*	NA	0	0	1	NA	0	0	1	NA	NA	NA	NA	NA	NA	NA	NA	NA
17PEF1181219	GER-CW		0	Bierbergen	52.23260	10.09678	accurate	*Lepus europaeus*	NA	1	0	0	OM939693	1	0	0	**OM990915**	AAAGGCGGAGGC	KGGG	AAAGGC	KG	NA	2	AAAGGCAAA	KGK
18PEM1181219	GER-CW		0	Bierbergen	52.23260	10.09678	accurate	*Lepus europaeus*	NA	0	1	0	NA	0	0	1	NA	NA	NA	NA	NA	NA	NA	NA	NA
19PEF1181219	GER-CW		0	Bierbergen	52.23260	10.09678	accurate	*Lepus europaeus*	NA	0	0	1	NA	0	0	1	NA	NA	NA	NA	NA	NA	NA	NA	NA
20PEM1181219	GER-CW		0	Bierbergen	52.23260	10.09678	accurate	*Lepus europaeus*	NA	0	0	1	NA	0	0	1	NA	NA	NA	NA	NA	NA	NA	NA	NA
21PEF1181219	GER-CW		0	Bierbergen	52.23260	10.09678	accurate	*Lepus europaeus*	NA	0	0	1	NA	0	0	1	NA	NA	NA	NA	NA	NA	NA	NA	NA
22PEF1181219	GER-CW		0	Bierbergen	52.23260	10.09678	accurate	*Lepus europaeus*	NA	1	0	0	OM939693	1	0	0	**OM990925**	AAAGGCGGAGGC	KGGG	AAAGGC	KG	NA	2	AAA	K
23PEF1181219	GER-CW		0	Bierbergen	52.23260	10.09678	accurate	*Lepus europaeus*	NA	0	0	1	NA	1	0	0	OM990925	AAAGGCGGAGGC	KGGG	AAAGGC	KG	NA	2	AAA	K
24PEF1181219	GER-CW		0	Bierbergen	52.23260	10.09678	accurate	*Lepus europaeus*	NA	1	0	0	OM939693	1	0	0	**OM990932**	NA	NA	AAAGGCGGAGGC	KGGG	I	3	AAAGGCAAA	KGK
01GÖM1191219	GER-CW		0	Reinshof	51.49757	9.93135	accurate	*Lepus europaeus*	NA	0	0	1	NA	0	0	1	NA	NA	NA	NA	NA	NA	NA	NA	NA
02GÖM1191219	GER-CW		0	Reinshof	51.49757	9.93135	accurate	*Lepus europaeus*	NA	0	1	0	NA	0	1	0	NA	NA	NA	NA	NA	NA	NA	NA	NA
03GÖM1191219	GER-CW		0	Reinshof	51.49757	9.93135	accurate	*Lepus europaeus*	NA	0	0	1	NA	0	0	1	NA	NA	NA	NA	NA	NA	NA	NA	NA
04GÖM1191219	GER-CW		0	Reinshof	51.49757	9.93135	accurate	*Lepus europaeus*	NA	0	0	1	NA	0	0	1	NA	NA	NA	NA	NA	NA	NA	NA	NA
05GÖF1191219	GER-CW		0	Reinshof	51.49757	9.93135	accurate	*Lepus europaeus*	NA	0	0	1	NA	0	0	1	NA	NA	NA	NA	NA	NA	NA	NA	NA
06GÖM1191219	GER-CW		0	Reinshof	51.49757	9.93135	accurate	*Lepus europaeus*	NA	0	0	1	NA	0	0	1	NA	NA	NA	NA	NA	NA	NA	NA	NA
07GÖM1191219	GER-CW		0	Reinshof	51.49757	9.93135	accurate	*Lepus europaeus*	NA	0	0	1	NA	0	0	1	NA	NA	NA	NA	NA	NA	NA	NA	NA
08GÖF1191219	GER-CW		0	Reinshof	51.49757	9.93135	accurate	*Lepus europaeus*	NA	0	0	1	NA	0	0	1	NA	NA	NA	NA	NA	NA	NA	NA	NA
09GÖM1191219	GER-CW		0	Reinshof	51.49757	9.93135	accurate	*Lepus europaeus*	NA	0	0	1	NA	0	0	1	NA	NA	NA	NA	NA	NA	NA	NA	NA
10GÖF1191219	GER-CW		0	Reinshof	51.49757	9.93135	accurate	*Lepus europaeus*	NA	0	0	1	NA	0	0	1	NA	NA	NA	NA	NA	NA	NA	NA	NA
11GÖF1191219	GER-CW		0	Reinshof	51.49757	9.93135	accurate	*Lepus europaeus*	NA	0	1	0	NA	0	0	1	NA	NA	NA	NA	NA	NA	NA	NA	NA
12GÖF1191219	GER-CW		0	Reinshof	51.49757	9.93135	accurate	*Lepus europaeus*	NA	0	1	0	NA	0	0	1	NA	NA	NA	NA	NA	NA	NA	NA	NA
13GÖF1191219	GER-CW		0	Reinshof	51.49757	9.93135	accurate	*Lepus europaeus*	NA	0	0	1	NA	0	0	1	NA	NA	NA	NA	NA	NA	NA	NA	NA
14GÖM1191219	GER-CW		0	Reinshof	51.49757	9.93135	accurate	*Lepus europaeus*	NA	0	0	1	NA	0	0	1	NA	NA	NA	NA	NA	NA	NA	NA	NA
15GÖM1301219	GER-CW		0	Parensen	51.58757	9.92188	accurate	*Lepus europaeus*	NA	0	0	1	NA	0	0	1	NA	NA	NA	NA	NA	NA	NA	NA	NA
16GÖM1301219	GER-CW		0	Parensen	51.58757	9.92188	accurate	*Lepus europaeus*	NA	1	0	0	OM939694	1	0	0	**OM990911**	GGAGGC	GG	AAAGGTGGAGGC	KGGG	I	2	AAA	K
17GÖF1301219	GER-CW		0	Parensen	51.58757	9.92188	accurate	*Lepus europaeus*	NA	0	0	1	NA	1	0	0	OM990911	GGAGGC	GG	AAAGGTGGAGGC	KGGG	I	2	AAA	K
18GÖM1301219	GER-CW		0	Parensen	51.58757	9.92188	accurate	*Lepus europaeus*	NA	0	1	0	NA	0	1	0	NA	NA	NA	NA	NA	NA	NA	NA	NA
19GÖM1301219	GER-CW		0	Parensen	51.58757	9.92188	accurate	*Lepus europaeus*	NA	0	0	1	NA	0	0	1	NA	NA	NA	NA	NA	NA	NA	NA	NA
20GÖM1301219	GER-CW		0	Parensen	51.58757	9.92188	accurate	*Lepus europaeus*	NA	1	0	0	**OM939714**	1	0	0	**OM990918**	GGAGGC	GG	AAAGGTGGAGGC	KGGG	I	2	AAA	K
21GÖF1301219	GER-CW		0	Parensen	51.58757	9.92188	accurate	*Lepus europaeus*	NA	0	0	1	NA	1	0	0	**OM990921**	GGAGGC	GG	AAAGGTGGAGGC	KGGG	I	1	AAA	K
22GÖF1301219	GER-CW		0	Parensen	51.58757	9.92188	accurate	*Lepus europaeus*	NA	1	0	0	OM939693	1	0	0	**OM990923**	GGAGGC	GG	AAAGGTGGAGGC	KGGG	I	3	AAA	K
23GÖF1301219	GER-CW		0	Parensen	51.58757	9.92188	accurate	*Lepus europaeus*	NA	0	0	1	NA	1	0	0	OM990911	GGAGGC	GG	AAAGGTGGAGGC	KGGG	I	2	AAA	K
24GÖM1301219	GER-CW		0	Parensen	51.58757	9.92188	accurate	*Lepus europaeus*	NA	0	1	0	NA	0	1	0	NA	NA	NA	NA	NA	NA	NA	NA	NA
25GÖM1301219	GER-CW		0	Parensen	51.58757	9.92188	accurate	*Lepus europaeus*	NA	0	0	1	NA	0	0	1	NA	NA	NA	NA	NA	NA	NA	NA	NA
26GÖM1301219	GER-CW		0	Parensen	51.58757	9.92188	accurate	*Lepus europaeus*	NA	1	0	0	OM939693	1	0	0	OM990923	GGAGGC	GG	AAAGGTGGAGGC	KGGG	I	3	AAA	K
27GÖF1301219	GER-CW		0	Parensen	51.58757	9.92188	accurate	*Lepus europaeus*	NA	0	1	0	NA	0	0	1	NA	NA	NA	NA	NA	NA	NA	NA	NA
01BWFX051019	GER-S		unknown	Bad Krozingen-Biengen	47.93977	7.68912	estimated	*Lepus europaeus*	**ON089362**	1	0	0	**OM939696**	1	0	0	**OM990855**	GGAGGC	GG	AAAGGTGGAGGC	KGGG	I	3	AAA	K
02BWFX051019	GER-S		unknown	Bad Krozingen-Biengen	47.93977	7.68912	estimated	*Lepus europaeus*	**ON089366**	1	0	0	OM939696	1	0	0	OM990855	GGAGGC	GG	AAAGGTGGAGGC	KGGG	I	3	AAA	K

03BWFX051019	GER-S		unknown	Bad Krozingen-Biengen	47.93977	7.68912	estimated	*Lepus europaeus*	**ON089371**	1	0	0	**OM939708**	1	0	0	OM990855	GGAGGC	GG	AAAGGTGGAGGC	KGGG	I	3	AAA	K
04BWFX051019	GER-S		unknown	Bad Krozingen-Biengen	47.93977	7.68912	estimated	*Lepus europaeus*	**ON089374**	0	1	0	NA	0	0	1	NA	NA	NA	NA	NA	NA	NA	NA	NA
05BWFX051019	GER-S		unknown	Bad Krozingen-Biengen	47.93977	7.68912	estimated	*Lepus europaeus*	**ON089377**	1	0	0	OM939696	1	0	0	OM990855	GGAGGC	GG	AAAGGTGGAGGC	KGGG	I	3	AAA	K
06BWFX051019	GER-S		unknown	Bad Krozingen-Biengen	47.93977	7.68912	estimated	*Lepus europaeus*	**ON089382**	0	0	1	NA	1	0	0	OM990855	GGAGGC	GG	AAAGGTGGAGGC	KGGG	I	3	AAA	K
07BWFX051019	GER-S		unknown	Bad Krozingen-Biengen	47.93977	7.68912	estimated	*Lepus europaeus*	**ON089384**	1	0	0	OM939696	1	0	0	**OM990875**	GGAGGC	GG	AAAGGTGGAGGC	KGGG	I	6	AAA	K
08BWFX051019	GER-S		unknown	Bad Krozingen-Biengen	47.93977	7.68912	estimated	*Lepus europaeus*	**ON089387**	0	1	0	NA	0	1	0	NA	NA	NA	NA	NA	NA	NA	NA	NA
09BWMX051019	GER-S		unknown	Bad Krozingen-Biengen	47.93977	7.68912	estimated	*Lepus europaeus*	**ON089390**	0	0	1	NA	0	1	0	NA	NA	NA	NA	NA	NA	NA	NA	NA
10BWMX051019	GER-S		unknown	Bad Krozingen-Biengen	47.93977	7.68912	estimated	*Lepus europaeus*	**ON089392**	1	0	0	**OM939712**	1	0	0	**OM990884**	GGAGGC	GG	AAAGGTGGAGGC	KGGG	I	5	AAA	K
11BWFX051019	GER-S		unknown	Bad Krozingen-Biengen	47.93977	7.68912	estimated	*Lepus europaeus*	**ON089396**	1	0	0	**OM939713**	1	0	0	OM990884	GGAGGC	GG	AAAGGTGGAGGC	KGGG	I	5	AAA	K
12BWMX111119	GER-S		unknown	Staufen II Granern	47.87089	7.71939	estimated	*Lepus europaeus*	**ON089398**	0	0	1	NA	0	0	1	NA	NA	NA	NA	NA	NA	NA	NA	NA
13BWMX251119	GER-S		unknown	Tunsel Ost	47.90128	7.67418	estimated	*Lepus europaeus*	**ON089401**	0	0	1	NA	0	0	1	NA	NA	NA	NA	NA	NA	NA	NA	NA
14BWFX011219	GER-S		unknown	Tunsel Ost	47.90128	7.67418	estimated	*Lepus europaeus*	**ON089407**	0	0	1	NA	0	0	1	NA	NA	NA	NA	NA	NA	NA	NA	NA
15BWMX121219	GER-S		unknown	Tunsel Ost	47.90128	7.67418	estimated	*Lepus europaeus*	**ON089410**	1	0	0	OM939693	1	0	0	**OM990900**	GGAGGC	GG	AAAGGTGGAGGC	KGGG	I	3	AAA	K
16BWMX121219	GER-S		unknown	Tunsel Ost	47.90128	7.67418	estimated	*Lepus europaeus*	**ON089415**	1	0	0	OM939694	1	0	0	**OM990907**	GGAGGC	GG	AAAGGTGGAGGC	KGGG	I	3	AAA	K
17BWMX281219	GER-S		unknown	Tunsel Ost	47.90128	7.67418	estimated	*Lepus europaeus*	**ON089420**	0	1	0	NA	0	1	0	NA	NA	NA	NA	NA	NA	NA	NA	NA
18BWFX281219	GER-S		unknown	Tunsel Ost	47.90128	7.67418	estimated	*Lepus europaeus*	**ON089423**	0	1	0	NA	0	0	1	NA	NA	NA	NA	NA	NA	NA	NA	NA
19BWMX281219	GER-S		unknown	Tunsel Ost	47.90128	7.67418	estimated	*Lepus europaeus*	**ON089425**	0	0	1	NA	0	0	1	NA	NA	NA	NA	NA	NA	NA	NA	NA
20BWMX281219	GER-S		unknown	Tunsel Ost	47.90128	7.67418	estimated	*Lepus europaeus*	**ON089426**	0	0	1	NA	0	0	1	NA	NA	NA	NA	NA	NA	NA	NA	NA
21BWFX281219	GER-S		unknown	Tunsel Ost	47.90128	7.67418	estimated	*Lepus europaeus*	**ON089429**	0	0	1	NA	1	0	0	**OM990920**	GGAGGC	GG	AAAGGTGGAGGC	KGGG	I	2	AAA	K
22BWMX281219	GER-S		unknown	Tunsel Ost	47.90128	7.67418	estimated	*Lepus europaeus*	**ON089432**	0	0	1	NA	0	0	1	NA	NA	NA	NA	NA	NA	NA	NA	NA
23BWFX281219	GER-S		unknown	Tunsel Ost	47.90128	7.67418	estimated	*Lepus europaeus*	**ON089434**	0	1	0	NA	1	0	0	**OM990928**	GGAGGC	GG	AAAGGTGGAGGC	KGGG	I	2	AAA	K
24BWMX281219	GER-S		unknown	Tunsel Ost	47.90128	7.67418	estimated	*Lepus europaeus*	**ON089436**	1	0	0	OM939693	1	0	0	**OM990930**	GGAGGC	GG	AAAGGTGGAGGC	KGGG	I	3	AAA	K
01CPMX081119	GER-N		unknown	Hochelsten	52.77003	8.09182	estimated	*Lepus europaeus*	NA	0	0	1	NA	0	0	1	NA	NA	NA	NA	NA	NA	NA	NA	NA
02CPFX081119	GER-N		unknown	Hochelsten	52.77003	8.09182	estimated	*Lepus europaeus*	**ON089367**	0	0	1	NA	0	1	0	NA	NA	NA	NA	NA	NA	NA	NA	NA
03CPFX081119	GER-N		unknown	Hochelsten	52.77003	8.09182	estimated	*Lepus europaeus*	**ON089372**	0	0	1	NA	0	1	0	NA	NA	NA	NA	NA	NA	NA	NA	NA
04CPMX081119	GER-N		unknown	Hochelsten	52.77003	8.09182	estimated	*Lepus europaeus*	ON089399	1	0	0	OM939694	1	0	0	**OM990863**	GGAGGC	GG	AAAGGTGGAGGC	KGGG	NA	1	(AAAGGAGGC)2AAA	(KGG)2K
05CPFX081119	GER-N		unknown	Hochelsten	52.77003	8.09182	estimated	*Lepus europaeus*	**ON089378**	0	1	0	NA	1	0	0	OM990863	GGAGGC	GG	AAAGGTGGAGGC	KGGG	NA	1	(AAAGGAGGC)2AAA	(KGG)2K
06CPFX081119	GER-N		unknown	Hochelsten	52.77003	8.09182	estimated	*Lepus europaeus*	**ON089383**	1	0	0	OM939694	1	0	0	**OM990871**	GTAGGC	VG	AAAGGTGGAGGC	KGGG	I	4	AAA	K
07CPFX081119	GER-N		unknown	Hochelsten	52.77003	8.09182	estimated	*Lepus europaeus*	**ON089385**	1	0	0	OM939694	1	0	0	**OM990876**	GGAGGC	GG	AAAGGTGGAGGC	KGGG	I	1	AAA	K
08CPFX081119	GER-N		unknown	Hochelsten	52.77003	8.09182	estimated	*Lepus europaeus*	**ON089388**	0	1	0	NA	0	1	0	NA	NA	NA	NA	NA	NA	NA	NA	NA
09CPFX081119	GER-N		unknown	Hochelsten	52.77003	8.09182	estimated	*Lepus europaeus*	**ON089391**	1	0	0	OM939694	1	0	0	**OM990881**	GGAGGC	GG	AAAGGTGGAGGC	KGGG	I	3	AAA	K
10CPMX091119	GER-N		1	Siehenfelde	52.80346	8.14808	estimated	*Lepus europaeus*	**ON089393**	1	0	0	OM939694	1	0	0	**OM990885**	GGAGGC	GG	AAAGGTGGAGGC	KGGG	I	3	AAA	K
11CPMX091119	GER-N		unknown	Siehenfelde	52.80346	8.14808	estimated	*Lepus europaeus*	**ON089397**	1	0	0	OM939694	1	0	0	**OM990887**	GGAGGC	GG	AAAGGTGGAGGC	KGGG	I	3	AAA	K
12CPFX091119	GER-N		unknown	Siehenfelde	52.80346	8.14808	estimated	*Lepus europaeus*	**ON089399**	0	1	0	NA	0	1	0	NA	NA	NA	NA	NA	NA	NA	NA	NA
13CPFX091119	GER-N		unknown	Siehenfelde	52.80346	8.14808	estimated	*Lepus europaeus*	**ON089403**	1	0	0	OM939694	1	0	0	OM990885	GGAGGC	GG	AAAGGTGGAGGC	KGGG	I	3	AAA	K
14CPFX091119	GER-N		unknown	Siehenfelde	52.80346	8.14808	estimated	*Lepus europaeus*	**ON089408**	1	0	0	OM939694	1	0	0	OM990885	GGAGGC	GG	AAAGGTGGAGGC	KGGG	I	3	AAA	K
15CPFX091119	GER-N		unknown	Siehenfelde	52.80346	8.14808	estimated	*Lepus europaeus*	**ON089411**	0	0	1	NA	1	0	0	OM990885	GGAGGC	GG	AAAGGTGGAGGC	KGGG	I	3	AAA	K
16CPFX091119	GER-N		unknown	Siehenfelde	52.80346	8.14808	estimated	*Lepus europaeus*	**ON089417**	0	1	0	NA	0	1	0	NA	NA	NA	NA	NA	NA	NA	NA	NA
01DAMX081019	GER-CW		unknown	Darmstadt	49.8633	8.6644	estimated	*Oryctolagus cuniculus*	NA	0	0	1	NA	0	0	1	NA	NA	NA	NA	NA	NA	NA	NA	NA
02DAMX111019	GER-CW		unknown	Darmstadt	49.8633	8.6644	estimated	*Oryctolagus cuniculus*	NA	0	0	1	NA	0	0	1	NA	NA	NA	NA	NA	NA	NA	NA	NA
03DAFX231119	GER-CW		unknown	Darmstadt	49.8633	8.6644	estimated	*Oryctolagus cuniculus*	NA	0	0	1	NA	0	0	1	NA	NA	NA	NA	NA	NA	NA	NA	NA
04DAMX160120	GER-CW		unknown	Darmstadt	49.8633	8.6644	estimated	*Oryctolagus cuniculus*	NA	0	0	1	NA	0	0	1	NA	NA	NA	NA	NA	NA	NA	NA	NA
05DAFX050220	GER-CW		unknown	Darmstadt	49.8633	8.6644	estimated	*Oryctolagus cuniculus*	NA	0	0	1	NA	0	0	1	NA	NA	NA	NA	NA	NA	NA	NA	NA
06DAMX090220	GER-CW		unknown	Darmstadt	49.8633	8.6644	estimated	*Oryctolagus cuniculus*	NA	0	0	1	NA	0	0	1	NA	NA	NA	NA	NA	NA	NA	NA	NA
07DAMX160220	GER-CW		unknown	Darmstadt	49.8633	8.6644	estimated	*Oryctolagus cuniculus*	NA	0	0	1	NA	0	0	1	NA	NA	NA	NA	NA	NA	NA	NA	NA
08DAMX160220	GER-CW		unknown	Darmstadt	49.8633	8.6644	estimated	*Oryctolagus cuniculus*	NA	0	0	1	NA	0	0	1	NA	NA	NA	NA	NA	NA	NA	NA	NA
09DAMX240720	GER-CW		unknown	Darmstadt	49.8633	8.6644	estimated	*Oryctolagus cuniculus*	NA	0	0	1	NA	0	0	1	NA	NA	NA	NA	NA	NA	NA	NA	NA
10DAFX270720	GER-CW		unknown	Darmstadt	49.8633	8.6644	estimated	*Oryctolagus cuniculus*	NA	0	0	1	NA	0	0	1	NA	NA	NA	NA	NA	NA	NA	NA	NA
11DAFX170820	GER-CW		unknown	Darmstadt	49.8633	8.6644	estimated	*Oryctolagus cuniculus*	NA	0	0	1	NA	0	0	1	NA	NA	NA	NA	NA	NA	NA	NA	NA
12DAFX150920	GER-CW		unknown	Darmstadt	49.8633	8.6644	estimated	*Oryctolagus cuniculus*	NA	0	0	1	NA	0	0	1	NA	NA	NA	NA	NA	NA	NA	NA	NA
13DAMX200920	GER-CW		unknown	Darmstadt	49.8633	8.6644	estimated	*Oryctolagus cuniculus*	NA	0	0	1	NA	0	0	1	NA	NA	NA	NA	NA	NA	NA	NA	NA
01NRWFX190320	GER-CW		unknown	CVUA-Muenster	51.985558	7.634976	estimated	*Lepus europaeus*	NA	0	0	1	NA	0	0	1	NA	NA	NA	NA	NA	NA	NA	NA	NA
02NRWMX190320	GER-CW		unknown	CVUA-Muenster	51.985558	7.634976	estimated	*Lepus europaeus*	NA	0	0	1	NA	0	0	1	NA	NA	NA	NA	NA	NA	NA	NA	NA
03NRWFX190320	GER-CW		unknown	CVUA-Muenster	51.985558	7.634976	estimated	*Lepus europaeus*	NA	0	0	1	NA	0	0	1	NA	NA	NA	NA	NA	NA	NA	NA	NA
04NRWMX190320	GER-CW		unknown	CVUA-Muenster	51.985558	7.634976	estimated	*Lepus europaeus*	ON089378	1	0	0	OM939693	1	0	0	**OM990865**	GGAGGC	GG	AAACAGGGAGGC	KQGG	III	2	AAA	K
05NRWMX230320	GER-CW		unknown	CVUA-Muenster	51.985558	7.634976	estimated	*Lepus europaeus*	**ON089381**	1	0	0	**OM939701**	1	0	0	**OM990869**	NA	NA	AAAGGC	KG	V	3	(AAAGGT)2AAA	(KG)2K
06NRWFX190320	GER-CW		unknown	CVUA-Muenster	51.985558	7.634976	estimated	*Lepus europaeus*	NA	0	0	1	NA	0	0	1	NA	NA	NA	NA	NA	NA	NA	NA	NA
07NRWMX190320	GER-CW		unknown	CVUA-Muenster	51.985558	7.634976	estimated	*Lepus europaeus*	NA	0	0	1	NA	0	0	1	NA	NA	NA	NA	NA	NA	NA	NA	NA
08NRWMX190320	GER-CW		unknown	CVUA-Muenster	51.985558	7.634976	estimated	*Lepus europaeus*	NA	0	0	1	NA	0	0	1	NA	NA	NA	NA	NA	NA	NA	NA	NA
09NRWFX250320	GER-CW		unknown	CVUA-Muenster	51.985558	7.634976	estimated	*Lepus europaeus*	NA	0	0	1	NA	0	0	1	NA	NA	NA	NA	NA	NA	NA	NA	NA
10NRWMX270320	GER-CW		unknown	CVUA-Muenster	51.985558	7.634976	estimated	*Lepus europaeus*	**ON089395**	1	0	0	OM939693	0	1	0	NA	NA	NA	NA	NA	NA	NA	NA	NA
11NRWMX020420	GER-CW		unknown	CVUA-Muenster	51.985558	7.634976	estimated	*Lepus europaeus*	NA	0	0	1	NA	0	0	1	NA	NA	NA	NA	NA	NA	NA	NA	NA
12NRWMX140420	GER-CW		unknown	CVUA-Muenster	51.985558	7.634976	estimated	*Lepus europaeus*	NA	0	0	1	NA	0	0	1	NA	NA	NA	NA	NA	NA	NA	NA	NA
13NRWMX220420	GER-CW		unknown	CVUA-Muenster	51.985558	7.634976	estimated	*Lepus europaeus*	**ON089405**	1	0	0	OM939694	1	0	0	**OM990896**	NA	NA	NA	NA	NA	NA	NA	NA
14NRWMX220420	GER-CW		unknown	CVUA-Muenster	51.985558	7.634976	estimated	*Lepus europaeus*	NA	0	0	1	NA	0	0	1	NA	NA	NA	NA	NA	NA	NA	NA	NA
15NRWFX220420	GER-CW		unknown	CVUA-Muenster	51.985558	7.634976	estimated	*Lepus europaeus*	**ON089413**	0	0	1	NA	1	0	0	**OM990904**	GGAGGC	GG	AAAGGTGGAGGC	KGGG	I	7	AAA	K
16NRWFX020420	GER-CW		unknown	CVUA-Muenster	51.985558	7.634976	estimated	*Lepus europaeus*	**ON089419**	0	0	1	NA	0	1	0	NA	NA	NA	NA	NA	NA	NA	NA	NA
17NRWMX250320	GER-CW		unknown	CVUA-Muenster	51.985558	7.634976	estimated	*Lepus europaeus*	**ON089422**	1	0	0	OM939693	1	0	0	**OM990914**	GGAGGC	GG	AAAGGTGGAGGC	KGGG	I	3	AAA	K
18NRWFX280420	GER-CW		unknown	CVUA-Muenster	51.985558	7.634976	estimated	*Lepus europaeus*	NA	0	0	1	NA	0	0	1	NA	NA	NA	NA	NA	NA	NA	NA	NA
19NRWFX270420	GER-CW		unknown	CVUA-Muenster	51.985558	7.634976	estimated	*Lepus europaeus*	NA	0	0	1	NA	0	0	1	NA	NA	NA	NA	NA	NA	NA	NA	NA
20NRWFX050520	GER-CW		unknown	CVUA-Muenster	51.985558	7.634976	estimated	*Lepus europaeus*	**ON089428**	1	0	0	OM939694	1	0	0	**OM990919**	NA	NA	AAAGGCAAAGGT	KGKG	NA	2	(AAAGGC)2AAA	(KG)2K
21NRWMX040520	GER-CW		unknown	CVUA-Muenster	51.985558	7.634976	estimated	*Lepus europaeus*	NA	0	0	1	NA	0	0	1	NA	NA	NA	NA	NA	NA	NA	NA	NA
22NRWMX130520	GER-CW		unknown	CVUA-Muenster	51.985558	7.634976	estimated	*Lepus europaeus*	NA	0	0	1	NA	0	0	1	NA	NA	NA	NA	NA	NA	NA	NA	NA
23NRWMX180520	GER-CW		unknown	CVUA-Muenster	51.985558	7.634976	estimated	*Lepus europaeus*	NA	0	0	1	NA	0	0	1	NA	NA	NA	NA	NA	NA	NA	NA	NA
24NRWMX180520	GER-CW		unknown	CVUA-Muenster	51.985558	7.634976	estimated	*Lepus europaeus*	NA	0	0	1	NA	0	0	1	NA	NA	NA	NA	NA	NA	NA	NA	NA
25NRWMX010920	GER-CW		unknown	CVUA-Muenster	51.985558	7.634976	estimated	*Lepus europaeus*	**ON089439**	1	0	0	OM939693	1	0	0	**OM990933**	GGAGGCAAAGGTGGAGGC	GGKGGG	AAAGGAGGC	KGG	NA	2	AAA	K
26NRWFX010920	GER-CW		unknown	CVUA-Muenster	51.985558	7.634976	estimated	*Lepus europaeus*	NA	0	0	1	NA	0	0	1	NA	NA	NA	NA	NA	NA	NA	NA	NA
27NRWFX300920	GER-CW		unknown	CVUA-Muenster	51.985558	7.634976	estimated	*Lepus europaeus*	**ON089441**	1	0	0	OM939693	0	1	0	NA	NA	NA	NA	NA	NA	NA	NA	NA
28NRWMX011020	GER-CW		unknown	CVUA-Muenster	51.985558	7.634976	estimated	*Lepus europaeus*	NA	0	0	1	NA	0	0	1	NA	NA	NA	NA	NA	NA	NA	NA	NA
29NRWMX061020	GER-CW		unknown	CVUA-Muenster	51.985558	7.634976	estimated	*Lepus europaeus*	NA	0	0	1	NA	0	0	1	NA	NA	NA	NA	NA	NA	NA	NA	NA
30NRWMX091020	GER-CW		unknown	CVUA-Muenster	51.985558	7.634976	estimated	*Lepus europaeus*	NA	0	0	1	NA	0	0	1	NA	NA	NA	NA	NA	NA	NA	NA	NA
31NRWFX141020	GER-CW		unknown	CVUA-Muenster	51.985558	7.634976	estimated	*Lepus europaeus*	NA	0	0	1	NA	0	0	1	NA	NA	NA	NA	NA	NA	NA	NA	NA
32NRWMX301020	GER-CW		unknown	CVUA-Muenster	51.985558	7.634976	estimated	*Lepus europaeus*	**ON089442**	1	0	0	**OM939717**	0	1	0	NA	NA	NA	NA	NA	NA	NA	NA	NA
33NRWMX261020	GER-CW		unknown	CVUA-Muenster	51.985558	7.634976	estimated	*Lepus europaeus*	NA	0	0	1	NA	0	0	1	NA	NA	NA	NA	NA	NA	NA	NA	NA
34NRWFX291020	GER-CW		unknown	CVUA-Muenster	51.985558	7.634976	estimated	*Lepus europaeus*	NA	0	0	1	NA	0	0	1	NA	NA	NA	NA	NA	NA	NA	NA	NA
35NRWMX031120	GER-CW		unknown	CVUA-Muenster	51.985558	7.634976	estimated	*Lepus europaeus*	NA	0	0	1	NA	0	0	1	NA	NA	NA	NA	NA	NA	NA	NA	NA
36NRWFX021120	GER-CW		unknown	CVUA-Muenster	51.985558	7.634976	estimated	*Lepus europaeus*	NA	0	0	1	NA	0	0	1	NA	NA	NA	NA	NA	NA	NA	NA	NA
37NRWMX091120	GER-CW		unknown	CVUA-Muenster	51.985558	7.634976	estimated	*Lepus europaeus*	**ON089443**	1	0	0	OM939697	0	1	0	NA	NA	NA	NA	NA	NA	NA	NA	NA
38NRWMX121120	GER-CW		unknown	CVUA-Muenster	51.985558	7.634976	estimated	*Lepus europaeus*	NA	0	0	1	NA	0	0	1	NA	NA	NA	NA	NA	NA	NA	NA	NA
39NRWFX171120	GER-CW		unknown	CVUA-Muenster	51.985558	7.634976	estimated	*Lepus europaeus*	NA	0	0	1	NA	0	0	1	NA	NA	NA	NA	NA	NA	NA	NA	NA
40NRWFX181120	GER-CW		unknown	CVUA-Muenster	51.985558	7.634976	estimated	*Lepus europaeus*	NA	0	0	1	NA	0	0	1	NA	NA	NA	NA	NA	NA	NA	NA	NA
41NRWMX241120	GER-CW		unknown	CVUA-Muenster	51.985558	7.634976	estimated	*Lepus europaeus*	NA	0	0	1	NA	0	0	1	NA	NA	NA	NA	NA	NA	NA	NA	NA
42NRWMX241120	GER-CW		unknown	CVUA-Muenster	51.985558	7.634976	estimated	*Lepus europaeus*	**ON089449**	0	1	0	NA	0	1	0	NA	NA	NA	NA	NA	NA	NA	NA	NA
43NRWMX241120	GER-CW		unknown	CVUA-Muenster	51.985558	7.634976	estimated	*Lepus europaeus*	NA	0	0	1	NA	0	0	1	NA	NA	NA	NA	NA	NA	NA	NA	NA
44NRWMX231120	GER-CW		unknown	CVUA-Muenster	51.985558	7.634976	estimated	*Lepus europaeus*	NA	0	0	1	NA	0	0	1	NA	NA	NA	NA	NA	NA	NA	NA	NA
45NRWMX100320	GER-CW		unknown	CVUA-Krefeld	51.32395	6.55724	estimated	*Lepus europaeus*	**ON089453**	1	0	0	**OM939720**	1	0	0	**OM990963**	GGAGGC	GG	AAAGGTGGAGGC	KGGG	NA	2	AAAGGAGGCAAA	KGGK
46NRWMX240320	GER-CW		unknown	CVUA-Krefeld	51.32395	6.55724	estimated	*Lepus europaeus*	NA	0	0	1	NA	0	0	1	NA	NA	NA	NA	NA	NA	NA	NA	NA
47NRWMX240320	GER-CW		unknown	CVUA-Krefeld	51.32395	6.55724	estimated	*Lepus europaeus*	**ON089454**	1	0	0	OM939694	1	0	0	**OM990965**	NA	NA	AAAGGCAAAGGT	KGKG	II	2	AAAGGCAAA	KGK
48NRWMX060420	GER-CW		unknown	CVUA-Krefeld	51.32395	6.55724	estimated	*Lepus europaeus*	NA	0	0	1	NA	0	0	1	NA	NA	NA	NA	NA	NA	NA	NA	NA
49NRWFX060420	GER-CW		unknown	CVUA-Krefeld	51.32395	6.55724	estimated	*Oryctolagus cuniculus*	NA	0	0	1	NA	0	0	1	NA	NA	NA	NA	NA	NA	NA	NA	NA
50NRWMX160420	GER-CW		unknown	CVUA-Krefeld	51.32395	6.55724	estimated	*Lepus europaeus*	NA	0	0	1	NA	0	0	1	NA	NA	NA	NA	NA	NA	NA	NA	NA
51NRWFX240420	GER-CW		unknown	CVUA-Krefeld	51.32395	6.55724	estimated	*Lepus europaeus*	NA	0	0	1	NA	0	0	1	NA	NA	NA	NA	NA	NA	NA	NA	NA

52NRWMX040520	GER-CW		unknown	CVUA-Krefeld	51.32395	6.55724	estimated	*Lepus europaeus*	NA	0	0	1	NA	0	0	1	NA	NA	NA	NA	NA	NA	NA	NA	NA
53NRWMX040520	GER-CW		unknown	CVUA-Krefeld	51.32395	6.55724	estimated	*Lepus europaeus*	NA	0	0	1	NA	0	0	1	NA	NA	NA	NA	NA	NA	NA	NA	NA
54NRWFX020620	GER-CW		unknown	CVUA-Krefeld	51.32395	6.55724	estimated	*Lepus europaeus*	**ON089462**	1	0	0	OM939693	0	1	0	NA	NA	NA	NA	NA	NA	NA	NA	NA
55NRWMX030720	GER-CW		unknown	CVUA-Krefeld	51.32395	6.55724	estimated	*Lepus europaeus*	NA	0	0	1	NA	0	0	1	NA	NA	NA	NA	NA	NA	NA	NA	NA
56NRWMX080720	GER-CW		unknown	CVUA-Krefeld	51.32395	6.55724	estimated	*Lepus europaeus*	NA	0	0	1	NA	0	0	1	NA	NA	NA	NA	NA	NA	NA	NA	NA
57NRWMX050820	GER-CW		unknown	CVUA-Krefeld	51.32395	6.55724	estimated	*Oryctolagus cuniculus*	NA	0	0	1	NA	0	0	1	NA	NA	NA	NA	NA	NA	NA	NA	NA
58NRWFX060820	GER-CW		unknown	CVUA-Krefeld	51.32395	6.55724	estimated	*Oryctolagus cuniculus*	NA	0	0	1	NA	0	0	1	NA	NA	NA	NA	NA	NA	NA	NA	NA
59NRWFX200820	GER-CW		unknown	CVUA-Krefeld	51.32395	6.55724	estimated	*Lepus europaeus*	NA	0	0	1	NA	0	0	1	NA	NA	NA	NA	NA	NA	NA	NA	NA
60NRWMX240820	GER-CW		unknown	CVUA-Krefeld	51.32395	6.55724	estimated	*Lepus europaeus*	NA	0	0	1	NA	0	0	1	NA	NA	NA	NA	NA	NA	NA	NA	NA
61NRWMX280820	GER-CW		unknown	CVUA-Krefeld	51.32395	6.55724	estimated	*Lepus europaeus*	**ON089471**	1	0	0	OM939693	1	0	0	**OM990981**	GGAGGC	GG	AAAGGTGGAGGC	KGGG	I	2	AAAGGCAAA	KGK
62NRWFX020920	GER-CW		unknown	CVUA-Krefeld	51.32395	6.55724	estimated	*Lepus europaeus*	NA	0	0	1	NA	0	0	1	NA	NA	NA	NA	NA	NA	NA	NA	NA
63NRWMX020920	GER-CW		unknown	CVUA-Krefeld	51.32395	6.55724	estimated	*Lepus europaeus*	NA	0	0	1	NA	0	0	1	NA	NA	NA	NA	NA	NA	NA	NA	NA
64NRWMX040920	GER-CW		unknown	CVUA-Krefeld	51.32395	6.55724	estimated	*Lepus europaeus*	**ON089477**	1	0	0	OM939696	1	0	0	**OM990986**	GGAGGC	GG	AAAGGTGGAGGC	KGGG	I	2	AAA	K
65NRWFX080920	GER-CW		unknown	CVUA-Krefeld	51.32395	6.55724	estimated	*Lepus europaeus*	**ON089479**	1	0	0	OM939693	1	0	0	**OM990989**	GGAGGC	GG	AAAGGTGGAGGC	KGGG	I	2	AAA	K
66NRWFX080920	GER-CW		unknown	CVUA-Krefeld	51.32395	6.55724	estimated	*Lepus europaeus*	NA	0	0	1	NA	0	0	1	NA	NA	NA	NA	NA	NA	NA	NA	NA
67NRWMX080920	GER-CW		unknown	CVUA-Krefeld	51.32395	6.55724	estimated	*Lepus europaeus*	NA	0	0	1	NA	0	0	1	NA	NA	NA	NA	NA	NA	NA	NA	NA
68NRWMX150920	GER-CW		unknown	CVUA-Krefeld	51.32395	6.55724	estimated	*Lepus europaeus*	NA	0	0	1	NA	0	0	1	NA	NA	NA	NA	NA	NA	NA	NA	NA
69NRWFX170920	GER-CW		unknown	CVUA-Krefeld	51.32395	6.55724	estimated	*Lepus europaeus*	NA	0	0	1	NA	0	0	1	NA	NA	NA	NA	NA	NA	NA	NA	NA
70NRWMX230920	GER-CW		unknown	CVUA-Krefeld	51.32395	6.55724	estimated	*Lepus europaeus*	NA	0	0	1	NA	0	0	1	NA	NA	NA	NA	NA	NA	NA	NA	NA
71NRWFX191020	GER-CW		unknown	CVUA-Krefeld	51.32395	6.55724	estimated	*Lepus europaeus*	NA	0	0	1	NA	0	0	1	NA	NA	NA	NA	NA	NA	NA	NA	NA
72NRWMX201020	GER-CW		unknown	CVUA-Krefeld	51.32395	6.55724	estimated	*Oryctolagus cuniculus*	NA	0	0	1	NA	0	0	1	NA	NA	NA	NA	NA	NA	NA	NA	NA
73NRWMX301020	GER-CW		unknown	CVUA-Krefeld	51.32395	6.55724	estimated	*Lepus europaeus*	NA	0	0	1	NA	0	0	1	NA	NA	NA	NA	NA	NA	NA	NA	NA
74NRWMX111120	GER-CW		unknown	CVUA-Krefeld	51.32395	6.55724	estimated	*Lepus europaeus*	**ON089494**	1	0	0	OM939693	1	0	0	**OM991000**	GGAGGC	GG	AAAGGTGGAGGC	KGGG	I	2	AAA	K
75NRWFX181120	GER-CW		unknown	CVUA-Krefeld	51.32395	6.55724	estimated	*Lepus europaeus*	NA	0	0	1	NA	0	0	1	NA	NA	NA	NA	NA	NA	NA	NA	NA
76NRWFX261120	GER-CW		unknown	CVUA-Krefeld	51.32395	6.55724	estimated	*Lepus europaeus*	**ON089498**	1	0	0	OM939694	0	1	0	NA	NA	NA	NA	NA	NA	NA	NA	NA
77NRWFX271120	GER-CW		unknown	CVUA-Krefeld	51.32395	6.55724	estimated	*Lepus europaeus*	ON089569	0	1	0	NA	1	0	0	**OM991002**	GTAGGCAAAGGTGTAGGC	VGKGVG	AAAGGTGGAGGC	KGGG	NA	2	AAA	K
78NRWMX301120	GER-CW		unknown	CVUA-Krefeld	51.32395	6.55724	estimated	*Lepus europaeus*	NA	0	0	1	NA	0	0	1	NA	NA	NA	NA	NA	NA	NA	NA	NA
79NRWMX071220	GER-CW		unknown	CVUA-Krefeld	51.32395	6.55724	estimated	*Lepus europaeus*	**ON089506**	0	1	0	NA	1	0	0	**OM991006**	GGAGGC	GG	AAAGGTGGAGGC	KGGG	I	1	AAA	K
80NRWFX091220	GER-CW		unknown	CVUA-Krefeld	51.32395	6.55724	estimated	*Lepus europaeus*	NA	0	0	1	NA	0	0	1	NA	NA	NA	NA	NA	NA	NA	NA	NA
81NRWFX130121	GER-CW		unknown	CVUA-Arnsberg	51.384394	8.077302	estimated	*Lepus europaeus*	NA	0	0	1	NA	0	0	1	NA	NA	NA	NA	NA	NA	NA	NA	NA
82NRWFX130121	GER-CW		unknown	CVUA-Arnsberg	51.384394	8.077302	estimated	*Lepus europaeus*	NA	0	0	1	NA	0	0	1	NA	NA	NA	NA	NA	NA	NA	NA	NA
83NRWMX130121	GER-CW		unknown	CVUA-Arnsberg	51.384394	8.077302	estimated	*Lepus europaeus*	**ON089511**	1	0	0	OM939700	0	1	0	NA	NA	NA	NA	NA	NA	NA	NA	NA
84NRWMX130121	GER-CW		unknown	CVUA-Arnsberg	51.384394	8.077302	estimated	*Lepus europaeus*	NA	0	0	1	NA	0	0	1	NA	NA	NA	NA	NA	NA	NA	NA	NA
85NRWFX130121	GER-CW		unknown	CVUA-Arnsberg	51.384394	8.077302	estimated	*Lepus europaeus*	NA	0	0	1	NA	0	0	1	NA	NA	NA	NA	NA	NA	NA	NA	NA
86NRWFX130121	GER-CW		unknown	CVUA-Arnsberg	51.384394	8.077302	estimated	*Lepus europaeus*	NA	0	0	1	NA	0	0	1	NA	NA	NA	NA	NA	NA	NA	NA	NA
87NRWFX130121	GER-CW		unknown	CVUA-Arnsberg	51.384394	8.077302	estimated	*Oryctolagus cuniculus*	NA	0	0	1	NA	0	0	1	NA	NA	NA	NA	NA	NA	NA	NA	NA
88NRWFX130121	GER-CW		unknown	CVUA-Arnsberg	51.384394	8.077302	estimated	*Oryctolagus cuniculus*	NA	0	0	1	NA	0	0	1	NA	NA	NA	NA	NA	NA	NA	NA	NA
89NRWFX130121	GER-CW		unknown	CVUA-Arnsberg	51.384394	8.077302	estimated	*Oryctolagus cuniculus*	NA	0	0	1	NA	0	0	1	NA	NA	NA	NA	NA	NA	NA	NA	NA
90NRWFX130121	GER-CW		unknown	CVUA-Arnsberg	51.384394	8.077302	estimated	*Oryctolagus cuniculus*	NA	0	0	1	NA	0	0	1	NA	NA	NA	NA	NA	NA	NA	NA	NA
91NRWFX130121	GER-CW		unknown	CVUA-Arnsberg	51.384394	8.077302	estimated	*Lepus europaeus*	NA	0	0	1	NA	0	0	1	NA	NA	NA	NA	NA	NA	NA	NA	NA
92NRWFX130121	GER-CW		unknown	CVUA-Arnsberg	51.384394	8.077302	estimated	*Oryctolagus cuniculus*	NA	0	0	1	NA	0	0	1	NA	NA	NA	NA	NA	NA	NA	NA	NA
93NRWMX130121	GER-CW		unknown	CVUA-Arnsberg	51.384394	8.077302	estimated	*Lepus europaeus*	**ON089518**	1	0	0	**OM939704**	0	1	0	NA	NA	NA	NA	NA	NA	NA	NA	NA
94NRWFX130121	GER-CW		unknown	CVUA-Arnsberg	51.384394	8.077302	estimated	*Lepus europaeus*	NA	0	0	1	NA	0	0	1	NA	NA	NA	NA	NA	NA	NA	NA	NA
95NRWFX130121	GER-CW		unknown	CVUA-Arnsberg	51.384394	8.077302	estimated	*Lepus europaeus*	NA	0	0	1	NA	0	0	1	NA	NA	NA	NA	NA	NA	NA	NA	NA
96NRWMX130121	GER-CW		unknown	CVUA-Arnsberg	51.384394	8.077302	estimated	*Lepus europaeus*	NA	0	0	1	NA	0	0	1	NA	NA	NA	NA	NA	NA	NA	NA	NA
97NRWFX130121	GER-CW		unknown	CVUA-Arnsberg	51.384394	8.077302	estimated	*Lepus europaeus*	NA	0	0	1	NA	0	0	1	NA	NA	NA	NA	NA	NA	NA	NA	NA
98NRWFX250320	GER-CW		unknown	CVUA-Detmold	51.95655	8.84611	estimated	*Oryctolagus cuniculus f. domestica*	NA	0	0	1	NA	0	0	1	NA	NA	NA	NA	NA	NA	NA	NA	NA
99NRWFX260320	GER-CW		unknown	CVUA-Detmold	51.95655	8.84611	estimated	*Oryctolagus cuniculus f. domestica*	NA	0	0	1	NA	0	0	1	NA	NA	NA	NA	NA	NA	NA	NA	NA
100NRWFX010420	GER-CW		unknown	CVUA-Detmold	51.95655	8.84611	estimated	*Oryctolagus cuniculus*	NA	0	0	1	NA	0	0	1	NA	NA	NA	NA	NA	NA	NA	NA	NA
101NRWMX300720	GER-CW		unknown	CVUA-Detmold	51.95655	8.84611	estimated	*Lepus europaeus*	NA	0	0	1	NA	0	0	1	NA	NA	NA	NA	NA	NA	NA	NA	NA
102NRWFX050820	GER-CW		unknown	CVUA-Detmold	51.95655	8.84611	estimated	*Lepus europaeus*	NA	0	0	1	NA	0	0	1	NA	NA	NA	NA	NA	NA	NA	NA	NA
103NRWFX220920	GER-CW		unknown	CVUA-Detmold	51.95655	8.84611	estimated	*Lepus europaeus*	NA	0	0	1	NA	0	0	1	NA	NA	NA	NA	NA	NA	NA	NA	NA
104NRWMX280920	GER-CW		unknown	CVUA-Detmold	51.95655	8.84611	estimated	*Lepus europaeus*	NA	0	0	1	NA	0	0	1	NA	NA	NA	NA	NA	NA	NA	NA	NA
105NRWMX051120	GER-CW		unknown	CVUA-Detmold	51.95655	8.84611	estimated	*Lepus europaeus*	NA	0	0	1	NA	0	0	1	NA	NA	NA	NA	NA	NA	NA	NA	NA
106NRWFX041220	GER-CW		unknown	CVUA-Detmold	51.95655	8.84611	estimated	*Lepus europaeus*	NA	0	0	1	NA	0	0	1	NA	NA	NA	NA	NA	NA	NA	NA	NA
107NRWMX271120	GER-CW		unknown	Monheim	51.082516	6.872097	estimated	*Lepus europaeus*	**ON089532**	0	0	1	NA	0	1	0	NA	NA	NA	NA	NA	NA	NA	NA	NA
108NRWFX271120	GER-CW		unknown	Monheim	51.082516	6.872097	estimated	*Lepus europaeus*	NA	0	0	1	NA	0	0	1	NA	NA	NA	NA	NA	NA	NA	NA	NA
109NRWFX271120	GER-CW		unknown	Monheim	51.082516	6.872097	estimated	*Lepus europaeus*	**ON089535**	1	0	0	**OM939715**	1	0	0	**OM991033**	GGAGGC	GG	AAAGGTGGAGGC	KGGG	I	2	AAA	K
110NRWFX271120	GER-CW		unknown	Monheim	51.082516	6.872097	estimated	*Lepus europaeus*	NA	0	0	1	NA	0	0	1	NA	NA	NA	NA	NA	NA	NA	NA	NA
111NRWMX271120	GER-CW		unknown	Monheim	51.082516	6.872097	estimated	*Lepus europaeus*	NA	0	0	1	NA	0	0	1	NA	NA	NA	NA	NA	NA	NA	NA	NA
112NRWFX271120	GER-CW		unknown	Monheim	51.082516	6.872097	estimated	*Lepus europaeus*	NA	0	0	1	NA	0	0	1	NA	NA	NA	NA	NA	NA	NA	NA	NA
113NRWFX271120	GER-CW		unknown	Monheim	51.082516	6.872097	estimated	*Lepus europaeus*	NA	0	0	1	NA	0	0	1	NA	NA	NA	NA	NA	NA	NA	NA	NA
114NRWMX271120	GER-CW		unknown	Monheim	51.082516	6.872097	estimated	*Lepus europaeus*	NA	0	0	1	NA	0	0	1	NA	NA	NA	NA	NA	NA	NA	NA	NA
115NRWMX271120	GER-CW		unknown	Monheim	51.082516	6.872097	estimated	*Lepus europaeus*	NA	0	0	1	NA	0	0	1	NA	NA	NA	NA	NA	NA	NA	NA	NA
116NRWFX271120	GER-CW		unknown	Monheim	51.082516	6.872097	estimated	*Lepus europaeus*	NA	0	0	1	NA	0	0	1	NA	NA	NA	NA	NA	NA	NA	NA	NA
117NRWMX271120	GER-CW		unknown	Monheim	51.082516	6.872097	estimated	*Lepus europaeus*	NA	0	0	1	NA	0	0	1	NA	NA	NA	NA	NA	NA	NA	NA	NA
118NRWMX271120	GER-CW		unknown	Monheim	51.082516	6.872097	estimated	*Lepus europaeus*	NA	0	0	1	NA	0	0	1	NA	NA	NA	NA	NA	NA	NA	NA	NA
119NRWMX271120	GER-CW		unknown	Monheim	51.082516	6.872097	estimated	*Lepus europaeus*	NA	0	0	1	NA	0	0	1	NA	NA	NA	NA	NA	NA	NA	NA	NA
120NRWMX121220	GER-CW		unknown	Heimerzheim	50.705302	6.909716	estimated	*Lepus europaeus*	NA	0	0	1	NA	0	0	1	NA	NA	NA	NA	NA	NA	NA	NA	NA
121NRWFX121220	GER-CW		unknown	Heimerzheim	50.705302	6.909716	estimated	*Lepus europaeus*	NA	0	0	1	NA	0	0	1	NA	NA	NA	NA	NA	NA	NA	NA	NA
122NRWMX121220	GER-CW		unknown	Heimerzheim	50.705302	6.909716	estimated	*Lepus europaeus*	NA	0	0	1	NA	0	0	1	NA	NA	NA	NA	NA	NA	NA	NA	NA
123NRWMX121220	GER-CW		unknown	Heimerzheim	50.705302	6.909716	estimated	*Lepus europaeus*	NA	0	0	1	NA	0	0	1	NA	NA	NA	NA	NA	NA	NA	NA	NA
124NRWFX121220	GER-CW		unknown	Heimerzheim	50.705302	6.909716	estimated	*Lepus europaeus*	NA	0	0	1	NA	0	0	1	NA	NA	NA	NA	NA	NA	NA	NA	NA
125NRWFX121220	GER-CW		unknown	Heimerzheim	50.705302	6.909716	estimated	*Lepus europaeus*	NA	0	0	1	NA	0	0	1	NA	NA	NA	NA	NA	NA	NA	NA	NA
126NRWFX121220	GER-CW		unknown	Heimerzheim	50.705302	6.909716	estimated	*Lepus europaeus*	NA	0	0	1	NA	0	0	1	NA	NA	NA	NA	NA	NA	NA	NA	NA
127NRWFX121220	GER-CW		unknown	Heimerzheim	50.705302	6.909716	estimated	*Lepus europaeus*	**ON089553**	0	1	0	NA	1	0	0	**OM991052**	AGAGGC	RG	AAAGGTGGAGGC	KGGG	I	3	AAA	K
128NRWFX121220	GER-CW		unknown	Heimerzheim	50.705302	6.909716	estimated	*Lepus europaeus*	NA	0	0	1	NA	0	0	1	NA	NA	NA	NA	NA	NA	NA	NA	NA
129NRWMX121220	GER-CW		unknown	Heimerzheim	50.705302	6.909716	estimated	*Lepus europaeus*	NA	0	0	1	NA	0	0	1	NA	NA	NA	NA	NA	NA	NA	NA	NA
130NRWFX121220	GER-CW		unknown	Heimerzheim	50.705302	6.909716	estimated	*Lepus europaeus*	**ON089554**	0	1	0	NA	1	0	0	**OM991055**	GGAGGC	GG	AAAGGTGGAGGC	KGGG	I	3	AAA	K
131NRWMX121220	GER-CW		unknown	Heimerzheim	50.705302	6.909716	estimated	*Lepus europaeus*	NA	0	0	1	NA	0	0	1	NA	NA	NA	NA	NA	NA	NA	NA	NA
132NRWFX121220	GER-CW		unknown	Heimerzheim	50.705302	6.909716	estimated	*Lepus europaeus*	NA	0	0	1	NA	0	0	1	NA	NA	NA	NA	NA	NA	NA	NA	NA
133NRWMX121220	GER-CW		unknown	Heimerzheim	50.705302	6.909716	estimated	*Lepus europaeus*	NA	0	0	1	NA	0	0	1	NA	NA	NA	NA	NA	NA	NA	NA	NA
134NRWFX121220	GER-CW		unknown	Heimerzheim	50.705302	6.909716	estimated	*Lepus europaeus*	NA	0	0	1	NA	0	0	1	NA	NA	NA	NA	NA	NA	NA	NA	NA
135NRWFX121220	GER-CW		unknown	Heimerzheim	50.705302	6.909716	estimated	*Lepus europaeus*	NA	0	0	1	NA	0	0	1	NA	NA	NA	NA	NA	NA	NA	NA	NA
136NRWMX121220	GER-CW		unknown	Heimerzheim	50.705302	6.909716	estimated	*Lepus europaeus*	NA	0	0	1	NA	0	0	1	NA	NA	NA	NA	NA	NA	NA	NA	NA
137NRWFX121220	GER-CW		unknown	Heimerzheim	50.705302	6.909716	estimated	*Lepus europaeus*	NA	0	0	1	NA	0	0	1	NA	NA	NA	NA	NA	NA	NA	NA	NA
138NRWMX121220	GER-CW		unknown	Heimerzheim	50.705302	6.909716	estimated	*Lepus europaeus*	NA	0	0	1	NA	0	0	1	NA	NA	NA	NA	NA	NA	NA	NA	NA
139NRWFX121220	GER-CW		unknown	Heimerzheim	50.705302	6.909716	estimated	*Lepus europaeus*	NA	0	0	1	NA	0	0	1	NA	NA	NA	NA	NA	NA	NA	NA	NA
140NRWMX121220	GER-CW		unknown	Heimerzheim	50.705302	6.909716	estimated	*Lepus europaeus*	NA	0	0	1	NA	0	0	1	NA	NA	NA	NA	NA	NA	NA	NA	NA
141NRWFX121220	GER-CW		unknown	Heimerzheim	50.705302	6.909716	estimated	*Lepus europaeus*	NA	0	0	1	NA	0	0	1	NA	NA	NA	NA	NA	NA	NA	NA	NA
142NRWMX121220	GER-CW		unknown	Heimerzheim	50.705302	6.909716	estimated	*Lepus europaeus*	NA	0	0	1	NA	0	0	1	NA	NA	NA	NA	NA	NA	NA	NA	NA
