# Supplementary material for: High prevalence and genetic diversity of Treponema paraluisleporidarum isolates in European lagomorphs
Source: Microbiol Spectr. 2023 Dec 14;12(1):e01774-23. doi: 10.1128/spectrum.01774-23 (PMC10783078; doi:10.1128/spectrum.01774-23)
Supplement: Table S2 — GenBank accession numbers for the mt-genomes. [file spectrum.01774-23-s0003.pdf]

Sample ID	Country Code	Field Site	Species	Gene Bank Accession Number
03ITUX140319	IT	Roverbella	*Lepus europaeus*	OM993367
04ITFX140319	IT	Zocca	*Lepus europaeus*	OM993373
69NLF1281118	NL	Wilp	*Lepus europaeus*	OM993436
78NLM1281118	NL	Wilp	*Lepus europaeus*	OM993441
80NLF1281118	NL	Wilp	*Lepus europaeus*	OM993442
82NLF1281118	NL	Wilp	*Lepus europaeus*	OM993443
84NLM1281118	NL	Wilp	*Lepus europaeus*	OM993444
96NLFX090119	NL	Utrecht	*Lepus europaeus*	OM993446
97NLFX090119	NL	Utrecht	*Lepus europaeus*	OM993447
98NLMX290519	NL	Utrecht	*Lepus europaeus*	OM993448
01GBMX060219	GB	Somerset	*Lepus europaeus*	OM993362
02GBFX010319	GB	Hampshire	*Lepus europaeus*	OM993361
03GBFX020319	GB	Wiltshire	*Lepus europaeus*	OM993366
04GBFX030319	GB	Wiltshire	*Lepus europaeus*	OM993372
05GBMX140819	GB	Dorset	*Lepus europaeus*	OM993376
06GBFX140619	GB	Wiltshire	*Lepus europaeus*	OM993380
07GBMX040719	GB	Gloucestershire	*Lepus europaeus*	OM993382
08GBMX160719	GB	Devon	*Lepus europaeus*	OM993385
06CZM1140919	CZ	Brumovice	*Lepus europaeus*	OM993379
22CZF1150919	CZ	Lhoty u Potštejna	*Lepus europaeus*	OM993418
32CZM1150919	CZ	Lhoty u Potštejna	*Lepus europaeus*	OM993426
38CZF1150919	CZ	Lhoty u Potštejna	*Lepus europaeus*	OM993432
55CZM1150919	CZ	Lhoty u Potštejna	*Lepus europaeus*	OM993435
07BVM2131118	GER	Bad Vilbel	*Lepus europaeus*	OM993381
28BVF1131118	GER	Bad Vilbel	*Lepus europaeus*	OM993424
40BVM1131118	GER	Bad Vilbel	*Lepus europaeus*	OM993433
95BVM1121119	GER	Bad Vilbel	*Lepus europaeus*	OM993445
111BVM1131119	GER	Bad Vilbel	*Lepus europaeus*	OM993393
129BVM1201119	GER	Niedererlenbach	*Lepus europaeus*	OM993397
143BVF1201119	GER	Niedererlenbach	*Lepus europaeus*	OM993405
102EBF1311218	GER	Hullersen	*Lepus europaeus*	OM993389
108EBF1311218	GER	Hullersen	*Lepus europaeus*	OM993390
130EBF1301219	GER	Hullersen	*Lepus europaeus*	OM993401
131EBM1301219	GER	Hullersen	*Lepus europaeus*	OM993402
02BYFX151019	GER	Heuberg bei Hilpoltstein	*Lepus europaeus*	OM993363
21BYM1281219	GER	Großeibstadt	*Lepus europaeus*	OM993415
25BYF1281219	GER	Großeibstadt	*Lepus europaeus*	OM993421
34BYM1281219	GER	Großeibstadt	*Lepus europaeus*	OM993428
38BYF1281219	GER	Großeibstadt	*Lepus europaeus*	OM993431
02SHMX270419	GER	Schleswig Holstein	*Lepus europaeus*	OM993365
03SHFX280519	GER	Schleswig Holstein	*Lepus europaeus*	OM993369
12SHMX151019	GER	Schleswig Holstein	*Lepus europaeus*	OM993400
71SHF1161119	GER	Föhr	*Lepus europaeus*	OM993437
72SHF1161119	GER	Föhr	*Lepus europaeus*	OM993438
75SHM1161119	GER	Föhr	*Lepus europaeus*	OM993439
76SHM1161119	GER	Föhr	*Lepus europaeus*	OM993440
109SHF1111219	GER	Fehmarn	*Lepus europaeus*	OM993391
110SHF1111219	GER	Fehmarn	*Lepus europaeus*	OM993392
121SHF1131219	GER	Fehmarn	*Lepus europaeus*	OM993396
01KSMX021119	GER	Espenau	*Lepus europaeus*	OM993359
02KSFX021119	GER	Espenau	*Lepus europaeus*	OM993364
03SOF1161119	GER	Geseke	*Lepus europaeus*	OM993370
04SOF1161119	GER	Geseke	*Lepus europaeus*	OM993374
09SOM1161119	GER	Geseke	*Lepus europaeus*	OM993388
15SOF1161119	GER	Geseke	*Lepus europaeus*	OM993410
16SOM1161119	GER	Geseke	*Lepus europaeus*	OM993411
01LRM1231119	GER	Mahlberg	*Lepus europaeus*	OM993354
07LRF1231119	GER	Mahlberg	*Lepus europaeus*	OM993383
12LRM1231119	GER	Mahlberg	*Lepus europaeus*	OM993399
13LRM1231119	GER	Mahlberg	*Lepus europaeus*	OM993404
01FZM1301119	GER	Fritzlar	*Lepus europaeus*	OM993356
13FZM1301119	GER	Fritzlar	*Lepus europaeus*	OM993403
14FZF1301119	GER	Fritzlar	*Lepus europaeus*	OM993406
17FZF1301119	GER	Fritzlar	*Lepus europaeus*	OM993412
02FBM1041219	GER	Nieder-Wöllstadt	*Lepus europaeus*	OM993357
19FBF1041219	GER	Nieder-Wöllstadt	*Lepus europaeus*	OM993414
21FBM1041219	GER	Nieder-Wöllstadt	*Lepus europaeus*	OM993416
34FBM1041219	GER	Nieder-Wöllstadt	*Lepus europaeus*	OM993429
04AUF1061219	GER	Riepe	*Lepus europaeus*	OM993371
12AUM1061219	GER	Riepe	*Lepus europaeus*	OM993398
24AUM1061219	GER	Riepe	*Lepus europaeus*	OM993419
31AUF1061219	GER	Riepe	*Lepus europaeus*	OM993425
54AUM1061219	GER	Riepe	*Lepus europaeus*	OM993434
03IZM1071219	GER	Nienborstel	*Lepus europaeus*	OM993368
14IZF1071219	GER	Nienborstel	*Lepus europaeus*	OM993407
21IZF1071219	GER	Jahrsdorf	*Lepus europaeus*	OM993417
26IZM1071219	GER	Jahrsdorf	*Lepus europaeus*	OM993422
33IZM1071219	GER	Nienborstel	*Lepus europaeus*	OM993427
36IZF1071219	GER	Nienborstel	*Lepus europaeus*	OM993430
05PEM1181219	GER	Bierbergen	*Lepus europaeus*	OM993378
07PEM1181219	GER	Bierbergen	*Lepus europaeus*	OM993384
08PEF1181219	GER	Bierbergen	*Lepus europaeus*	OM993387
15PEF1181219	GER	Bierbergen	*Lepus europaeus*	OM993409
02GÖM1191219	GER	Reinshof	*Lepus europaeus*	OM993358
05GÖF1191219	GER	Reinshof	*Lepus europaeus*	OM993377
08GÖF1191219	GER	Reinshof	*Lepus europaeus*	OM993386
15GÖM1301219	GER	Parensen	*Lepus europaeus*	OM993408
27GÖF1301219	GER	Parensen	*Lepus europaeus*	OM993423
01BWFX051019	GER	Bad Krozingen-Biengen	*Lepus europaeus*	OM993360
11BWFX051019	GER	Bad Krozingen-Biengen	*Lepus europaeus*	OM993394
19BWMX281219	GER	Tunsel Ost	*Lepus europaeus*	OM993413
24BWMX281219	GER	Tunsel Ost	*Lepus europaeus*	OM993420
01CPMX081119	GER	Hochelsten	*Lepus europaeus*	OM993355
05CPFX081119	GER	Hochelsten	*Lepus europaeus*	OM993375
11CPMX091119	GER	Siehenfelde	*Lepus europaeus*	OM993395
